# From Herbal Tea to Science: Phytochemical and Biological Investigation of *Ajuga chamaepitys* subsp. *chia* (Lamiaceae) With Molecular Docking Analysis

**DOI:** 10.1002/fsn3.70749

**Published:** 2025-08-13

**Authors:** Bilge Aydın, Amine Sena Aydın, Ömer Çeçen, Hafize Yuca, Gülnur Ekşi Bona, Betül Demirci, Mehmet Bona, Songul Karakaya

**Affiliations:** ^1^ Department of Pharmacognosy, Faculty of Pharmacy Erzincan Binali Yıldırım University Erzincan Türkiye; ^2^ Department of Pharmaceutical Chemistry, Faculty of Pharmacy Ataturk University Erzurum Türkiye; ^3^ Department of Biology, Kamil Özdağ Faculty of Science Karamanoğlu Mehmetbey University Karaman Türkiye; ^4^ Department of Pharmacognosy, Faculty of Pharmacy Ataturk University Erzurum Türkiye; ^5^ Department of Pharmaceutical Botany, Faculty of Pharmacy İstanbul‐Cerrahpaşa University İstanbul Türkiye; ^6^ Department of Pharmacognosy, Faculty of Pharmacy Anadolu University Erzurum Türkiye; ^7^ Department of Biology, Faculty of Science İstanbul University İstanbul Türkiye; ^8^ Department of Pharmaceutical Botany, Faculty of Pharmacy Ataturk University Erzurum Türkiye

**Keywords:** *Ajuga chamaepitys* subsp. *chia*, Alzheimer's disease, diabetes, morpho‐anatomy, pinene

## Abstract

The hypothesis that insulin resistance and impaired insulin‐like growth factor signaling contribute to Alzheimer's disease (AD) has led to the designation of AD as “type 3 diabetes.” *Ajuga chamaepitys* subsp. *chia* is traditionally used in Türkiye as an analgesic, tonic, and for the external treatment of hemorrhoids and wound healing. This study evaluates antioxidant, antidiabetic, and anticholinesterase activities of methanolic, aqueous extracts, and essential oils from the flowering aerial parts of *A. chamaepitys* subsp. *chia*, along with their phytochemical profiles (GC–MS/MS), morphological‐anatomical characteristics, and molecular docking analysis. The essential oil yield was 0.002%, comprising 45 compounds (92.5%), with β‐pinene (19.8%), α‐pinene (12.8%), and germacrene D (10.0%) as major constituents, dominated by monoterpene (39.8%) and sesquiterpene hydrocarbons (23.5%). The methanolic extract exhibited moderate bioactivity, achieving 28.36% α‐amylase inhibition at 5000 μg/mL and 21.85% acetylcholinesterase inhibition at 100 μg/mL, while also demonstrating notable antioxidant activity with 23.013% ABTS^•+^ and 8.114% DPPH^•^ scavenging. It showed the highest total phenolic (14.261 μg GAE/mg) and tannin content (34.444 μg TAE/mg) among the tested extracts. Morphologically, the plant features hairy stems and tripartite leaves with a cuticle, trichomes, and diacytic stomata, while anatomically, it presents collenchyma, starch‐filled parenchyma, and a distinct cambium in the stem. Molecular docking studies revealed that germacrene D exhibited strong binding affinities to acetylcholinesterase, butyrylcholinesterase, α‐amylase, and α‐glucosidase, suggesting multitarget inhibition potential. These findings support the traditional use of *A. chamaepitys* subsp. *chia* and highlight its promise as a natural source for therapeutic agents targeting oxidative stress, diabetes, and neurodegenerative diseases.

## Graphical abstract

The present study comprehensively investigates the phytochemical profile, biological activities, and molecular docking interactions of *Ajuga chamaepitys* subsp. chia. Methanolic extracts exhibited notable antioxidant, antidiabetic, and anticholinesterase activities, while germacrene D demonstrated strong multitarget binding affinities in silico. These findings highlight the species' potential as a promising source of bioactive compounds for managing oxidative stress, diabetes, and neurodegenerative diseases.

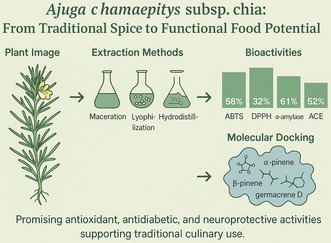

## 1 Introduction

*Ajuga chamaepitys* (L.) Schreb. is a species within the Lamiaceae family, subfamily Ajugoideae, and the genus *Ajuga* L. The *Ajuga* genus comprises approximately 300 species and numerous naturalized subspecies across the globe. *A. chamaepitys* is a small, annual or biennial herbaceous plant that blooms in the summer, specifically from May to August. It has a slender, quadrangular, hairy stem measuring 10–30 cm in length, a rhizome‐shaped root, and 1–4 cm long, tripartite, hairy leaves. The plant's flowers are up to 15 mm long, with a yellow to pale red corolla that has two lips, and its fruit is a tetranucule. When crushed, the entire plant emits a pine scent (Ionus et al. 2021). It has been used in traditional medicine to treat various conditions, including menstrual disorders, as an astringent and antitussive, and for the treatment of hemorrhoids, diabetes, stomachaches, gout, joint pain, and as a painkiller (Jaffal et al. 2019). *A. chamaepitys* is an Euri‐Mediterranean species commonly found in Italy, especially in cultivated fields, urban areas, and ruderal habitats, from mountainous regions down to coastal zones. It often grows behind dunes and along seashores in urbanized landscapes. While it is widespread in agricultural areas as a weed, it is rare in more natural ecosystems (Venditti et al. 2016). According to Selvi et al. (2022), infusion of *A. chamaepitys* subsp. *chia* is used as an analgesic, tonic, and externally for hemorrhoids and wound healing by the people in Türkiye.

Assessing the nutritional and chemical makeup of plants is vital before their use in human or animal diets. Detailed analysis of nutrients and bioactive compounds aids in species verification and provides valuable data for nutritional research. Considering the chemical richness and dietary potential of *Ajuga* species, a thorough investigation of *A. iva*'s mineral content, fatty acids, essential oils, and phenolic profile is essential (Ammar et al. 2022). *A. chamaepitys* subsp. *chia*, commonly known as “Hırtkesen,” has long been used in traditional medicine to relieve pain, cramps, asthma, and colds. In folk practices, its leaves and flowers are typically boiled to make herbal tea, which is consumed to ease discomfort and support respiratory health. This traditional use underscores the plant's importance as a natural remedy for both respiratory and muscular ailments (Aslan et al. 2020). Medicinal plants possess a wide range of pharmacological properties, making them important in both traditional healing and modern drug discovery. Their rich content of bioactive compounds—such as alkaloids, flavonoids, terpenoids, tannins, and phenolics—provides valuable templates for developing treatments against various diseases (İzol 2025).

Alzheimer's Disease (AD) is marked by the accumulation of amyloid‐β (Aβ) plaques outside neurons and tau protein tangles inside them. Aβ buildup may result from increased production, reduced clearance by degrading enzymes, or impaired transport across the blood–brain barrier. Both Type 2 Diabetes Mellitus (T2DM) and dyslipidemia contribute to Aβ accumulation and vascular dysfunction in the brain, making them key targets in AD prevention. T2DM is often associated with dyslipidemia, characterized by high triglycerides, low HDL cholesterol, and small, dense LDL particles (Carlsson 2010).

T2DM and AD are among the leading global causes of premature death and share common risk factors such as aging, insulin resistance, and metabolic dysfunction. Evidence shows that AD is nearly twice as prevalent in individuals with T2DM. Both diseases involve similar pathological mechanisms, including chronic inflammation, oxidative stress, and amyloid accumulation. Due to these overlaps, AD is often referred to as “type 3 diabetes.” Some antidiabetic drugs have also shown potential in slowing AD progression (Zu et al. 2021). AD is characterized by brain insulin resistance and insulin deficiency, while T2DM is characterized by peripheral insulin resistance (Moroz et al. 2008). AD and DM are widespread conditions with major global health impacts. Key enzymes such as AChE, BChE, α‐glucosidase, and α‐amylase are central to their treatment. Identifying natural inhibitors of these enzymes is crucial, as such compounds may offer safer alternatives to synthetic drugs and may help manage disease progression through both therapeutic use and dietary intake (İzol, Yılmaz, and Gülçin 2025).

Antioxidants are compounds that neutralize free radicals and reduce oxidative stress. They play a key role in preventing cellular damage and are involved in the treatment of numerous diseases (İzol, Turhan, et al. 2025). Oxidative stress plays a significant role in the development of various chronic diseases, including cardiovascular disease, DM, cancer, AD, inflammation, neurodegenerative disorders, pulmonary diseases, and hematological diseases. Reactive oxygen species (ROS), which are produced during normal metabolic processes in the body, can cause damage to biomolecules such as proteins, DNA, and membrane lipids (Liu et al. 2012; Nicolai et al. 2016). Beyond regulating ROS with antioxidants, targeting key enzymes involved in disease pathways is another effective therapeutic approach. For example, inhibiting amylase and glucosidase helps control diabetes, cholinesterase inhibition supports AD treatment, and blocking tyrosinase is beneficial for treating skin disorders (Llorent‐Martínez et al. 2019).

Such studies help to establish diagnostic characters for the plant material, ensuring consistency in pharmacognostic evaluation and preventing misidentification, which is particularly important for species with traditional uses and emerging pharmacological relevance. According to WHO guidelines, the morphological and anatomical characterization of medicinal plants is a fundamental step in the standardization of herbal materials (WHO 2011). Pharmacognosy is the science of natural drugs derived from plant, animal, or mineral sources. It involves the identification and characterization of raw materials through morphological and anatomical analysis, assessment of their purity and potency, and evaluation of their active constituents. It also covers aspects such as cultivation, harvesting, and preparation methods, as well as the physicochemical properties of bioactive compounds (Shinde et al. 2021). WHO outlines various quality control criteria for herbal materials. Identifying phytochemicals and standardizing plant materials are essential steps to ensure the safety and authenticity of herbal remedies. Pharmacognostic analysis, phytochemical screening, and physicochemical evaluations support the verification and reliable use of medicinal plants (Firoskhan and Muthuswamy 2021).

In this study, the pharmacognostic features of the aerial parts of *Ajuga chamaepitys* subsp. *chia* were collected and subjected to methanolic, aqueous, and essential oil extractions. Antioxidant (ABTS^•+^ and DPPH^•^), antidiabetic (α‐amylase and α‐glucosidase), and anticholinesterase (AChE and BChE) activities were evaluated using spectrophotometric assays. Total phenolic and tannin contents were determined by Folin–Ciocalteu methods. Essential oil composition was analyzed via GC–MS/MS. Morphological and anatomical features were examined microscopically. Additionally, molecular docking studies were conducted to predict the binding affinities of all volatile compounds to key metabolic and neurological enzymes. All experiments were performed in triplicate, and statistical analyses were conducted using the Kruskal–Wallis test.

## 2 Material and Method

### 2.1 Plant Material

The aerial parts of *A. chamaepitys* subsp. *chia* were collected from Karaman (Central Turkey) in July 2022 and identified by Dr. Ömer ÇEÇEN. Voucher specimens were deposited at the Herbarium of Biodiversity Application and Research Center, Karamanoglu Mehmetbey University (KMUB 7321).

### 2.2 Extraction

The methanolic and aqueous extracts of *A. chamaepitys* subsp. *chia* seeds were obtained from dried flowering aerial parts. The plant material was first finely ground and mixed with methanol using a mechanical mixer. The resulting mixture was then allowed to macerate at room temperature for 8 h and 3 days, respectively. After maceration, the extract was evaporated to dryness and weighed. To prepare the aqueous extract, 50 g of dried flowering aerial parts were boiled in water for 2 h. The resulting mixture was then filtered and frozen at −80°C. The frozen extract was subjected to lyophilization, and the weight was recorded.

### 2.3 Essential Oil Extraction and Analysis

The essential oil was obtained from the flowering aerial parts of *A. chamaepitys* by using the Clevenger apparatus, which involved adding water during the 3–4 h extraction process. The resulting oil was then subjected to gas chromatography–mass spectrometry (GC–MS) analysis, using an Innowax FSC column (60 m × 0.25 mm, 0.25 μm film thickness) and helium gas as the carrier (0.8 mL/min). The initial temperature of the GC oven was set at 60°C for 10 min and then increased at a rate of 4°C per minute to 220°C, where it was held for 10 min before being further increased to 240°C at a rate of 1°C per minute. The injector temperature was set at 250°C, and the mass spectra were recorded across a range of m/z 35 to 450 at 70 eV. The GC analysis was performed using an Agilent 6890 N GC system, with the flame ionization detector (FID) temperature set at 300°C. Identification of the essential oil components was carried out by comparison of their relative retention times with those of authentic samples or by comparison of their relative retention index (RRI) to a series of *n*‐alkanes. Computer matching against commercial (Wiley GC/MS Library, MassFinder Software 4.0) (McLafferty and Stauffer 1989; Hochmuth 2008) and in‐house “Başer Library of Essential Oil Constituents” built up by genuine compounds and components of known oils.

### 2.4 *α*‐Amylase Inhibition Assay

The *α*‐amylase inhibitory activity was assessed using a modified version of the method described by Nampoothiri et al. (2011) and further adapted by Yuca et al. (2024). Both the test samples and acarbose were dissolved in dimethyl sulfoxide (DMSO). A 100 μL aliquot of each sample was mixed with 100 μL of a 1% starch solution prepared in 20 mM sodium phosphate buffer (pH 6.9, containing 6 mM sodium chloride), followed by incubation at 25°C for 10 min. After the initial incubation, 100 μL of *α*‐amylase solution (0.5 mg/mL) was added to the reaction mixture, and incubation was continued under the same conditions for another 10 min. The enzymatic reaction was then terminated by adding the dinitrosalicylic acid (DNS) color reagent, after which the mixture was heated in a boiling water bath for 5 min. Following cooling to room temperature, the absorbance was recorded at 540 nm using a microplate reader. Acarbose was used as the reference inhibitor. All experiments were performed in triplicate. The percentage inhibition of α‐amylase was calculated using the following equation:

$$\text{Inhibition}\;\left(\%\right)=\left(1-\;{\mathrm{\Delta A}}_{54\text{0sample}}/{\mathrm{\Delta A}}_{54\text{0control}}\right)\times 100$$

### 2.5 *α*‐Glucosidase Inhibition Assay

The *α*‐glucosidase inhibitory activity was assessed using a modified version of the method of Bachhawat et al. (2011) described by Yuca et al. (2024). The assay was performed in a 96‐well microplate format. Initially, 50 μL of phosphate buffer (50 mM, pH 6.9) was added to each well, followed by the addition of 10 μL of *α*‐glucosidase enzyme (1 U/mL) and 20 μL of plant extract at concentrations ranging from 1 to 5000 μg/mL. The mixture was then incubated at 37°C for 5 min. After incubation, 20 μL of 3 mM *p*‐nitrophenyl‐*α*‐D‐glucopyranoside (pNPG) was introduced as a substrate, and the reaction was allowed to proceed at 37°C for 30 min. The reaction was subsequently terminated by adding 50 μL of 0.1 M sodium carbonate. All solutions were prepared in phosphate buffer. Acarbose was used as a positive control to compare the inhibitory activity. The formation of *p*‐nitrophenol (pNP) was quantified by measuring absorbance at 405 nm using a spectrophotometer. Each experiment was conducted in triplicate. The percentage inhibition was calculated using the following formula:

$$\text{Inhibition}\;\left(\%\right)=\left(1-{\mathrm{\Delta A}}_{40\text{5sample}}/{\mathrm{\Delta A}}_{40\text{5control}}\right)\times 100$$

### 2.6 Acetylcholinesterase (AChE) and Butyrylcholinesterase (BChE) Inhibition

The inhibitory activities against acetylcholinesterase (AChE) and butyrylcholinesterase (BChE) were assessed using a modified version of the method described by Ingkaninan et al. (2000), with slight adaptations as reported by Yuca et al. (2024). Both the test samples and the reference inhibitor, donepezil, were dissolved in DMSO. The assay was conducted in a 96‐well microplate, where a reaction mixture was prepared by combining 125 μL of 5,5′‐dithiobis‐(2‐nitrobenzoic acid) (DTNB, Ellman's reagent), 25 μL of substrate (acetylthiocholine for AChE or butyrylthiocholine for BChE), 50 μL of Tris–HCl buffer, and 25 μL of the test sample. This mixture was incubated under controlled conditions, after which 25 μL of either AChE or BChE enzyme solution was added to initiate the reaction. The extent of enzyme inhibition was quantified by measuring absorbance at 405 nm using a spectrophotometer. Donepezil was used as the standard reference inhibitor. All experiments were performed in triplicate. The percentage inhibition of enzyme activity was calculated using the following formula:

$$\text{Inhibition}\;\left(\%\right)=\left(1-{\mathrm{\Delta A}}_{40\text{5sample}}/{\mathrm{\Delta A}}_{40\text{5control}}\right)\times 100$$

### 2.7 ABTS ^·+^ Scavenging Activity

The ABTS cation radical scavenging activity was evaluated using the method described by Re et al. (1999), with minor modifications by Karakaya et al. (2023). α‐Tocopherol and Trolox were utilized as reference antioxidants, while a 2 mM ABTS^•+^ solution served as the free radical source. Stock solutions were prepared at concentrations ranging from 1 to 150 μg/mL for both the samples and the standards. The absorbance of all samples was recorded at 734 nm against a blank containing phosphate buffer. Each measurement was conducted in triplicate to ensure reproducibility and reliability. The ABTS^•+^ scavenging activity was determined using the following equation:

$$\%\text{Inhibition}=\left[\left(A_{\text{control}}-A_{\text{Sample}}\right)/A_{\text{control}}\right]\times 100$$

### 2.8 DPPH ^•^ Scavenging Activity

The DPPH radical scavenging activity was determined according to the method described by Blois (1958), with slight modifications (Karakaya et al. 2023). α‐Tocopherol and Trolox were used as reference antioxidants, while a 1 mM DPPH^•^ solution served as the free radical source. Stock solutions of both the samples and standards were prepared at concentrations ranging from 1 to 150 μg/mL. The absorbance of all samples was measured at 517 nm against a blank containing absolute ethanol. To ensure precision and reliability, all measurements were performed in triplicate. The DPPH^•^ scavenging activity was calculated using the following equation:

$$\%\text{Inhibition}=\left[\left(A_{\text{control}}-A_{\text{Sample}}\right)/A_{\text{control}}\right]\times 100$$

### 2.9 Total Phenolic Content

The total phenolic content of the samples was determined using a modified version of the Folin and Denis method, later refined by Slinkard and further adapted in our laboratory (Folin and Denis 1912; Slinkard and Singleton 1977). Gallic acid was used as the standard, and a stock solution at a concentration of 1 mg/mL was prepared accordingly. From this stock solution, a series of dilutions (100, 200, 300, 400, 500, 600, and 700 μg/mL) was prepared. Each dilution was then reacted with Folin–Ciocalteu reagent (FCR) and 2% Na₂CO₃. To construct a standard calibration curve, the absorbance of these gallic acid solutions was measured at 760 nm, with distilled water serving as the blank. Similarly, a 1 mg/mL stock solution of the samples was prepared, and their absorbance values were determined using the Folin–Ciocalteu method. The total phenolic content was calculated by substituting the absorbance values of the samples into the equation Absorbance = 0.0015 × Gallic acid − 0.0017, derived from the standard calibration curve at 760 nm. The results were expressed in terms of gallic acid equivalents (GAE). To ensure accuracy and reproducibility, all measurements were performed in triplicate.

### 2.10 Total Tannin Content

The total tannin content of the samples was determined following the methodology described by Makkar (Makkar and Makkar 2003), with slight modifications. Tannic acid was used as the standard, and a stock solution with a concentration of 1 mg/mL was prepared. From this stock solution, a series of dilutions ranging from 50 to 1000 μg/mL was prepared. Each dilution was then mixed with Folin–Ciocalteu reagent (FCR) and 2% Na₂CO₃. After an appropriate incubation period, the absorbance of the samples was measured at 725 nm against a blank containing distilled water. Similarly, a 1 mg/mL stock solution of the samples was prepared, and their absorbance values were determined using the Folin–Ciocalteu method. The total tannin content was calculated by substituting the absorbance values of the samples into the equation Absorbance = 0.0017 × Tannic acid − 0.0119, derived from the standard curve at 725 nm. The results were expressed in tannic acid equivalents (TAE). To ensure accuracy and reproducibility, all measurements were conducted in triplicate.

### 2.11 Morphological Study and Anatomical Studies

The Leica S8AP0 stereo microscope was employed for the identification and morphological examination of *A. chamaepitys* subsp. *chia*. A total of ten individuals from each species were analyzed to assess the structural characteristics of stems, leaves, flowers, and roots. Detailed morphological descriptions were provided, accompanied by scientific illustrations to highlight distinguishing features and facilitate accurate identification.

The plant material was initially preserved in 70% ethanol to maintain its structural integrity and prevent degradation. Following preservation, transverse sections of both stems and leaves were carefully prepared and examined in detail. The anatomical features were analyzed using Sartur's reagent and chloral hydrate solution to enhance tissue differentiation and facilitate microscopic observations.

### 2.12 Molecular Docking Studies

In this study, forty‐five distinct metabolites were identified in the volatile oil of *Ajuga chamaepitys* subsp. *chia*, and all were subjected to molecular docking analyses (representative renderings and complete results are provided in Supporting Information, Table S1). Nevertheless, we elected to concentrate our detailed evaluation on three principal constituents—α‐pinene, β‐pinene, and germacrene D—on the basis of their highest relative abundances in GC–MS profiling and their previously reported inhibitory activities against enzymes implicated in Alzheimer's disease and type 2 diabetes. The target enzymes included acetylcholinesterase (AChE, PDB ID: 4EY7), butyrylcholinesterase (BChE, PDB ID: 5DYW), α‐amylase (PDB ID: 2QV4), and α‐glucosidase (PDB ID: 3A4A). The 4EY7, 5DYW, and 2QV4 structures were obtained from the RCSB Protein Data Bank (https://www.rcsb.org/). Since the human α‐glucosidase structure has not yet been experimentally resolved, a homology model based on *Saccharomyces cerevisiae* isomaltase (PDB ID: 3A4A) was utilized, as previously described by Peytam et al. (2021) and Adib et al. (2018).

Protein structures were preprocessed using Discovery Studio Visualizer 2024 Client (BIOVIA, Dassault Systèmes, San Diego, CA, USA, Biovia 2021). This involved the removal of crystallographic water molecules and nonessential ligands. Polar hydrogen atoms and Gasteiger charges were added using AutoDock Tools v1.5.7. All ligand structures were retrieved from the PubChem database (https://pubchem.ncbi.nlm.nih.gov/) in SDF format. Energy minimization was performed using Discovery Studio Visualizer, and the structures were converted to PDBQT format.

Molecular docking simulations were conducted using AutoDock Vina v1.2.7, with the exhaustiveness parameter set to 8 (Trott and Olson 2010; Eberhardt et al. 2021). Grid boxes were configured to encompass the active sites of each target enzyme, based on literature‐reported catalytic residues and preliminary modeling analyses. The grid box was set over the active site of receptors with the following parameters: for acetylcholinesterase (AChE, PDB ID: 4EY7) (Júnior et al. 2025): center at *x* = −14.07, *y* = −43.93, *z* = 27.92, size as *x* = 40, *y* = 40, *z* = 40; for butyrylcholinesterase (BChE, PDB ID: 5DYW) (Shakil 2021): center at *x* = −5.33, *y* = −10.63, *z* = −12.73, size as *x* = 60, *y* = 60, *z* = 60; for α‐amylase (PDB ID: 2QV4) (Akshatha et al. 2021): center at *x* = 16.11, *y* = 49.57, *z* = 25.06, size as *x* = 25.76, *y* = 25.78, *z* = 26.13; and for α‐glucosidase (PDB ID: 3A4A) (Karrouchi et al. 2020) center at *x* = 22.625, *y* = −8.069, *z* = 24.158, size as *x* = 40, *y* = 40, *z* = 40. For each ligand–protein pair, the binding energies (kcal/mol) of the top‐ranked docking poses were recorded. The most favorable poses were selected for further analysis. Docked conformations were analyzed using Discovery Studio Visualizer to assess hydrogen bonds, hydrophobic interactions, and π‐interactions. These molecular insights were used to support and interpret the experimental enzyme inhibition results.

### 2.13 Statistical Analysis

All experiments were conducted in triplicate. Statistical significance was evaluated using the Kruskal–Wallis test.

## 3 Results

### 3.1 Extraction

The methanol extract yielded 10.24%, whereas the water extract demonstrated a significantly higher yield of 21.92%. This suggests that the water extraction method is more efficient in extracting compounds from the plant material compared to the methanol extract, highlighting the potential differences in the solubility and availability of active constituents in each solvent.

### 3.2 Essential Oil

The composition of the essential oil from *A. chamaepitys* (L.) Schreb. subsp. *chia* (Schreb.) Arcang is presented in Table 1. The essential oil, derived from the aerial parts with flowers, yielded 0.002% and appeared yellow in color. A total of 45 compounds were identified, which accounted for 92.5% of the oil. The major component was β‐pinene, which constituted 19.8%, followed by α‐pinene at 12.8% and germacrene D at 10.0%. The largest group of compounds was monoterpene hydrocarbons, making up 39.8%, with sesquiterpene hydrocarbons representing 23.5%.

**TABLE 1:** The composition of the essential oil of *Ajuga chamaepitys* subsp. *chia*.

No	RRI	Compound	%	Identification method (IM)
1	1032	*α*‐Pinene	12.8	*t*_R_, MS
2	1035	*α*‐Thujene	0.4	*t*_R_, MS
3	1076	Camphene	2.5	*t*_R_, MS
4	1118	*β*‐Pinene	19.8	*t*_R_, MS
5	1132	Sabinene	< 0.1	*t*_R_, MS
6	1174	Myrcene	1.0	*t*_R_, MS
7	1176	*α*‐Phellandrene	0.2	*t*_R_, MS
8	1203	Limonene	2.4	*t*_R_, MS
9	1213	1,8‐Cineol	3.1	*t*_R_, MS
10	1280	*p*‐Cymene	0.7	*t*_R_, MS
11	1466	*α‐*Cubebene	< 0.1	MS
12	1497	*α‐*Copaene	1.6	MS
13	1532	Camphor	3.1	*t*_R_, MS
14	1535	*β‐*Bourbonene	< 0.1	MS
15	1553	Linalool	< 0.1	*t*_R_, MS
16	1586	Pinocarvone	0.1	*t*_R_, MS
17	1590	Bornyl acetate	0.9	*t*_R_, MS
18	1597	*β‐*Copaene	2.1	MS
19	1611	Terpinen‐4‐ol	0.1	*t*_R_, MS
20	1612	*β‐*Caryophyllene	2.6	*t*_R_, MS
21	1648	Myrtenal	0.7	MS
22	1662	Pulegone	0.7	*t*_R_, MS
23	1687	*α‐*Humulene	0.6	*t*_R_, MS
24	1704	*γ‐*Muurolene	3.0	MS
25	1706	*α‐*Terpineol	1.6	*t*_R_, MS
26	1719	Borneol	0.2	*t*_R_, MS
27	1726	Germacrene D	10.0	MS
28	1740	*α‐*Muurolene	< 0.1	MS
29	1751	Carvone	0.5	*t*_R_, MS
30	1755	Bicyclogermacrene	1.9	MS
31	1773	*δ‐*Cadinene	1.1	MS
32	1804	Myrtenol	0.1	MS
33	1849	Calamelene	0.6	MS
34	1900	*epi*‐Cubebol	0.4	MS
35	2008	Caryophyllene oxide	3.3	*t*_R_, MS
36	2057	Ledol	0.5	MS
37	2069	Germacren‐D‐4‐ol	3.2	MS
38	2104	Viridiflorol	3.5	MS
39	2131	Hexahydrofarnesyl acetone	0.9	MS
40	2144	Spathulenol	0.4	MS
41	2187	T‐Cadinol	2.4	MS
42	2219	‐Cadinol (=*Torreyol*)	1.1	MS
43	2255	*α‐*Cadinol	0.9	MS
44	2300	Tricosane	1.5	*t*_R_, MS
45	2622	Phytol	< 0.1	MS
		Monoterpene hydrocarbons	39.8	
		Oxygenated monoterpenes	11.1	
		Sesquiterpene hydrocarbons	23.5	
		Oxygenated sesquiterpenes	15.7	
		Diterpenes	< 0.1	
		Others	2.4	
		Total	92.5	

*Note:* RRI: Relative retention indices calculated against *n*‐alkanes; %: calculated from FID data; IM: Identification method *t*_R_, identification based on the retention times of genuine compounds on the HP Innowax column; MS, identified on the basis of computer matching of the mass spectra with those of the Wiley and MassFinder libraries and comparison with literature data.

### 3.3 Enzyme Inhibition Assays

Table 2 presents the in vitro antidiabetic and anticholinesterase activities of *A. chamaepitys* subsp. *chia* extracts. In the antidiabetic assays, neither the methanolic (MeOH) nor the aqueous extract exhibited *α*‐glucosidase inhibition. However, both extracts demonstrated *α*‐amylase inhibitory activity, with the methanolic extract showing a moderate inhibition of 28.36% at 5000 μg/mL, while the aqueous extract exhibited a lower inhibition of 20.20%. In comparison, the standard antidiabetic drug, acarbose, displayed significantly higher inhibition, with 18.80% inhibition of *α*‐glucosidase at 500 μg/mL and 69.08% inhibition of *α*‐amylase at 5000 μg/mL.

**TABLE 2:** In vitro antidiabetic and anticholinesterase activities of extracts from *Ajuga chamaepitys* subsp. *chia*.

Samples	Antidiabetic activities		Anticholinesterase activities	
	*α*‐Glucosidase inhibition (%) (500 μg/mL) (mean ± std)	*α*‐Amylase inhibition (%) (5000 μg/mL) (mean ± std)	Acetylcholinesterase inhibition (%) (100 μg/mL) (mean ± std)	Butyrylcholinesterase inhibition (%) (1000 μg/mL) (mean ± std)
MeOH extract	N.D.	28.36 ± 2.50	21.85 ± 1.28	12.92 ± 1.31
Aqueous extract	N.D.	20.20 ± 6.51	22.82 ± 3.77	6.83 ± 4.75
Acarbose (positive control)	18.80 ± 1.41	69.08 ± 1.47	—	—
Donepezil (positive control)	—	—	100 ± 1.14	100 ± 1.30

*Note:* Results are expressed as mean ± standard deviation (SD) of three independent replicates. Statistical significance was evaluated using the Kruskal–Wallis test.

Abbreviation: N.D., not determined.

Regarding anticholinesterase activity, the methanolic extract exhibited moderate inhibition of acetylcholinesterase (AChE) at 21.85% and butyrylcholinesterase (BChE) at 12.92% at 100 μg/mL and 1000 μg/mL, respectively. The aqueous extract displayed a similar level of AChE inhibition (22.82%) but a lower BChE inhibition (6.83%). In contrast, the reference inhibitor, donepezil, showed complete inhibition of both enzymes, with 100% for AChE and BChE.

### 3.4 Antioxidant Activity and Total Phenolic/Tannin Compound Test Results

The antioxidant activity of the samples was evaluated using ABTS^•+^ and DPPH^•^ scavenging assays at a concentration of 50 μg/mL. The results indicate significant differences in the free radical scavenging capacities among the tested samples. Trolox exhibited the highest antioxidant activity, with 52.509 ± 0.0095% inhibition in the ABTS^•+^ assay and 91.309 ± 0.0042% inhibition in the DPPH^•^ assay, demonstrating its strong radical scavenging potential. α‐Tocopherol showed moderate activity, with 23.284 ± 0.0258% inhibition in the ABTS^•+^ assay and 59.159 ± 0.0112% inhibition in the DPPH^•^ assay (Table 3).

**TABLE 3:** ABTS^•+^ and DPPH^•^ scavenging activity test results.

Antioxidant activity assays		
Samples	ABTS^•+^ scavenging activity (% inhibition of 50 μg/mL ± SD)	DPPH^•^ scavenging activity (% inhibition of 50 μg/mL ± SD)
*α‐*Tocopherol	23.284 ± 0.0258	59.159 ± 0.0112
Trolox	52.509 ± 0.0095	91.309 ± 0.0042
MeOH Extract	23.013 ± 0.0159	8.114 ± 0.0452
Aqueous Extract	21.707 ± 0.0059	4.614 ± 0.0050

*Note:* Results are expressed as mean ± standard deviation (SD) of three independent replicates. Statistical analysis was performed using the Kruskal–Wallis test.

Abbreviation: SD, standard deviation.

Among the *A. chamaepitys* subsp. *chia* extracts, the methanolic extract displayed slightly higher scavenging activity than the aqueous extract. The methanolic extract exhibited 23.013 ± 0.0159% inhibition in the ABTS^•+^ assay and 8.114 ± 0.0452% inhibition in the DPPH^•^ assay, while the aqueous extract showed 21.707 ± 0.0059% inhibition in the ABTS^•+^ assay and only 4.614 ± 0.0050% inhibition in the DPPH^•^ assay. These results suggest that the methanolic extract has a slightly better antioxidant capacity compared to the aqueous extract, although both extracts are considerably less effective than Trolox and α‐Tocopherol (Table 3).

Overall, Trolox demonstrated the highest antioxidant activity, followed by α‐Tocopherol, while *A. chamaepitys* subsp. *chia* extracts exhibited relatively low free radical scavenging potential, particularly in the DPPH^•^ assay.

The total phenolic and tannin contents of the *A. chamaepitys* subsp. *chia* extracts were determined and expressed as micrograms of GAE per milligram of extract for phenolics and micrograms of TAE per milligram of extract for tannins. The methanolic extract exhibited a higher total phenolic content (14.261 ± 0.0005 μg GAE/mg extract) compared to the aqueous extract (9.904 ± 0.0007 μg GAE/mg extract). Similarly, the total tannin content was also higher in the methanolic extract (34.444 ± 0.0005 μg TAE/mg extract) than in the aqueous extract (30.377 ± 0.0007 μg TAE/mg extract) (Table 4).

**TABLE 4:** Total phenolic/tannin compound test results.

Samples	Total phenolic compound (μg GAE/mg extract ± SD)	Total tannin compound (μg TAE/mg extract ± SD)
MeOH extract	14.261 ± 0.0005	34.444 ± 0.0005
Aqueous extract	9.904 ± 0.0007	30.377 ± 0.0007

*Note:* Results are expressed as mean ± standard deviation (SD) of three independent replicates. Statistical comparisons were performed using the Kruskal–Wallis test to evaluate significance.

Abbreviation: SD, standard deviation.

These results suggest that methanol is a more effective solvent than water for extracting phenolic and tannin compounds from *A. chamaepitys* subsp. *chia*. The higher content of these bioactive compounds in the methanolic extract may contribute to its relatively better antioxidant activity observed in previous assays.

### 3.5 Morphological Study and Anatomical Studies

**FIGURE 1:**
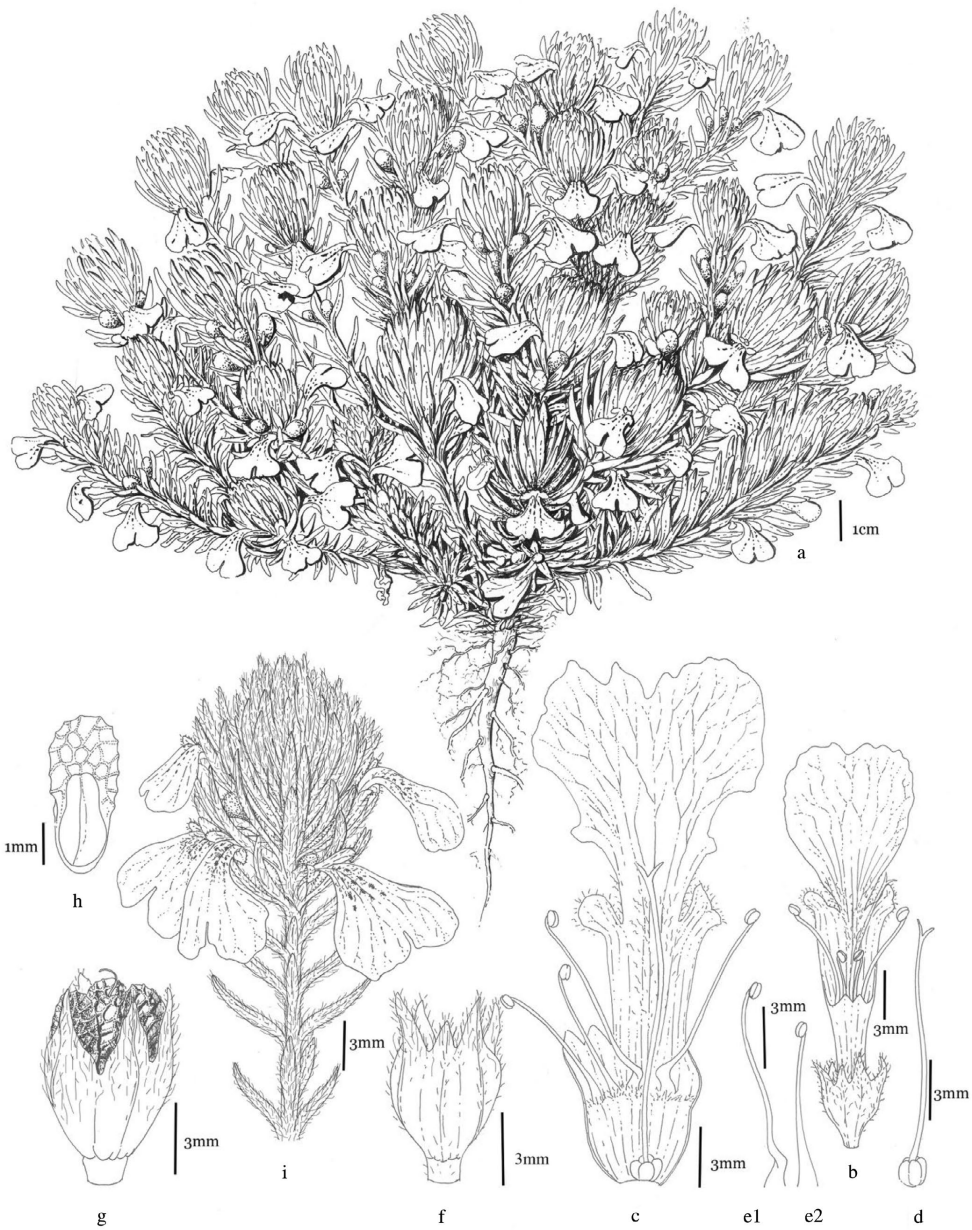
General appearance of *Ajuga chamaepitys* (a), flower (b), flower longitudinal section (c), pistil (d), stamens (e1, e2), calyx (f), seeds in calyx (g), seed (h), a branch with flowers and leaves (i).

Perennial herbs with prostrate or ascending, hairy stems. Cauline leaves are tripartite, cuneate to cuneate‐oblong, and hirsute, with floral leaves exhibiting a middle lobe significantly longer than broad. Bracts resemble cauline leaves. Verticillasters typically 2(−4) flowered. Calyx 4–7 mm, hairy. The corolla is yellow, 20–25 mm long, with a short tube, a bifid upper lip that is emarginate, and a much longer lower lip; the lower lip hairy, featuring two lateral lobes and a bilobed middle lobe. Stamens are four in number and usually exserted beyond the reduced upper lip. Nutlet between 2.5 and 4 mm, exhibiting a transversely rugose texture (Figure 1).

### 3.6 Leaf

**FIGURE 2:**
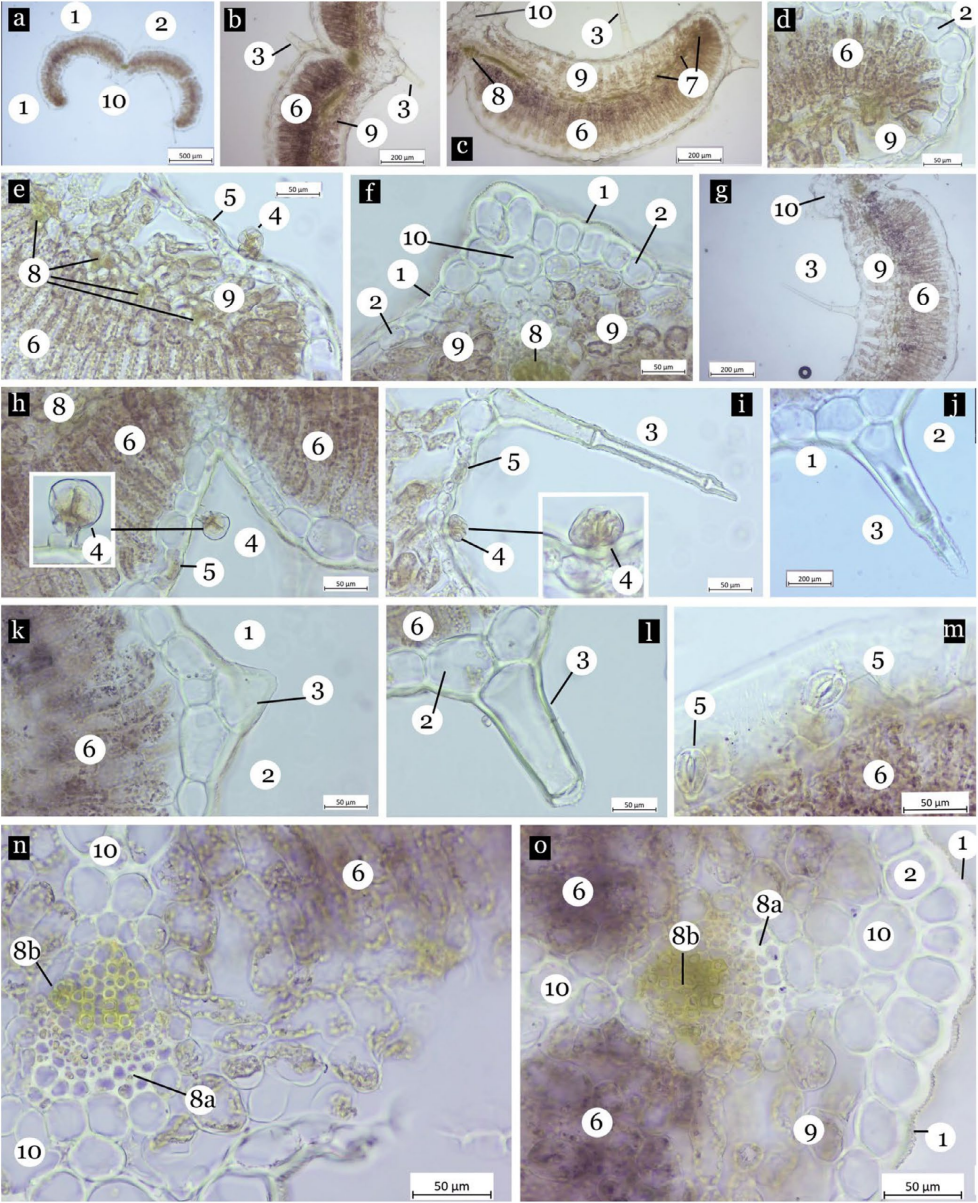
Leaf transverse‐section of *Ajuga chamaepitys* (a–o). Cuticle (1), epidermis (2), simple trichome (3), glandular trichome (4), stoma (5), palisade parenchyma (6), lateral vascular bundle (7), central vascular bundle (8), phloem (8a), xylem (8b), spongy parenchyma (9), collenchyma (10).

In the transverse section, the leaf lamina is covered by a cuticle layer on both sides of the leaf (Figure 2a,d,f,j–l,o). The epidermis is composed of a single layer of elliptical to rectangular cells, with those on the upper surface being larger. The nonglandular and glandular trichomes are present on both sides of the leaves (Figure 2b,c,e,g,h–l). The nonglandular trichome consists of one to four cells (Figure 2b,c,g,i–k). The glandular trichome is smaller and has three cells, including a neck cell in the stalk with two‐celled heads (Figure 2e,h,i). The midrib contains a large vascular bundle, surrounded by three to four layers of circular collenchyma cells on the adaxial side and two to three layers on the abaxial side (Figure 2a,c,f,g,n,o). The stomata are diacytic and predominantly located on the lower epidermis (Figure 2e,h,i,m). The leaf exhibits an amphistomatic and bifacial structure (Figure 2a–i,k–o). The palisade parenchyma consists of rectangular, elongated cells arranged in three to five layers, while the spongy parenchyma comprises two to four layers with intercellular spaces (Figure 2a–i,k,n,o). The vascular bundle has a collateral arrangement (Figure 2n,o).

### 3.7 Stem

**FIGURE 3:**
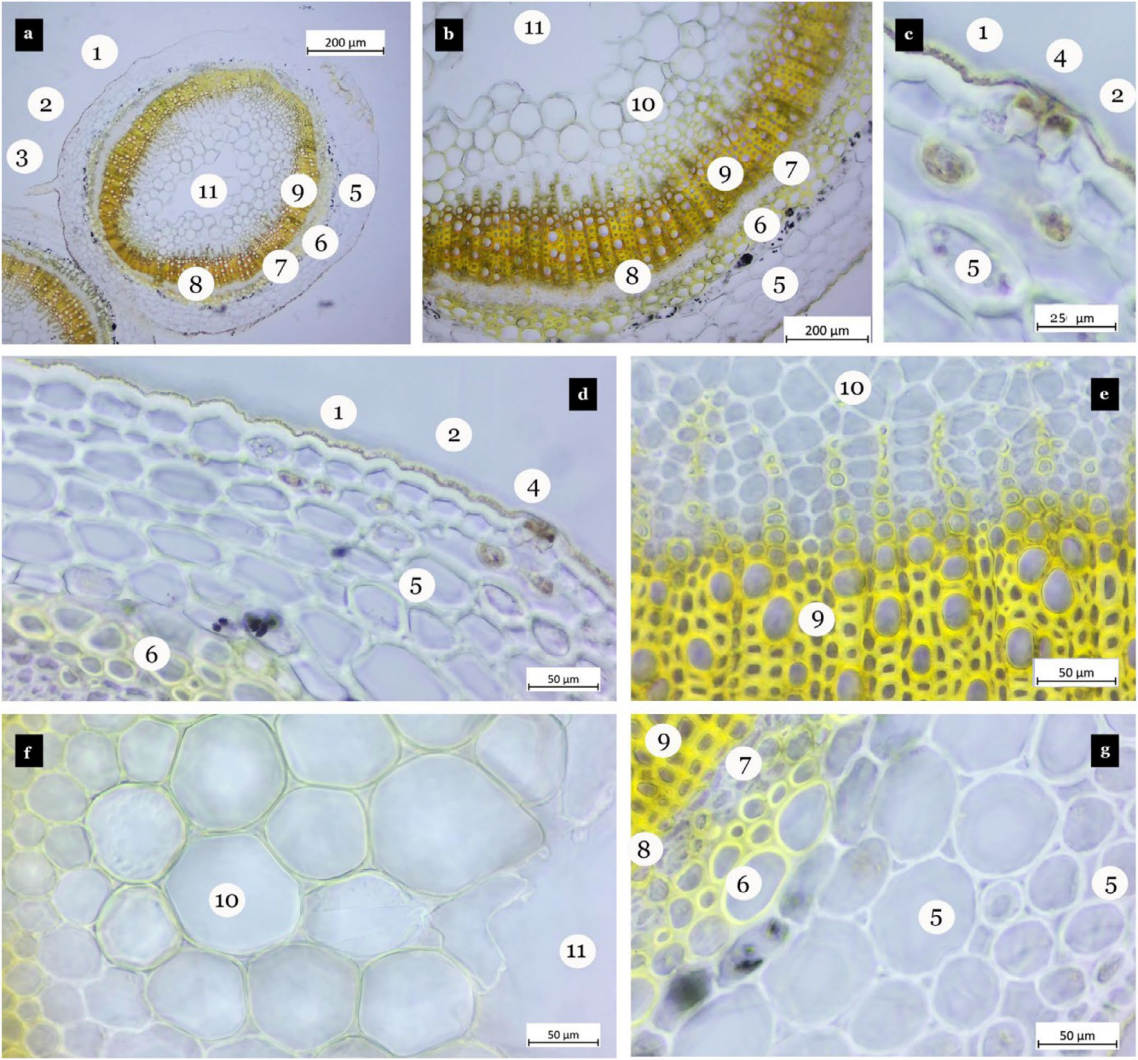
Stem cross‐section *Ajuga chamaepitys* (a–o). Cuticle (1), epidermis (2), simple trichome (3), glandular trichome (4), stoma (5), palisade parenchyma (6), lateral vascular bundle (7), central vascular bundle (8), phloem (8a), xylem (8b), spongy parenchyma (9), collenchyma (10).

The transverse section of the stem exhibits a circular to oblong shape (Figure 3a). The epidermis is coated with a cuticle layer and consists of a single layer of elliptical to rectangular cells (Figure 3d). The collenchyma tissue is organized in three to four layers (Figure 3d), while the cortex contains three to five layers of parenchyma. Starch granules are observed within the parenchymatic cells (Figure 3a,b,d,g). Sclerenchyma cell clusters are positioned above the phloem within the cortex (Figure 3b,d,g). The cambium is clearly distinguishable (Figure 3a,b). The pith is composed of both lignified and unlignified parenchymatic cells, which are large and exhibit polygonal or orbicular shapes (Figure 3a,b,e,f).

### 3.8 Molecular Docking Studies

Docking simulations of 45 volatile metabolites from *Ajuga chamaepitys* (L.) subsp. *chia* were carried out against four disease‐relevant enzymes—acetylcholinesterase (AChE, PDB ID: 4EY7), butyrylcholinesterase (BChE, PDB ID: 5DYW), α‐amylase (PDB ID: 2QV4), and α‐glucosidase (PDB ID: 3A4A)—using AutoDock Vina, with interaction analyses performed in Discovery Studio Visualizer. Donepezil and acarbose served as reference inhibitors for the cholinesterases and carbohydrate‐hydrolyzing enzymes, respectively. In the main text, we focus on α‐pinene, β‐pinene, and germacrene D—selected for their high relative abundance and consistently strong multitarget affinities—while the complete dataset remains available in the other figures.

Across all 45 metabolites, binding energies ranged from −10.6 to −5.9 kcal·mol^−1^ (mean −8.14 ±) for AChE; −8.5 to −5.7 kcal·mol^−1^ (mean −7.08) for BChE; −8.7 to −5.2 kcal·mol^−1^ (mean −6.62) for α‐amylase; and −8.6 to −5.0 kcal·mol^−1^ (mean −6.88) for α‐glucosidase (Table 5). Binding energy calculations revealed that germacrene D exhibited the lowest binding energies across all targets (Table 5), while detailed 2D interaction analyses (Table 6) visually confirmed the binding modes of each ligand within the active sites.

**TABLE 5:** Comparative binding energy (kcal/mol).

Compound	AChE (4EY7)	BChE (5DYW)	α‐Amylase (2QV4)	α‐Glucosidase (3A4A)
*α*‐Pinene	−5.6	−5.2	−6.1	−5.8
*α*‐Thujene	−5.6	−6.4	−7.1	−6.0
Camphene	−5.6	−5.9	−6.5	−5.7
*β*‐Pinene	−5.5	−5.0	−5.9	−5.7
Sabinene	−5.6	−6.6	−7.2	−5.7
Myrcene	−5.8	−6.3	−7.4	−6.3
*α*‐Phellandrene	−5.9	−6.5	−7.1	−6.3
Limonene	−5.9	−6.5	−7.2	−6.1
1,8‐Cineol	−5.5	−5.6	−6.9	−5.9
*p*‐Cymene	−5.6	−6.5	−7.5	−6.7
*α‐*Cubebene	−7.4	−7.7	−9.2	−8.1
*α‐*Copaene	−7.2	−7.2	−9.2	−7.8
Camphor	−5.7	−6.1	−6.9	−6.1
*β‐*Bourbonene	−7.3	−7.7	−8.7	−7.6
Linalool	−5.7	−6.9	−7.5	−6.3
Pinocarvone	−6.0	−6.1	−7.2	−6.1
Bornyl acetate	−6.8	−6.6	−8.0	−6.8
*β‐*Copaene	−7.8	−7.6	−9.0	−7.6
Terpinen‐4‐ol	−6.0	−6.8	−7.5	−6.2
*β‐*Caryophyllene	−7.8	−7.2	−8.7	−7.9
Myrtenal	−5.6	−6.0	−7.2	−6.2
Pulegone	−5.9	−6.3	−7.3	−6.8
*α‐*Humulene	−7.1	−7.1	−8.4	−8.1
*γ‐*Muurolene	−7.0	−7.5	−8.8	−8.1
*α‐*Terpineol	−6.3	−6.9	−7.4	−6.7
Borneol	−6.0	−5.9	−6.8	−5.9
Germacrene D	−7.1	−7.0	−9.1	−8.3
*α‐*Muurolene	−7.3	−7.5	−8.8	−8.0
Carvone	−5.9	−6.6	−7.3	−6.4
Bicyclogermacrene	−7.6	−7.0	−8.3	−7.7
*δ‐*Cadinene	−7.0	−7.3	−8.9	−8.2
Myrtenol	−6.2	−6.0	−7.2	−6.1
Calamelene	−7.2	−7.5	−8.5	−8.4
*epi*‐Cubebol	−7.4	−7.8	−9.9	−8.1
Caryophyllene oxide	−7.1	−7.7	−10.0	−7.9
Ledol	−7.5	−7.6	−8.6	−7.8
Germacren‐D‐4‐ol	−7.0	−7.6	−8.6	−8.1
Viridiflorol	−7.5	−7.3	−7.9	−7.7
Hexahydrofarnesyl acetone	−7.6	−8.6	−10.0	−6.8
Spathulenol	−7.3	−7.2	−8.1	−8.3
T‐Cadinol	−7.5	−7.7	−8.7	−8.5
‐Cadinol (=*Torreyol*)	−7.1	−7.5	−8.9	−8.3
*α‐*Cadinol	−7.5	−7.9	−9.6	−8.3
Tricosane	−5.2	−6.3	−10.6	−6.3
Phytol	−8.7	−7.5	−10.6	−7.1
Donepezil	−11.8	−9.9	—	—
Acarbose	—	—	−7.1	−7.2

*Note:* Comparative binding energies (in kcal/mol) of natural compounds and standards with target enzymes. Negative values indicate stronger binding affinity.

**TABLE 6:** Interaction types and key residues in ligand–enzyme complexes.

Ligand	Protein (PDB)	Hydrogen bonds	vdW Int.	Alkyl	π‐Alkyl	π‐Sigma	Residues
α‐Pinene	2QV4	0	6	2	4	0	HIS101, LEU162, LEU165, TRP58, TYR62
α‐Pinene	3A4A	0	7	2	4	1	HIS423, ILE419, PHE314
α‐Pinene	4EY7	0	12	0	9	3	HIS447, PHE338, TRP86, TRY337, TYR124, TYR337
α‐Pinene	5DYW	0	10	3	6	1	ALA328, PHE329, TRP430, TRP82, TYR332
β‐Pinene	2QV4	0	3	0	2	1	HIS299, TRP58, TYR62
β‐Pinene	3A4A	0	5	1	3	1	HIS423, ILE419, PHE314
β‐Pinene	4EY7	0	9	0	6	3	PHE338, TRP86, TRY337, TYR124, TYR337
β‐Pinene	5DYW	0	7	2	5	0	ALA328, HIS438, PHE329, TRP82, TYR332
Germacrene D	2QV4	2	1	0	1	0	TRP59
Germacrene D	3A4A	2	1	0	0	1	TYR158
Germacrene D	4EY7	1	3	0	1	2	PHE338, TYR337
Germacrene D	5DYW	1	3	1	0	0	ALA328, TRP82

*Note:* Hydrogen bonds = number of hydrogen bonds; vdW Int. = van der Waals interactions; Alkyl, π‐Alkyl, π‐Sigma = types of hydrophobic interactions; Residues = example amino acids involved.

Binding energy calculations demonstrated that germacrene D consistently displayed the lowest affinities across all targets. Specifically, germacrene D achieved binding energies of −9.1 and −8.3 kcal/mol for AChE and BChE, respectively, corresponding to approximately 77% and 84% of donepezil's reference scores (−11.8 and −9.9 kcal/mol) (PMC). Similarly, germacrene D exhibited binding scores of −7.1 and −7.0 kcal/mol for α‐amylase and α‐glucosidase, accounting for 89% and 83% of acarbose's binding energies (−7.1 and −7.2 kcal/mol) (PubMed). In contrast, neither α‐pinene nor β‐pinene surpassed the −6.1 kcal/mol threshold for any of the four enzymes, indicating a markedly weaker inhibitory potential relative to the standard inhibitors (Table 5).

**FIGURE 4:**
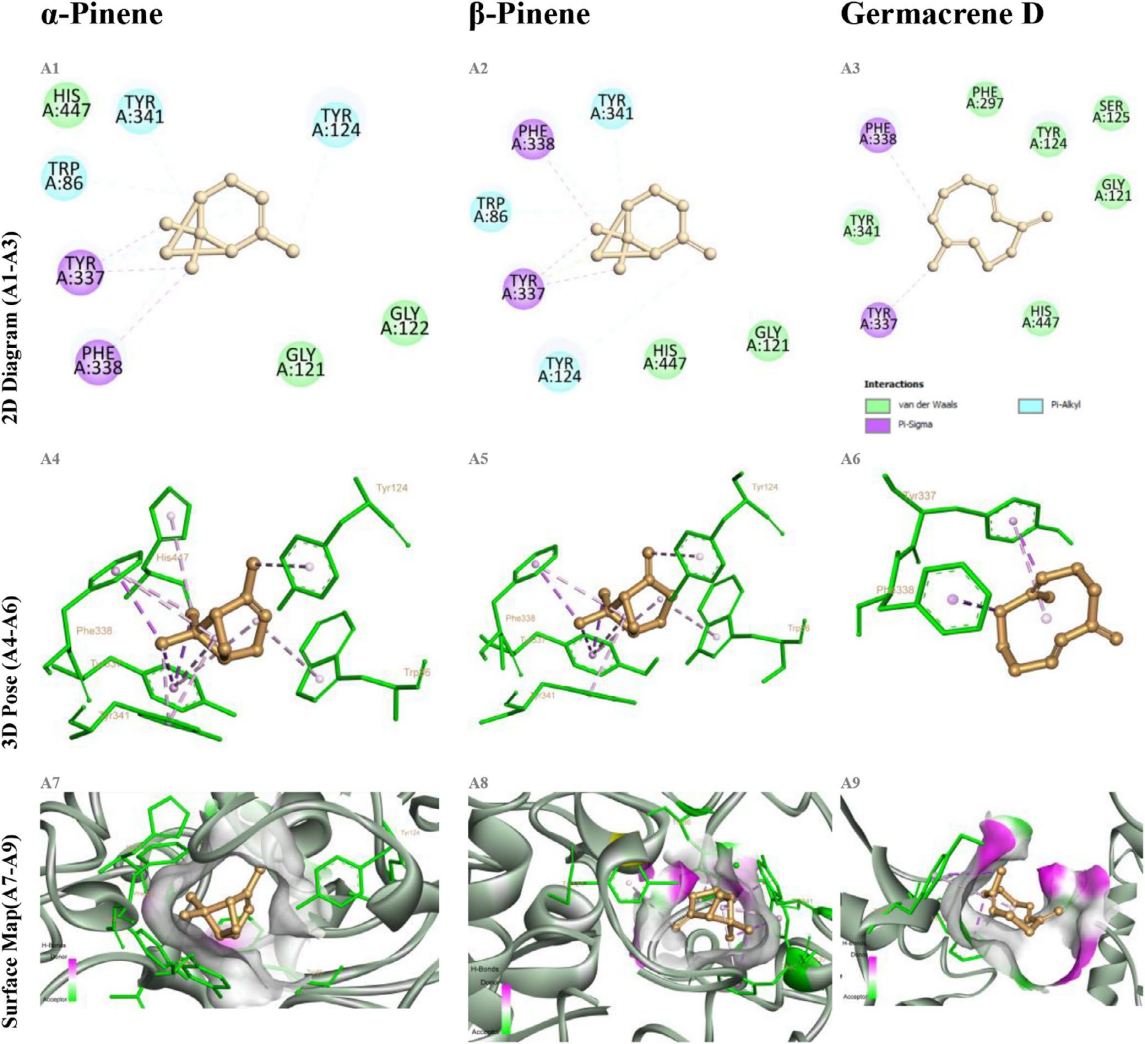
Docking of α‐pinene, β‐pinene, and germacrene D with acetylcholinesterase (AChE, PDB ID 4EY7). (A1–A3) Show the Discovery Studio 2D interaction diagrams indicating van der Waals (green), alkyl (cyan), and π‐alkyl (purple) contacts. (A4–A6) Present the 3D binding poses colored by residue type, with dotted lines for hydrophobic (purple) and π–π (lilac) interactions. (A7–A9) Display surface‐mapped views highlighting hydrogen‐bond donors in magenta, acceptors in green, and hydrophobic regions on a gray–white surface.

The AChE–α‐pinene complex exhibited a binding energy of −6.1 kcal/mol, forming π–π stacking and alkyl interactions with TRP86, PHE295, and TYR337. β‐Pinene established a similar aromatic interaction network but with a slightly lower binding energy of −5.9 kcal/mol. Germacrene D, demonstrating a binding energy of −6.5 kcal/mol, engaged in π–π and alkyl interactions with TRP86, PHE338, HIS447, and TYR337, while forming a hydrogen bond with GLU202 at a distance of 3.2 Å (Figure 4). This hydrogen bond network closely resembles the interactions established by donepezil with critical residues, thus reinforcing the potential of germacrene D as an AChE inhibitor.

**FIGURE 5:**
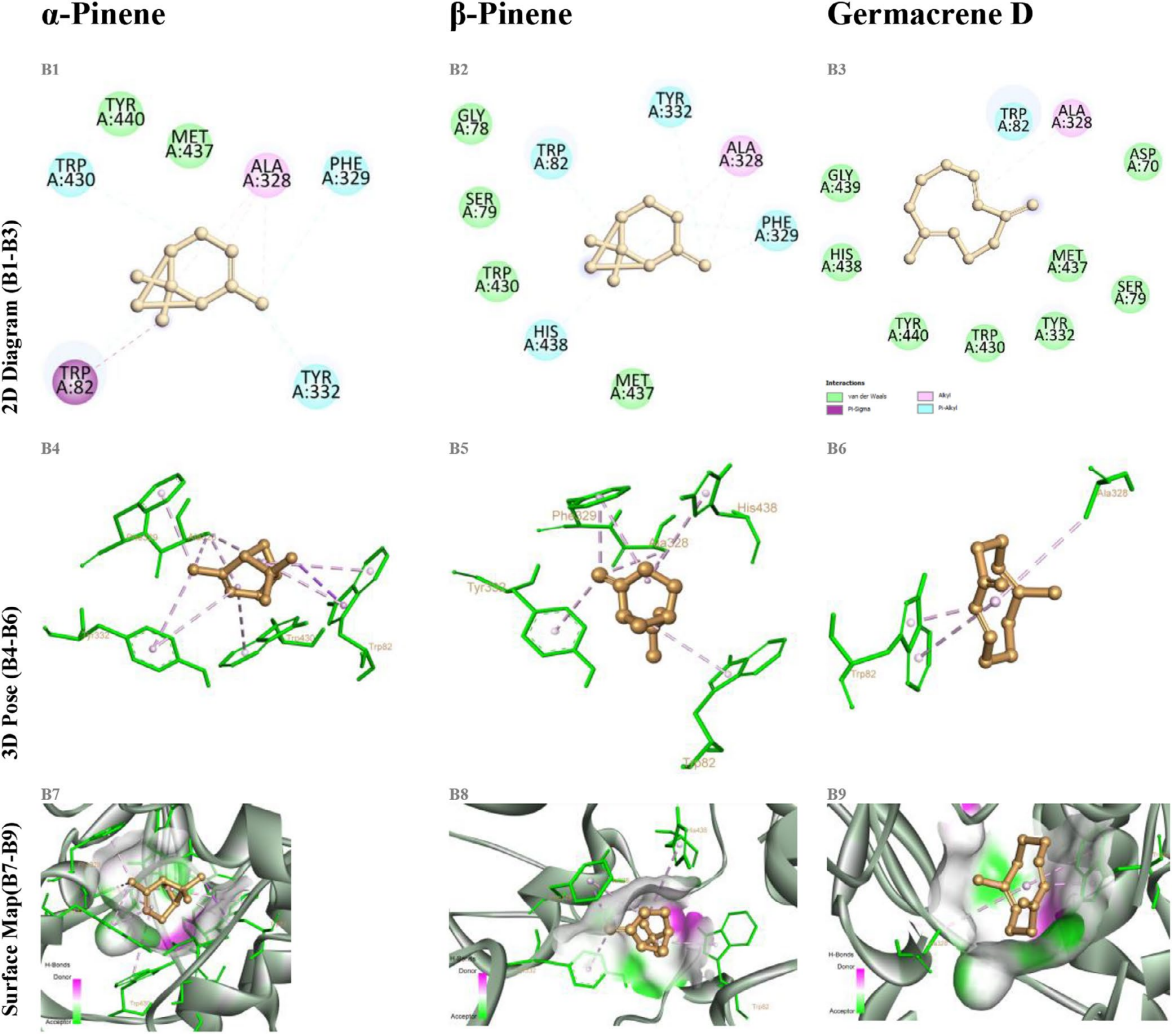
Docking of α‐pinene, β‐pinene, and germacrene D with butyrylcholinesterase (BChE, PDB ID 5DYW). Panels B1–B9 arranged as in Figure 4.

In the BChE docking studies, α‐pinene exhibited a binding energy of −5.8 kcal/mol through hydrophobic interactions involving TRP82, TYR332, and ALA328, while β‐pinene demonstrated π‐alkyl interactions with TRP82 and GLY116 at −5.7 kcal/mol. Germacrene D, with a binding energy of −6.2 kcal/mol, formed π‐alkyl and alkyl interactions with TRP82, TYR332, ALA328, and GLY116, in addition to establishing a hydrogen bond with SER198 at a distance of 3.5 Å (Figure 5). Notably, the hydrogen bond formed with SER198 enhanced the ligand's accommodation within the BChE active site, closely mimicking the binding mode of donepezil.

**FIGURE 6:**
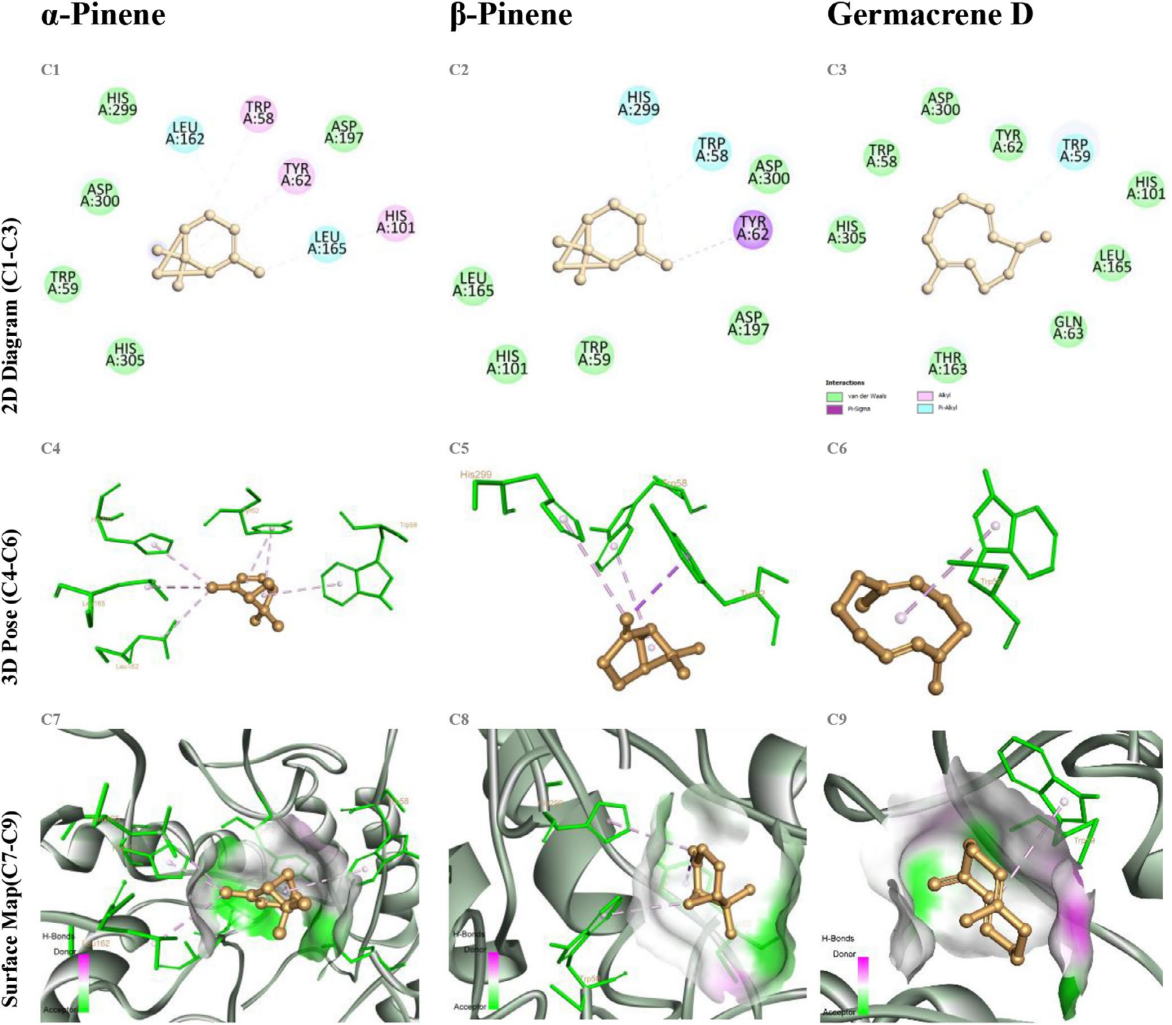
Docking of α‐pinene, β‐pinene, and germacrene D with α‐amylase (PDB ID 2QV4). Panels C1–C9 arranged as in Figure 4.

In the α‐amylase docking studies, α‐pinene bound with an energy of −5.6 kcal/mol, forming monofunctional interactions with GLU233, ASP197, and HIS299. Similarly, β‐pinene exhibited a binding energy of −5.5 kcal/mol, engaging in predominantly hydrophobic contacts with ASP300, HIS305, and TRP59. Germacrene D demonstrated a superior binding profile with an energy of −6.3 kcal/mol, forming two hydrogen bonds with ASP197 and GLU233 (2.8–3.1 Å), along with π‐alkyl interactions involving TRP59 and TYR62 (Figure 6). The resemblance of these hydrogen bonding patterns to those observed in acarbose–α‐amylase complexes underscores the biological relevance of germacrene D's binding strategy.

**FIGURE 7:**
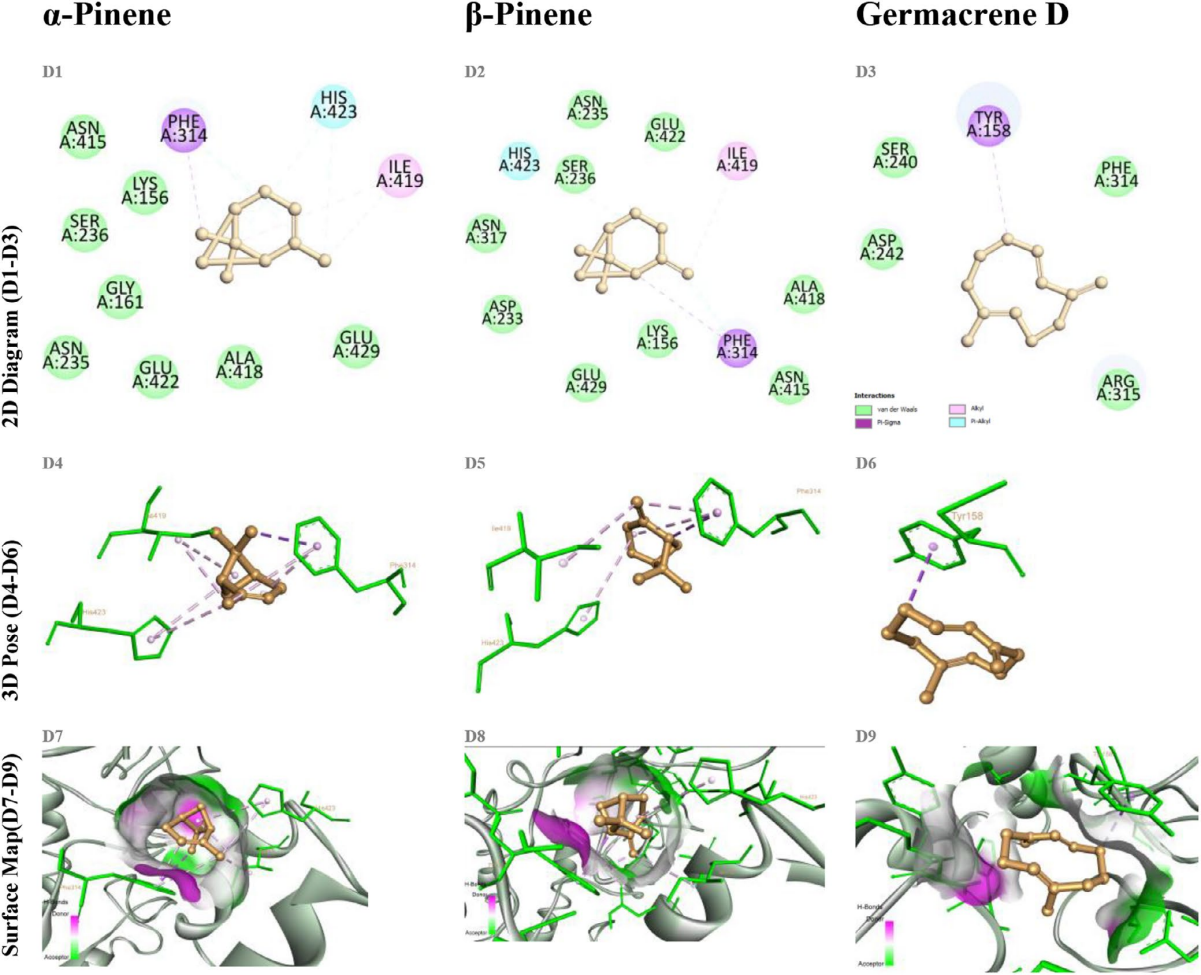
Docking of α‐pinene, β‐pinene, and germacrene D with α‐glucosidase (homology model PDB ID 3A4A). Panels D1–D9 arranged as in Figure 4.

For α‐glucosidase, α‐pinene and β‐pinene achieved binding energies of −5.2 and −5.0 kcal/mol, respectively, engaging only in superficial hydrophobic contacts, with a notable lack of specific aromatic interactions. In contrast, germacrene D attained a binding energy of −6.0 kcal/mol, forming two hydrogen bonds with ASP404 and ARG526 (2.7–3.3 Å) and π‐alkyl interactions with TYR158, thus demonstrating a more robust and specific interaction profile (Figure 7). The hydrogen bonding pattern observed for germacrene D closely mirrors that of acarbose, further highlighting its inhibitory potential against α‐glucosidase.

**FIGURE 8:**
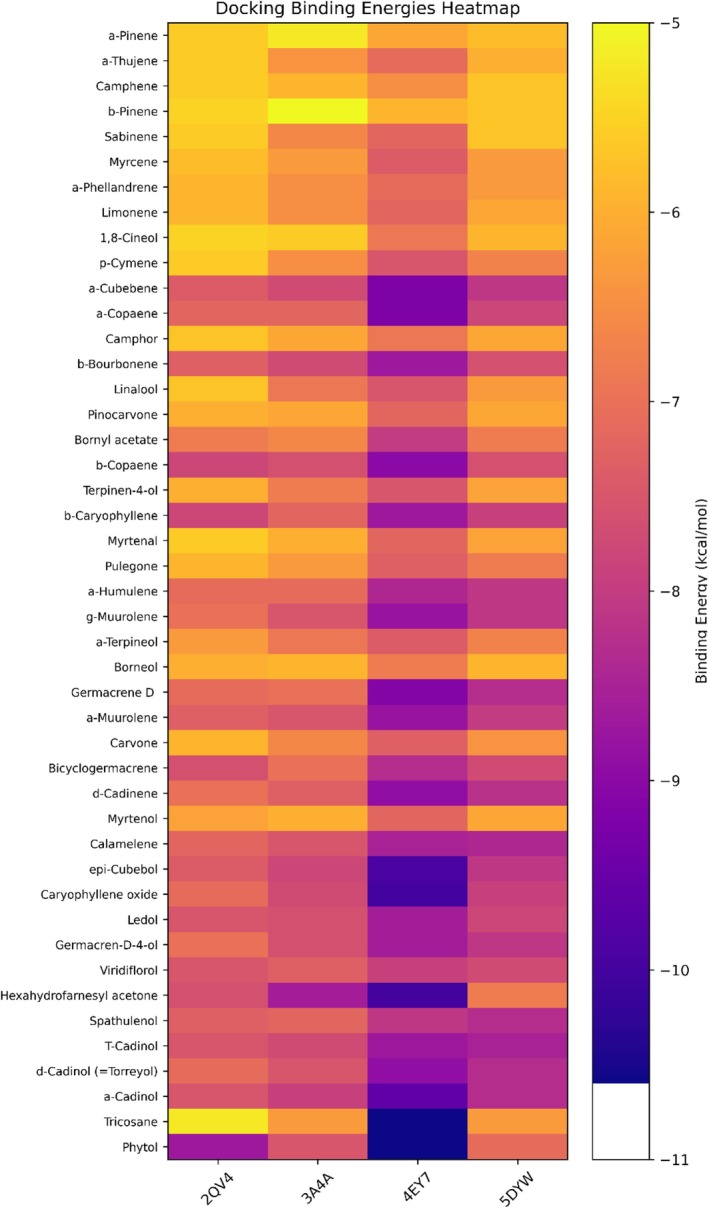
Molecular docking‐based heatmap representing the interaction energies of essential oil compounds with target enzymes.

**FIGURE 9:**
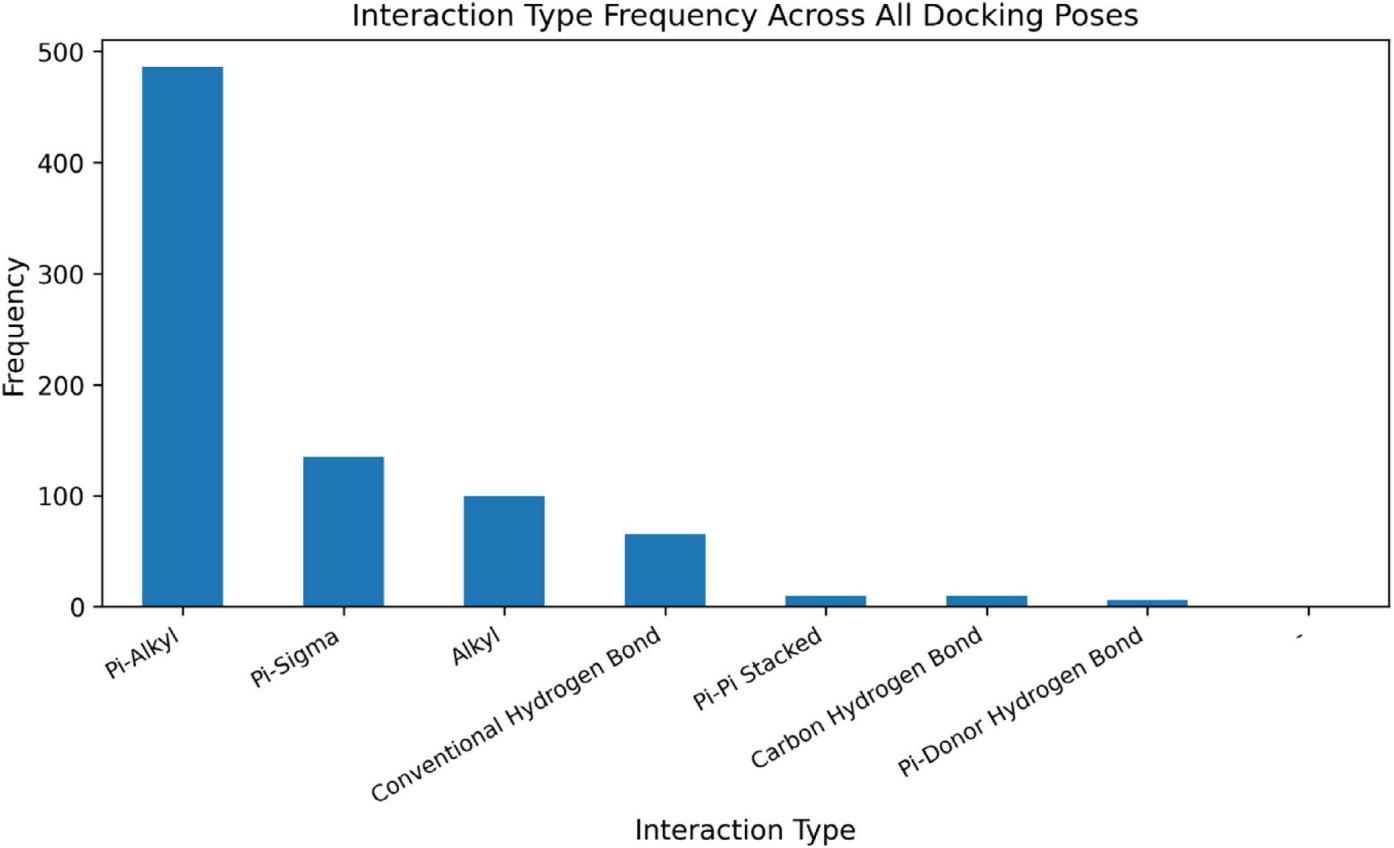
Frequency of molecular interactions between essential oil constituents and target proteins based on docking analysis.

Across all ligand–enzyme complexes, the best docking modes exhibited RMSD values generally < 2.0 Å, indicating high conformational reliability and consistency with reference conformations. Germacrene D, in particular, achieved a perfect alignment (0.000 Å RMSD) in complexes with AChE and α‐amylase, whereas α‐ and β‐pinene demonstrated deviations up to 19 Å in certain combinations, suggesting lower conformational stability. The detailed interaction data presented in Table 6 clearly illustrate germacrene D's superiority as a potential multitarget inhibitor, while also highlighting the promising aromatic interaction profiles of the pinene isomers as pharmacophoric scaffolds. The lowest binding energies for all 45 compounds are summarized in Table 5; a comprehensive heatmap of these values is shown in Figure 8, and the overall frequency distribution of interaction types in Figure 9. Full 2D diagrams, 3D poses, and surface maps for each ligand–enzyme pair are provided in Detailed residue–ligand interaction lists available in the Supporting Information as Table S1. A molecular docking‐based heatmap representing the interaction energies of essential oil compounds with target enzymes and the frequency of molecular interactions between essential oil constituents and target proteins based on docking analysis was presented in Figures 8 and 9.

Previous reports have established that donepezil binds to AChE through interactions with TRP86, PHE338, and TYR337 (Hakeem 2023). In this study, germacrene D was similarly observed to form interactions with these critical residues, supporting its potential as a potent AChE inhibitor. Likewise, germacrene D replicated the hydrogen bonding interactions of acarbose with ASP197 and GLU233 in α‐amylase, reinforcing its functional binding capability (Tables 6 and 7). Discovery Studio analyses further revealed that germacrene D possessed a significantly larger interaction surface area compared to α‐ and β‐pinene.

**TABLE 7:** Hydrogen bond lengths for germacrene D–enzyme complexes.

Enzyme	Residues	Bond length (Å)
AChE (4EY7)	GLU202	3.2
BChE (5DYW)	SER198	3.5
α‐Amylase (2QV4)	ASP197, GLU233	2.8–3.1
α‐Glucosidase (3A4A)	ARG526, ASP404	2.7–3.3

Due to their small and apolar structures, α‐pinene and β‐pinene were limited to forming superficial hydrophobic interactions. Although they achieved lower binding energies, they were able to establish π–alkyl and π–sigma interactions with aromatic residues, thereby maintaining a degree of binding stability. For instance, α‐pinene's hydrophobic contacts with TRP86 and TYR337 in AChE highlight its capacity to access critical active site regions despite its limited size (Table 8). Nevertheless, the inability of pinene isomers to form hydrogen bonds reduced the overall stability of their complexes.

**TABLE 8:** Hydrophobic interactions of α‐ and β‐pinene.

Ligand	Enzyme	Interaction count	Key residues
α‐Pinene	AChE (4EY7)	2	TRP86, TYR337
β‐Pinene	BChE (5DYW)	3	TRP82, TYR332

## 4 Discussion

### 4.1 Essential Oil

GC‐FID and GC–MS analysis of *A. chamaepitys* essential oil identified key compounds: ethyl linoleate (13.7%), germacrene D (13.4%), kaurene (8.4%), β‐pinene (6.8%), and (E)‐phytol (5.3%), revealing its complex chemical profile (Venditti et al. 2016). The essential oil of *A. chamaepitys* was obtained by hydrodistillation and analyzed by GC and GC–MS, identifying 25 compounds. Monoterpene hydrocarbons made up 65.6%, with α‐pinene (16.1%) and β‐pinene (34.3%) as the main components, while sesquiterpenes accounted for 18.0%, with germacrene D (5.6%) as the major constituent (Azizan et al. 2002). GC–MS and GC‐FID analyses identified the major components of the essential oil from the aerial parts of the plant as α‐pinene (23.66%), β‐pinene (9.33%), 1‐octen‐3‐ol (9.72%), β‐phellandrene (8.70%), and germacrene D (7.92%) (Delazar et al. 2012). The essential oil composition of *A. chamaepitys* (L.) Schreber ssp. *chia* (Schreber) Arcangeli from the aerial parts collected on Vidlic Mountain (Serbia) under normal (NEC) and post‐fire (PFEC) conditions was analyzed by GC and GC–MS. Thirty‐eight compounds were identified in the NEC oil (98.3%) and 34 in the PFEC oil (98.2%). Major constituents included α‐pinene (10.9%, 5.4%), β‐pinene (22.0%, 14.8%), and germacrene D (16.2%, 26.8%), (6.0%, 5.7%) (Mitić et al. 2011). Our results align with previous studies on *A. chamaepitys* essential oil, identifying key compounds such as α‐pinene, β‐pinene, and germacrene D. The consistent presence of these compounds, particularly in the monoterpene and sesquiterpene fractions, underscores the chemical stability and complexity of the oil.

### 4.2 Enzyme Inhibition Assays

In a study, the enzyme inhibitory effect of *A. chamaepitys* subsp. *chia* var. *chia* and *Ajuga bombycina* extracts was evaluated. Enzyme inhibition studies targeted AChE, BChE, *α*‐amylase, and *α*‐glucosidase. The enzyme inhibition results indicate that the ethyl acetate extracts of both *A. chamaepitys* and *A. bombycina* exhibited the strongest inhibitory activity against *α*‐amylase and *α*‐glucosidase, with *A. chamaepitys* showing the highest *α*‐glucosidase inhibition (3.56 mmol ACAE/g extract). For cholinesterase inhibition, *A. chamaepitys* ethyl acetate extract showed the highest BChE inhibition (1.44 mg GALAE/g extract), while its methanol extract displayed the highest AChE inhibition (1.52 mg GALAE/g extract). Notably, the aqueous extracts of both plants exhibited the lowest enzyme inhibition, showing no activity against AChE and BChE and only weak inhibition against *α*‐amylase and *α*‐glucosidase (Llorent‐Martínez et al. 2019).

Another study explored the antidiabetic and anti‐Alzheimer's activities of the essential oil and extracts (ethanol and water) of *Ajuga chamaecistus* subsp. *scoparia*. The essential oil exhibited the strongest enzyme inhibitory activity, particularly against *α*‐glucosidase (4.3 mmol ACAE/g), *α*‐amylase (2.8 mmol ACAE/g), acetylcholinesterase (1.96 mg GALAE/g), and butyrylcholinesterase (2.2 mg GALAE/g), while the ethanol and water extracts showed moderate inhibition (Movahhedin et al. 2016).

Our study on *A. chamaepitys* subsp. *chia* showed no *α*‐glucosidase inhibition, unlike Llorent‐Martínez et al. (2019), who reported strong inhibition for the ethyl acetate extract. However, our methanolic extract exhibited moderate *α*‐amylase and AChE inhibition. Compared to Movahhedin et al. (2016), where *A. chamaecistus* subsp. *scoparia* essential oil showed strong enzyme inhibition; our extracts demonstrated lower activity, highlighting the impact of extraction methods on bioactivity.

Overall, *A. chamaepitys* subsp. *chia* extracts exhibited moderate α‐amylase and AChE inhibition but did not show *α*‐glucosidase inhibition. The methanolic extract demonstrated slightly higher activity than the aqueous extract in all assays. Compared to standard inhibitors, the extracts displayed relatively weak activity, suggesting that while they may possess some potential in inhibiting cholinesterase enzymes, their antidiabetic effects are limited. Further investigations, including compound isolation and mechanistic studies, may help identify the bioactive constituents responsible for these activities.

### 4.3 Antioxidant Activity and Total Phenolic/Tannin Compound Test Results

The essential oil composition of the aerial parts of *A. chamaepitys* (L.) Schreber ssp. *chia* (Schreber) Arcangeli, collected from two neighboring sites on Vidlic Mountain (Serbia) under normal (NEC) and post‐fire environmental conditions (PFEC), was analyzed. The antioxidant capacity of the essential oils was evaluated using electron transfer‐based assays, including the Folin–Ciocalteu method, DPPH radical scavenging assay, and Fe^+3^ to Fe^+2^ reducing power assay. Both oil samples demonstrated moderate efficiency in reducing Fe^+3^ to Fe^+2^ and Mo^+6^ to Mo^+5^, suggesting their role as electron donors in redox reactions. However, their ability to scavenge free radicals was relatively weak (Mitić et al. 2011).

A study by Savaş et al. (2023) examined the antibiofilm, time‐kill kinetics, and antioxidant activity of *A. chamaepitys* ssp. *chia* extracts. Antioxidant activity was evaluated using the DPPH radical scavenging and reducing power assays, with the ethyl acetate extract exhibiting the highest activity. These findings suggest that *A. chamaepitys* extracts have potential as natural antioxidants and antimicrobial agents (Savaş et al. 2023).

The antioxidant, antimicrobial, and phytochemical properties of *A. chamaepitys* subsp. have been investigated. Five extracts from the aerial flowering parts were obtained using water, methanol, acetone, ethyl acetate, and petroleum ether. The ethyl acetate extract exhibited the highest total phenolic (57.02 mg GA/g) and flavonoid (91.76 mg RU/g) contents. Antioxidant activity, assessed via the DPPH assay, was strongest in the acetone extract (SC₅₀ = 330.52 μg/mL). Antimicrobial activity was evaluated using a microdilution method, with the acetone extract showing the highest efficacy against *Bacillus cereus* (MIC/MMC = 1.25 mg/mL). These findings highlight *A. chamaepitys* as a potential source of bioactive natural compounds (Jakovljević et al. 2015).

The antioxidant and antimicrobial activities of methanol, water, and chloroform extracts of *A. chamaepitys* have been evaluated. Antioxidant capacity was assessed using ABTS, DPPH, superoxide anion, hydrogen peroxide scavenging, and metal chelation assays, with comparisons to synthetic antioxidants (BHA, BHT, and α‐tocopherol). The water extract exhibited the highest antioxidant activity. Total phenolic content was measured as pyrocatechol equivalents. Antimicrobial activity, tested via the disc diffusion method against six bacterial strains and two yeasts, demonstrated the bioactive potential of *A. chamaepitys* . These findings suggest its value as a natural antioxidant source (Turkoglu et al. 2010).

### 4.4 Morphological Study and Anatomical Studies

Two main varieties of *A. chamaepitys* are identified: *var. grandiflora*, which has larger flowers, and *var. glabra*, characterized by puberulous stems. These varieties are common in Europe, especially in Hungary, where the nutlets are reticulately pitted. The study examines their morphological variations across several regions: **Italy and Sicily**: Material from these areas includes *var. grandiflora* and *var. glabriuscula*, with some specimens having woody rootstocks. **North Africa**: Specimens similar to *var. grandiflora* are found in Morocco, though records from Algeria are sparse. **Bessarabia and South Russia**: Material shows a mix of *A. chamaepitys* and *A. chia*, with nutlets exhibiting both types of markings. **Crimea**: Specimens show characteristics of *A. chia*, with distinctive nutlets and larger flowers. **Iberian Peninsula**: The material exhibits high polymorphism, with plants showing traits of both *A. chamaepitys* and *A. chia*. **Greece**: Larger flowers and woody rootstocks are observed in southern Greece, where plants are often classified as *A. chia*. **Albania and Macedonia**: Most material is classified as *A. chamaepitys var. grandiflora*, though one specimen from South Macedonia may be *A. chia*. **Thrace**: Material from the Gallipoli Peninsula fits the characteristics of *A. chia*, with larger flowers. **Bulgaria and Dobruja**: Specimens show intermediate characteristics between *A. chamaepitys* and *A. chia*. **Serbia**: Material from Niš is classified as *A. chamaepitys var. grandiflora*. **Bosnia and Herzegovina**: Typical *A. chamaepitys* traits are observed in the specimens. **Dalmatia**: Variation between *A. chamaepitys* var. *grandiflora* and var. *glabra* is common. **Istria**: Specimens conform to *A. chamaepitys* var. *grandiflora*. **Asia Minor, Syria, and Palestine**: Material from these regions aligns with *A. chia*, characterized by larger flowers and perennial growth (Turrill 1934).

Talebi et al. (2019) conducted a study on the nutlet morphology of six Iranian Ajuga taxa, utilizing Scanning Electron Microscopy and Light Microscopy to analyze 13 nutlet characteristics. The study found significant variation (*p* < 0.01) in quantitative features among the taxa, with Principal Component Analysis (PCA) revealing that a few key traits explained over 60% of the variation. Stable features like nutlet shape contrasted with highly variable traits, such as dorsal sculpturing and exocarp cell indumentum, which served as distinguishing markers for the taxa. The study also divided the taxa into four groups based on the morphological data, challenging previous classifications in Flora Iranica and suggesting a reevaluation of some infraspecific taxa. Talebi et al. (2019) recommend complementary molecular studies to clarify species relationships and taxonomic positions within the genus.

The study conducted by Ulcay (2021) aimed to explore the morphological, anatomical, and ecological characteristics of *A. salicifolia* (L.) Schreber, a species from the Lamiaceae family. Samples were collected from Bağbaşı, Kırşehir, between April and June 2019. The findings revealed that the plant has an upright, occasionally spreading stem with red coloration and hispid hairs. Its leaves are oblong‐lanceolate, and the yellow corolla has prominent red spots. Anatomically, the root features a rectangular periderm and distinct xylem, while the stem has circular epidermal cells with glandular and eglandular hairs and continuous vascular bundles surrounded by sclerenchyma. The plant thrives in clayey, slightly alkaline soils rich in potassium and organic matter. The study provides valuable insights into the morphology and anatomy of Ajuga salicifolia, contributing to future research on the *Ajuga* genus.

The research explores the anatomical and chemical properties of two *Ajuga* species ( *A. genevensis* L. and *A. reptans* L.) from different populations in Romania. The focus is on examining both the intra‐ and interspecific variation in their histo‐anatomical and chemical features. Chemical analysis was carried out using techniques such as TLC and UV–VIS spectrophotometry to quantify iridoids, flavonoids, and polyphenolic acids (Hemcinschi et al. 2009).

The leaf anatomy of three subspecies of *A. chamaecistus* Ging. ex Benth. (Lamiaceae), a small subshrub endemic to Iran, was studied in this research. The specimens were collected from 28 regions across 16 provinces. The study examined both qualitative traits, such as leaf type, trichome type, and vascular structures, as well as quantitative traits, including stomatal density, cuticle thickness, and mesophyll dimensions. Despite some separation of the subspecies through PCA, the anatomical traits were largely similar and insufficient for distinguishing the subspecies on their own. The study suggests that additional anatomical research on other plant structures, such as the petiole and stem, alongside further biosystematic studies, would be necessary for clearer differentiation (Kazemi Saeed et al. 2019).

Drawing on a thorough review of existing literature, this study is the first to provide a comprehensive analysis of both the anatomical and morphological characteristics of *Ajuga chamaepitys* (L.) Schreb. subsp. *chia* (Schreb.) Arcang (Lamiaceae). While earlier studies have primarily focused on specific aspects of the plant, such as its ecological distribution or chemical composition, our research integrates detailed anatomical findings with morphological observations. By examining key structural features, including leaf morphology, trichome types, vascular organization, and stomatal traits, this study offers valuable new insights into the plant's chemical profile, biological activities, and overall morpho‐anatomical attributes. The findings expand our understanding of its functional adaptations and ecological significance.

### 4.5 Molecular Docking Studies

Hydrogen bonds are critical for stabilizing ligand–enzyme complexes. The low polarity and internal ring strain of monoterpenes (α−/β‐pinene), characterized by a single isoprene double bond and a tertiary hydrogen surface, limit their capacity to form hydrogen bonds with catalytic acid–base residues (Lu et al. 2024). In contrast, germacrene D's sesquiterpene nature, enriched with additional methylene groups and a conjugated double bond system, offers a broader hydrophobic surface while retaining the potential for hydrogen bonding via its oxygenated isoprene segment (Bülow and König 2000).

As presented in Table 9, germacrene D distinguished itself by exhibiting a 16.7% hydrogen bond contribution to its interaction profile, while α‐ and β‐pinene interactions were almost exclusively hydrophobic (> 80% alkyl + π‐alkyl). Furthermore, Table 10 shows that germacrene D formed a balanced interaction pattern consisting of eight hydrophobic and three hydrogen bonds, whereas pinene isomers primarily relied on hydrophobic contacts. The hydrogen bond lengths for germacrene D complexes ranged between 2.7 and 3.5 Å, indicative of optimal bonding strength and contributing to both binding affinity and stability.

**TABLE 9:** Percentage distribution of interaction subtypes.

Ligand	Alkyl (%)	π‐Alkyl (%)	π‐Sigma (%)	Hydrogen bond (%)
α‐Pinene	22.2	66.7	11.1	0.0
β‐Pinene	14.3	57.1	28.6	0.0
Germacrene D	0.0	66.7	16.7	16.7

**TABLE 10:** Number of hydrophobic and hydrogen bonds per ligand.

Ligand	Hydrophobic	Hydrogen
α‐Pinene	35	0
β‐Pinene	17	0
Germacrene D	8	3

Overall (Table 5), germacrene D achieved 77% and 84% of donepezil's binding scores against AChE and BChE, respectively, and 89% and 83% of acarbose's binding scores against α‐amylase and α‐glucosidase. These results suggest that germacrene D exhibits clinically meaningful inhibitory potential for a natural compound and may serve as a valuable lead for multitarget drug development efforts. Conversely, α‐ and β‐pinene, despite failing to bridge the binding affinity gap with reference inhibitors due to their limited hydrogen bonding capacity, demonstrated significant π‐alkyl and π‐sigma interactions within the aromatic gorge, preserving their potential as pharmacophoric scaffolds.

Although the biological activities of the tested extracts and essential oils were found to be moderate compared to standard compounds, this outcome is not unexpected. Crude extracts consist of complex mixtures in which bioactive constituents may be present at low concentrations, or their effects may be masked by other components. Therefore, their overall potency often appears lower than that of isolated compounds. Nonetheless, the observed antioxidant, antidiabetic, and anticholinesterase activities are in line with the traditional uses of *A. chamaepitys* subsp. *chia*, particularly for mild therapeutic purposes. Moreover, the relatively high concentrations required for measurable inhibition suggest the potential for dose‐dependent responses, which could be enhanced through further fractionation and isolation. Importantly, molecular docking analyses revealed strong binding affinities of key compounds, such as germacrene D, to multiple target enzymes, indicating a promising multitarget therapeutic potential. These docking findings will provide a valuable foundation for future 100 ns molecular dynamics simulations and MM/GBSA free‐energy calculations to more deeply assess complex stability and binding affinity. Ultimately, these insights offer a solid basis for future bioactivity‐guided isolation studies and in vivo validations to better assess the pharmacological relevance of this traditionally used plant.

## 5 Conclusion

In conclusion, *Ajuga chamaepitys* subsp. *chia* demonstrates significant antioxidant, antidiabetic, and anticholinesterase activities, with the methanolic extract showing the highest bioactivity, particularly in scavenging ABTS^•+^ and DPPH^•^ radicals and inhibiting α‐amylase and acetylcholinesterase enzymes. The essential oil composition, dominated by monoterpene and sesquiterpene hydrocarbons, further supports its potential therapeutic value. Additionally, the plant's morphological and anatomical characteristics, including its cuticle‐covered leaves with glandular trichomes and starch‐filled parenchyma in the stem, highlight its unique structure. Furthermore, germacrene D formed highly stable complexes across all four enzyme targets, characterized by low RMSD values and extensive hydrogen bonding interactions, suggesting its strong candidacy for future in vitro and in vivo validation studies. Meanwhile, α‐ and β‐pinene, due to their compact molecular frameworks, offer valuable starting points for semi‐synthetic modification. Structural derivatization, particularly through the introduction of polar functional groups, is expected to enhance their binding affinities and pharmacokinetic properties. Moreover, the design of hybrid molecules combining sesquiterpene and monoterpene cores (e.g., a germacrene backbone integrated with a hydroxylated pinane ring) represents a promising strategy for the development of multitarget anti‐Alzheimer's and antidiabetic agents. These findings not only validate its traditional uses in Türkiye for wound healing and hemorrhoid treatment but also suggest that *A. chamaepitys* subsp. *chia* may serve as a valuable natural source for developing novel therapeutic agents targeting oxidative stress, diabetes, and Alzheimer's disease.

## Author Contributions

**Amine Sena Aydın:** data curation (equal), investigation (equal), methodology (equal), writing – original draft (equal). **Bilge Aydın:** conceptualization, formal analysis, investigation (equal), methodology (equal), writing – original draft. **Ömer Çeçen:** conceptualization (equal), formal analysis (equal), investigation (equal), methodology (equal), resources. **Hafize Yuca:** conceptualization (equal), project administration, methodology (equal), resources, writing – original draft. **Gülnur Ekşi Bona:** conceptualization (equal), formal analysis (equal), investigation (equal), methodology (equal). **Betül Demirci:** conceptualization (equal), formal analysis (equal), investigation (equal), methodology (equal). **Mehmet Bona:** conceptualization (equal), investigation (equal), methodology (equal), formal analysis (equal). **Songul Karakaya:** conceptualization (equal), methodology (equal), writing – review and editing.

## Disclosure

The authors have nothing to report.

## Ethics Statement

The authors have nothing to report.

## Conflicts of Interest

The authors declare no conflicts of interest.

## Supporting information


**Table S1:** Residue ligand interactions.

## Data Availability

The data supporting the findings of this study are available from the corresponding author upon reasonable request.

## References

[fsn370749-bib-0001] Adib, M. , F. Peytam , M. Rahmanian‐Jazi , et al. 2018. “New 6‐Amino‐Pyrido [2, 3‐d] Pyrimidine‐2, 4‐Diones as Novel Agents to Treat Type 2 Diabetes: A Simple and Efficient Synthesis, α‐Glucosidase Inhibition, Molecular Modeling and Kinetic Study.” European Journal of Medicinal Chemistry 155: 353–363. 29902721 10.1016/j.ejmech.2018.05.046

[fsn370749-bib-0002] Akshatha, J. V. , H. S. SantoshKumar , H. S. Prakash , and M. S. Nalini . 2021. “In Silico Docking Studies of α‐Amylase Inhibitors From the Anti‐Diabetic Plant Leucas Ciliata Benth. And an Endophyte, *Streptomyces longisporoflavus* .” 3 Biotech 11, no. 2: 51. 10.1007/s13205-020-02547-0 PMC7801551 33489670

[fsn370749-bib-0003] Ammar, H. , I. Touihri , A. E. Kholif , et al. 2022. “Chemical Composition, Antioxidant, and Antimicrobial Activities of Leaves of *Ajuga iva* .” Molecules 27, no. 20: 7102. 36296695 10.3390/molecules27207102 PMC9607272

[fsn370749-bib-0004] Aslan, S. , H. Akan , and H. Pekmez . 2020. “Yaslıca Beldesi ve Arıkök Mahallesi (Şanlıurfa)’nin Etnobotanik Açıdan Araştırılması.” Biological Diversity and Conservation 13, no. 1: 44–61.

[fsn370749-bib-0005] Azizan, J. , H. Fallah‐Bagher‐Shaidaei , and H. Kefayati . 2002. “Chemical Constituents of the Essential Oil of *Ajuga chamaepitys* Growing in Iran.” Journal of Essential Oil Research 14, no. 5: 344–345.

[fsn370749-bib-0006] Bachhawat, J. A. , M. S. Shihabudeen , and K. Thirumurugan . 2011. “Screening of Fifteen Indian Ayurvedic Plants for Alpha‐Glucosidase Inhibitory Activity and Enzyme Kinetics.” International Journal of Pharmacy and Pharmaceutical Sciences 3, no. 4: 267–274.

[fsn370749-bib-0007] BIOVIA . 2021. Dassault Systèmes. Discovery Studio Visualizer. v21.1.0.20298. Dassault Systèmes.

[fsn370749-bib-0008] Blois, M. S. 1958. “Antioxidant Determinations by the Use of a Stable Free Radical.” Nature 181, no. 4617: 1199–1200. 10.1038/1811199a0.

[fsn370749-bib-0009] Bülow, N. , and W. A. König . 2000. “The Role of Germacrene D as a Precursor in Sesquiterpene Biosynthesis: Investigations of Acid Catalyzed, Photochemically and Thermally Induced Rearrangements.” Phytochemistry 55, no. 2: 141–168. 11065290 10.1016/s0031-9422(00)00266-1

[fsn370749-bib-0010] Carlsson, C. M. 2010. “Type 2 Diabetes Mellitus, Dyslipidemia, and Alzheimer's Disease.” Journal of Alzheimer's Disease 20: 711–722. 10.3233/JAD-2010-100012. PMC4235787 20413858

[fsn370749-bib-0011] Delazar, A. , M. R. Delnavazi , N. Yassa , et al. 2012. “Essential Oil Composition and Isolation of Free‐Radical‐Scavenging Phenolic Glycosides From the Aerial Parts of *Ajuga chamaepitys* Growing in Iran.” Revista Brasileira de Farmacognosia 22: 399–405.

[fsn370749-bib-0012] Eberhardt, J. , D. Santos‐Martins , A. F. Tillack , and S. Forli . 2021. “AutoDock Vina 1.2.0: New Docking Methods, Expanded Force Field, and Python Bindings.” Journal of Chemical Information and Modeling 61, no. 8: 3891–3898. 34278794 10.1021/acs.jcim.1c00203 PMC10683950

[fsn370749-bib-0013] Firoskhan, N. , and R. Muthuswamy . 2021. “Review on *Maranta arundinacea* L. (Marantacea).” International Journal of Pharmacognosy and Pharmaceutical Research 3, no. 1: 1–4.

[fsn370749-bib-0014] Folin, O. , and W. Denis . 1912. “On Phosphotungstic‐Phosphomolybdic Compounds as Color Reagents.” Journal of Biological Chemistry 12, no. 2: 239–243.

[fsn370749-bib-0015] Hakeem, I. J. 2023. “Molecular Docking Analysis of Acetylcholinesterase Inhibitors for Alzheimer's Disease Management.” Bioinformation 19, no. 5: 565–570. 37886145 10.6026/97320630019565 PMC10599677

[fsn370749-bib-0016] Hemcinschi, A. , R. Gales , U. Stanescu , and C. Toma . 2009. “Comparative Histo‐Anatomy and Chemical Composition of Two *Ajuga* Species From the Romanian Flora.” Analele Ştiinţifice Ale Universităţii “Al. I. Cuza” Din Iaşi 55, no. 2: 33.

[fsn370749-bib-0017] Hochmuth, D. H. 2008. MassFinder 4.0. Hochmuth Scientific Consulting.

[fsn370749-bib-0018] Ingkaninan, K. , C. M. de Best , R. van der Heijden , et al. 2000. “High‐Performance Liquid Chromatography With On‐Line Coupled UV, Mass Spectrometric and Biochemical Detection for Identification of Acetylcholinesterase Inhibitors From Natural Products.” Journal of Chromatography A 872, no. 1–2: 61–73. 10749487 10.1016/s0021-9673(99)01292-3

[fsn370749-bib-0019] Ionus, E. , L. A. Bucur , C. E. Lupu , and C. E. Gȋrd . 2021. “Evaluation of the Chemical Composition of *Ajuga chamaepitys* (L.) Schreb. From the Spontaneous Flora of Romania.” Farmácia 69, no. 3: 461–466.

[fsn370749-bib-0020] İzol, E. 2025. “Molecular Docking‐Guided In‐Depth Investigation of the Biological Activities and Phytochemical and Mineral Profiles of Endemic *Phlomis capitata* .” Journal of the Science of Food and Agriculture 105, no. 7: 3760–3775. 10.1002/jsfa.14142. 39876756 PMC11990054

[fsn370749-bib-0021] İzol, E. , M. Turhan , M. A. Yılmaz , C. Çağlayan , and İ. Gülçin . 2025. “Determination of Antioxidant, Antidiabetic, Anticholinergic, Antiglaucoma Properties and Comprehensive Phytochemical Content by LC‐MS/MS of Bingöl Honeybee Pollen.” Food Science & Nutrition 13, no. 3: e4531. 10.1002/fsn3.4531. I congratulate the authors for their study. 40041710 PMC11876561

[fsn370749-bib-0022] İzol, E. , M. A. Yılmaz , and İ. Gülçin . 2025. “Chemical Characterization by Chromatography Techniques and Comprehensive Biological Activities of Artvin Bee Products.” ChemistrySelect 10, no. 18: e202501545. 10.1002/slct.202501545.

[fsn370749-bib-0023] Jaffal, S. M. , M. A. Abbas , M. Alsalem , and B. O. Al‐Najjar . 2019. “Evidence for the Involvement of Opioid Receptor in *Ajuga chamaepitys* Action in Chemical and Thermal Models of Pain in BALB/c Mice.” Medicinal Chemistry Research 28: 992–999.

[fsn370749-bib-0024] Jakovljević, D. , S. Vasić , M. Stanković , L. Čomić , and M. Topuzović . 2015. Secondary Metabolite Content and In Vitro Biological Effects of *Ajuga chamaepitys* (L.) Schreb. Subsp. *chamaepitys* . 10.1556/018.66.2015.4.4 26616372

[fsn370749-bib-0025] Júnior, A. Q. D. S. , G. D. S. Rodrigues , A. D. S. Barroso , et al. 2025. “Essential Oil of Lippia Origanoides Kunth: Nanoformulation, Anticholinesterase Activity, and Molecular Docking.” Molecules 30, no. 7: 1554. 10.3390/molecules30071554. 40286153 PMC11990080

[fsn370749-bib-0026] Karakaya, S. , H. Yuca , G. Yilmaz , et al. 2023. “Phytochemical Screening, Biological Evaluation, Anatomical, and Morphological Investigation of *Ferula tingitana* L. (Apiaceae).” Protoplasma 260, no. 6: 1581–1601. 10.1007/s00709-023-01874-2. 37338647

[fsn370749-bib-0027] Karrouchi, K. , S. A. Brandán , Y. Sert , et al. 2020. “Synthesis, X‐Ray Structure, Vibrational Spectroscopy, DFT, Biological Evaluation and Molecular Docking Studies of (E)‐N'‐(4‐(Dimethylamino) Benzylidene)‐5‐Methyl‐1H‐Pyrazole‐3‐Carbohydrazide.” Journal of Molecular Structure 1219: 128541.

[fsn370749-bib-0028] Kazemi Saeed, F. , Z. Jamzad , A. Vaziri , A. Jalil , and F. Sefidkon . 2019. “Foliar Anatomical Studies of *Ajuga chamaecistus* (Lamiaceae) From Iran.” Iranian Journal of Botany 25, no. 2: 152–181.

[fsn370749-bib-0053] Liu, M. , X. Gong , R. K. Alluri , J. Wu , and Z. Li . 2012. “Characterization of RNA Damage Under Oxidative Stress in Escherichia coli.” Biological Chemistry 393, no. 3: 123. 10.1515/hsz-2011-0247. 22718628 PMC3404489

[fsn370749-bib-0029] Llorent‐Martínez, E. J. , G. Zengin , P. Ortega‐Barrales , et al. 2019. “Characterization of the Phytochemical Profiles and Biological Activities of *Ajuga chamaepitys* Subsp. *chia* var. *chia* and *Ajuga bombycina* by High‐Performance Liquid Chromatography–Electrospray Ionization–Tandem Mass Spectrometry (HPLC–ESI–MSn).” Analytical Letters 52, no. 5: 852–868. 10.1080/00032719.2018.1500581.

[fsn370749-bib-0030] Lu, X. , J. Bai , Z. Tian , et al. 2024. “Cyclization Mechanism of Monoterpenes Catalyzed by Monoterpene Synthases in Dipterocarpaceae.” Synthetic and Systems Biotechnology 9, no. 1: 11–18. 38173809 10.1016/j.synbio.2023.11.009 PMC10758623

[fsn370749-bib-0031] Makkar, H. P. , and H. P. Makkar . 2003. “Measurement of Total Phenolics and Tannins Using Folin‐Ciocalteu Method.” In Quantification of Tannins in Tree and Shrub Foliage: A Laboratory Manual, 49–51. 10.1007/978-94-017-0273-7_3. Springer Netherlands.

[fsn370749-bib-0032] McLafferty, F. W. , and D. B. Stauffer . 1989. The Wiley/NBS Registry of Mass Spectral Data. J. Wiley & Sons.

[fsn370749-bib-0033] Mitić, V. D. , V. P. Stankov‐Jovanović , O. P. Jovanović , I. R. Palić , A. S. Djordjević , and G. S. Stojanović . 2011. “Composition and Antioxidant Activity of Hydrodistilled Essential Oil of Serbian *Ajuga chamaepitys* (L.) Schreber ssp. *chia* (Schreber) Arcangeli.” Journal of Essential Oil Research 23, no. 6: 70–74.

[fsn370749-bib-0034] Moroz, N. , M. Tong , L. Longato , H. Xu , and S. M. de la Monte . 2008. “Limited Alzheimer‐Type Neurodegeneration in Experimental Obesity and Type 2 Diabetes Mellitus.” Journal of Alzheimer's Disease 15, no. 1: 29–44. 10.3233/jad-2008-15103 PMC10074393 18780965

[fsn370749-bib-0035] Movahhedin, N. , G. Zengin , M. B. Bahadori , C. Sarikurkcu , S. Bahadori , and L. Dinparast . 2016. “ *Ajuga chamaecistus* Subsp. *scoparia* (Boiss.) Rech. f.: A New Source of Phytochemicals for Antidiabetic, Skin‐Care, and Neuroprotective Uses.” Industrial Crops and Products 94: 89–96.

[fsn370749-bib-0036] Nampoothiri, S. V. , A. Prathapan , O. L. Cherian , K. G. Raghu , V. V. Venugopalan , and A. Sundaresan . 2011. “In Vitro Antioxidant and Inhibitory Potential of *Terminalia bellerica* and *Emblica officinalis* Fruits Against LDL Oxidation and Key Enzymes Linked to Type 2 Diabetes.” Food and Chemical Toxicology 49, no. 1: 125–131. 20951180 10.1016/j.fct.2010.10.006

[fsn370749-bib-0054] Nicolai, M. , P. Pereira , R. F. Vitor , C. P. Reis , A. Roberto , and P. Rijo . 2016. “Antioxidant Activity and Rosmarinic Acid Content of Ultrasound‐Assisted Ethanolic Extracts of Medicinal Plants.” Measurement 89: 328–332. 10.1016/j.measurement.2016.04.033.

[fsn370749-bib-0037] Peytam, F. , G. Takalloobanafshi , T. Saadattalab , et al. 2021. “Design, Synthesis, Molecular Docking, and In Vitro α‐Glucosidase Inhibitory Activities of Novel 3‐Amino‐2, 4‐Diarylbenzo [4, 5] Imidazo [1, 2‐a] Pyrimidines Against Yeast and Rat α‐Glucosidase.” Scientific Reports 11, no. 1: 11911. 34099819 10.1038/s41598-021-91473-z PMC8184976

[fsn370749-bib-0038] Re, R. , N. Pellegrini , A. Proteggente , A. Pannala , M. Yang , and C. Rice‐Evans . 1999. “Antioxidant Activity Applying an Improved ABTS Radical Cation Decolorization Assay.” Free Radical Biology and Medicine 26, no. 9–10: 1231–1237. 10.1016/S0891-5849(98)00315-3. 10381194

[fsn370749-bib-0039] Savaş, A. N. , N. Hacıoğlu , and N. Demir . 2023. “Investigation of Some Biological Activities of *Ajuga chamaepitys* Subsp. *chia* .” Journal of the Institute of Science and Technology 13, no. 1: 162–169.

[fsn370749-bib-0040] Selvi, S. , R. Polat , U. Çakılcıoğlu , F. Celep , T. Dirmenci , and Z. F. Ertuğ . 2022. “An Ethnobotanical Review on Medicinal Plants of the Lamiaceae Family in Turkey.” Turkish Journal of Botany 46, no. 4: 1. 10.55730/1300-008X.2712.

[fsn370749-bib-0041] Shakil, S. 2021. “Molecular Interaction of Inhibitors With Human Brain Butyrylcholinesterase.” EXCLI Journal 20: 1597–1607. 35024017 10.17179/excli2021-4418 PMC8743831

[fsn370749-bib-0042] Shinde, S. , S. Chavhan , S. Jain , and K. Shukla . 2021. “Pharmacognostical Study, Phytochemical and Physicochemical Evaluation and Establishment of Quality Standards for Certain Traditional Antidiabetic Medicinal Plants.” Journal of Pharmaceutical Research International 33, no. 39B: 288–299. 10.9734/JPRI/2021/v33i39B32207.

[fsn370749-bib-0043] Slinkard, K. , and V. L. Singleton . 1977. “Total Phenol Analysis: Automation and Comparison With Manual Methods.” American Journal of Enology and Viticulture 28, no. 1: 49–55. 10.5344/ajev.1977.28.1.49.

[fsn370749-bib-0044] Talebi, S. M. , R. Tabaripour , and M. Eskandari . 2019. “Analysis of Nutlet Morphological Characteristics of Some Iranian *Ajuga* L. Taxa.” Biodiversitas Journal of Biological Diversity 20, no. 10: 2833–2840.

[fsn370749-bib-0045] Trott, O. , and A. J. Olson . 2010. “AutoDock Vina: Improving the Speed and Accuracy of Docking With a New Scoring Function, Efficient Optimization, and Multithreading.” Journal of Computational Chemistry 31, no. 2: 455–461. 19499576 10.1002/jcc.21334 PMC3041641

[fsn370749-bib-0046] Turkoglu, S. , I. Turkoglu , M. Kahyaoglu , and S. Celık . 2010. “Determination of Antimicrobial and Antioxidant Activities of Turkish Endemic *Ajuga chamaepitys* (L.) Schreber Subsp. *euphratica* P. H. Davis (Lamiaceae).” Journal of Medicinal Plant Research 4, no. 13: 1260–1268.

[fsn370749-bib-0047] Turrill, W. B. 1934. “The Correlation of Morphological Variation With Distribution in Some Species of *Ajuga* .” New Phytologist 33, no. 3: 218–230.

[fsn370749-bib-0048] Ulcay, S. 2021. “Morphological, Anatomical, and Ecological Features of *Ajuga salicifolia* (L.) Schreber (Lamiaceae) With Natural Spreading.” Sakarya University Journal of Science 25, no. 1: 230–239.

[fsn370749-bib-0049] Venditti, A. , C. Frezza , F. Maggi , et al. 2016. “Phytochemistry, Micromorphology, and Bioactivities of *Ajuga chamaepitys* (L.) Schreb. (Lamiaceae, Ajugoideae): Two New Harpagide Derivatives and an Unusual Iridoid Glycosides Pattern.” Fitoterapia 113: 35–43. 27373875 10.1016/j.fitote.2016.06.016

[fsn370749-bib-0050] World Health Organization . 2011. Quality Control Methods for Herbal Materials (Updated Edition). WHO Press. https://apps.who.int/iris/handle/10665/44479.

[fsn370749-bib-0051] Yuca, H. , A. Sefalı , B. Aydın , et al. 2024. “Phytochemical Analysis and Biological Evaluation of Essential Oils and Extracts From *Heracleum pastinacifolium* Subsp. *incanum* (Boiss. & A. Huet) P. H. Davis, an Endemic Plant From Turkey.” Natural Product Research 38: 1–11. 10.1080/14786419.2024.2372661. 38962953

[fsn370749-bib-0052] Zu, G. , K. Sun , L. Li , X. Zu , T. Han , and H. Huang . 2021. “Mechanism of Quercetin Therapeutic Targets for Alzheimer Disease and Type 2 Diabetes Mellitus.” Scientific Reports 11: 22959. 10.1038/s41598-021-02379-6. 34824300 PMC8617296

