# Supplementary material for: From Herbal Tea to Science: Phytochemical and Biological Investigation of Ajuga chamaepitys subsp. chia (Lamiaceae) With Molecular Docking Analysis
Source: Food Sci Nutr. 2025 Aug 13;13(8):e70749. doi: 10.1002/fsn3.70749 (PMC12344866; doi:10.1002/fsn3.70749)
Supplement: Supplementary file 1 — Table S1: Residue ligand interactions. [file FSN3-13-e70749-s001.docx]

| **Table S1.** Residue ligand interactions. | | | | | |
| --- | --- | --- | --- | --- | --- |
| **Enzyme** | **Compound** | **Protein** | **Distance** | **Category** | **Types** |
| 2QV4 | 1,8-cineol | TYR62 | 3,71732 | Hydrophobic | Pi-Sigma |
| 2QV4 | 1,8-cineol | TYR62 | 5,22128 | Hydrophobic | Pi-Alkyl |
| 2QV4 | 1,8-cineol | HIS299 | 4,89929 | Hydrophobic | Pi-Alkyl |
| 2QV4 | Bicyclogermacrene | TRP59 | 5,18169 | Hydrophobic | Pi-Alkyl |
| 2QV4 | Borneol | GLU233 | 3,08494 | Hydrogen Bond | Conventional Hydrogen Bond |
| 2QV4 | Borneol | GLU233 | 3,13442 | Hydrogen Bond | Conventional Hydrogen Bond |
| 2QV4 | Borneol | TYR62 | 3,83486 | Hydrophobic | Pi-Sigma |
| 2QV4 | Borneol | TYR62 | 5,469 | Hydrophobic | Pi-Alkyl |
| 2QV4 | Bornyl Acetate | TYR62 | 5,29628 | Hydrophobic | Pi-Alkyl |
| 2QV4 | Calamenene | TRP59 | 4,33417 | Hydrophobic | Pi-Pi Stacked |
| 2QV4 | Calamenene | TRP59 | 3,7799 | Hydrophobic | Pi-Pi Stacked |
| 2QV4 | Calamenene | LEU165 | 5,34781 | Hydrophobic | Alkyl |
| 2QV4 | Calamenene | TRP59 | 5,04114 | Hydrophobic | Pi-Alkyl |
| 2QV4 | Calamenene | TRP59 | 3,9559 | Hydrophobic | Pi-Alkyl |
| 2QV4 | Camphene | LEU162 | 5,3881 | Hydrophobic | Alkyl |
| 2QV4 | Camphene | TYR62 | 5,46228 | Hydrophobic | Pi-Alkyl |
| 2QV4 | Camphor | TRP58 | 5,49669 | Hydrophobic | Pi-Alkyl |
| 2QV4 | Camphor | TYR62 | 5,07897 | Hydrophobic | Pi-Alkyl |
| 2QV4 | Carvone | TYR62 | 3,64611 | Hydrophobic | Pi-Sigma |
| 2QV4 | Carvone | TRP59 | 3,97696 | Hydrophobic | Pi-Sigma |
| 2QV4 | Carvone | TYR62 | 4,32482 | Hydrophobic | Pi-Alkyl |
| 2QV4 | Carvone | HIS299 | 4,75307 | Hydrophobic | Pi-Alkyl |
| 2QV4 | Caryophyllene Oxide | TRP59 | 5,20705 | Hydrophobic | Pi-Alkyl |
| 2QV4 | Epi-Cubebol | GLN63 | 3,12423 | Hydrogen Bond | Conventional Hydrogen Bond |
| 2QV4 | Germacrene-D-4-ol | ASP300 | 3,15489 | Hydrogen Bond | Conventional Hydrogen Bond |
| 2QV4 | Germacrene-D-4-ol | HIS305 | 2,99262 | Hydrogen Bond | Conventional Hydrogen Bond |
| 2QV4 | Germacrene-D-4-ol | HIS305 | 3,31537 | Hydrogen Bond | Conventional Hydrogen Bond |
| 2QV4 | Germacrene-D-4-ol | TYR62 | 3,58076 | Hydrophobic | Pi-Sigma |
| 2QV4 | Germacrene-D-4-ol | TRP59 | 4,03496 | Hydrophobic | Pi-Alkyl |
| 2QV4 | Germacrene-D-4-ol | TRP59 | 4,10666 | Hydrophobic | Pi-Alkyl |
| 2QV4 | Ledol | ASP197 | 2,90865 | Hydrogen Bond | Conventional Hydrogen Bond |
| 2QV4 | Ledol | GLU233 | 3,10236 | Hydrogen Bond | Conventional Hydrogen Bond |
| 2QV4 | Ledol | LEU162 | 4,72327 | Hydrophobic | Alkyl |
| 2QV4 | Ledol | TRP58 | 4,61488 | Hydrophobic | Pi-Alkyl |
| 2QV4 | Ledol | TRP59 | 4,60779 | Hydrophobic | Pi-Alkyl |
| 2QV4 | Ledol | TYR62 | 5,49948 | Hydrophobic | Pi-Alkyl |
| 2QV4 | Ledol | HIS305 | 4,28612 | Hydrophobic | Pi-Alkyl |
| 2QV4 | Limonene | TYR62 | 3,92117 | Hydrophobic | Pi-Sigma |
| 2QV4 | Limonene | TYR62 | 4,86744 | Hydrophobic | Pi-Alkyl |
| 2QV4 | Limonene | HIS299 | 5,23976 | Hydrophobic | Pi-Alkyl |
| 2QV4 | Linalool | ASP197 | 2,80765 | Hydrogen Bond | Conventional Hydrogen Bond |
| 2QV4 | Linalool | TYR62 | 3,68127 | Hydrophobic | Pi-Sigma |
| 2QV4 | Linalool | LEU165 | 4,75395 | Hydrophobic | Alkyl |
| 2QV4 | Linalool | LEU165 | 4,53562 | Hydrophobic | Alkyl |
| 2QV4 | Myrcene | TRP59 | 3,78611 | Hydrophobic | Pi-Sigma |
| 2QV4 | Myrcene | TRP58 | 5,21913 | Hydrophobic | Pi-Alkyl |
| 2QV4 | Myrcene | TRP59 | 5,12018 | Hydrophobic | Pi-Alkyl |
| 2QV4 | Myrcene | TRP59 | 4,21069 | Hydrophobic | Pi-Alkyl |
| 2QV4 | Myrcene | TRP59 | 5,10561 | Hydrophobic | Pi-Alkyl |
| 2QV4 | Myrcene | TYR62 | 4,85999 | Hydrophobic | Pi-Alkyl |
| 2QV4 | Myrcene | TYR62 | 3,66 | Hydrophobic | Pi-Alkyl |
| 2QV4 | Myrcene | HIS299 | 4,8214 | Hydrophobic | Pi-Alkyl |
| 2QV4 | Myrtenal | TYR62 | 3,74976 | Hydrophobic | Pi-Sigma |
| 2QV4 | Myrtenal | LEU162 | 5,44199 | Hydrophobic | Alkyl |
| 2QV4 | Myrtenol | HIS299 | 2,80374 | Hydrogen Bond | Conventional Hydrogen Bond |
| 2QV4 | Myrtenol | ASP300 | 3,16638 | Hydrogen Bond | Conventional Hydrogen Bond |
| 2QV4 | Myrtenol | ASP300 | 3,04193 | Hydrogen Bond | Conventional Hydrogen Bond |
| 2QV4 | Myrtenol | ASP197 | 3,78793 | Hydrogen Bond | Carbon Hydrogen Bond |
| 2QV4 | Myrtenol | TYR62 | 3,50508 | Hydrogen Bond | Pi-Donor Hydrogen Bond |
| 2QV4 | p-Cymene | TYR62 | 4,93708 | Hydrophobic | Pi-Pi Stacked |
| 2QV4 | p-Cymene | TYR62 | 4,00709 | Hydrophobic | Pi-Alkyl |
| 2QV4 | p-Cymene | HIS299 | 5,44304 | Hydrophobic | Pi-Alkyl |
| 2QV4 | Phytol | ASP197 | 2,71778 | Hydrogen Bond | Conventional Hydrogen Bond |
| 2QV4 | Phytol | GLU233 | 2,96753 | Hydrogen Bond | Conventional Hydrogen Bond |
| 2QV4 | Phytol | ASP300 | 3,33403 | Hydrogen Bond | Carbon Hydrogen Bond |
| 2QV4 | Phytol | LEU165 | 4,82836 | Hydrophobic | Alkyl |
| 2QV4 | Phytol | ALA106 | 3,74763 | Hydrophobic | Alkyl |
| 2QV4 | Phytol | ALA106 | 4,55702 | Hydrophobic | Alkyl |
| 2QV4 | Phytol | TRP59 | 5,38891 | Hydrophobic | Pi-Alkyl |
| 2QV4 | Phytol | TRP59 | 5,40573 | Hydrophobic | Pi-Alkyl |
| 2QV4 | Phytol | TYR62 | 4,77276 | Hydrophobic | Pi-Alkyl |
| 2QV4 | Pinocarvone | - | - | - | - |
| 2QV4 | Pulegone | TRP59 | 3,7723 | Hydrophobic | Pi-Sigma |
| 2QV4 | Pulegone | TRP59 | 3,95147 | Hydrophobic | Pi-Sigma |
| 2QV4 | Pulegone | TRP58 | 4,89255 | Hydrophobic | Pi-Alkyl |
| 2QV4 | Pulegone | TYR62 | 4,06026 | Hydrophobic | Pi-Alkyl |
| 2QV4 | Sabinene | TRP58 | 5,20049 | Hydrophobic | Pi-Alkyl |
| 2QV4 | Sabinene | TRP58 | 5,22405 | Hydrophobic | Pi-Alkyl |
| 2QV4 | Sabinene | TYR62 | 5,13364 | Hydrophobic | Pi-Alkyl |
| 2QV4 | Sabinene | TYR62 | 3,70083 | Hydrophobic | Pi-Alkyl |
| 2QV4 | Sabinene | HIS299 | 5,00054 | Hydrophobic | Pi-Alkyl |
| 2QV4 | Spathulenol | ASP197 | 2,70828 | Hydrogen Bond | Conventional Hydrogen Bond |
| 2QV4 | Spathulenol | GLU233 | 3,28568 | Hydrogen Bond | Conventional Hydrogen Bond |
| 2QV4 | Spathulenol | TYR62 | 3,88627 | Hydrophobic | Pi-Sigma |
| 2QV4 | Spathulenol | ILE235 | 5,38194 | Hydrophobic | Alkyl |
| 2QV4 | Spathulenol | LEU162 | 4,85573 | Hydrophobic | Alkyl |
| 2QV4 | Spathulenol | TYR62 | 5,01238 | Hydrophobic | Pi-Alkyl |
| 2QV4 | Spathulenol | HIS101 | 5,04557 | Hydrophobic | Pi-Alkyl |
| 2QV4 | T-Cadinol | TRP59 | 3,73498 | Hydrophobic | Pi-Sigma |
| 2QV4 | T-Cadinol | LEU165 | 4,74931 | Hydrophobic | Alkyl |
| 2QV4 | T-Cadinol | TRP59 | 3,97133 | Hydrophobic | Pi-Alkyl |
| 2QV4 | T-Cadinol | TRP59 | 4,6091 | Hydrophobic | Pi-Alkyl |
| 2QV4 | T-Cadinol | TRP59 | 5,34169 | Hydrophobic | Pi-Alkyl |
| 2QV4 | T-Cadinol | TRP59 | 4,21446 | Hydrophobic | Pi-Alkyl |
| 2QV4 | T-Cadinol | HIS305 | 4,9971 | Hydrophobic | Pi-Alkyl |
| 2QV4 | Terpinen-4-ol | TYR62 | 3,96069 | Hydrophobic | Pi-Sigma |
| 2QV4 | Terpinen-4-ol | TYR62 | 4,94516 | Hydrophobic | Pi-Alkyl |
| 2QV4 | Terpinen-4-ol | HIS299 | 5,28483 | Hydrophobic | Pi-Alkyl |
| 2QV4 | Tricosane | ILE235 | 5,15861 | Hydrophobic | Alkyl |
| 2QV4 | Tricosane | PRO54 | 3,92099 | Hydrophobic | Alkyl |
| 2QV4 | Tricosane | ILE235 | 5,12772 | Hydrophobic | Alkyl |
| 2QV4 | Tricosane | LEU237 | 4,67639 | Hydrophobic | Alkyl |
| 2QV4 | Tricosane | PRO54 | 3,95269 | Hydrophobic | Alkyl |
| 2QV4 | Tricosane | LEU237 | 4,414 | Hydrophobic | Alkyl |
| 2QV4 | Tricosane | HIS305 | 4,93123 | Hydrophobic | Pi-Alkyl |
| 2QV4 | Tricosane | HIS305 | 4,6189 | Hydrophobic | Pi-Alkyl |
| 2QV4 | Viridiflorol | TRP59 | 3,58609 | Hydrophobic | Pi-Sigma |
| 2QV4 | Viridiflorol | LEU165 | 4,90268 | Hydrophobic | Alkyl |
| 2QV4 | Viridiflorol | TRP58 | 5,35704 | Hydrophobic | Pi-Alkyl |
| 2QV4 | Viridiflorol | TRP59 | 5,21568 | Hydrophobic | Pi-Alkyl |
| 2QV4 | Viridiflorol | TRP59 | 5,44304 | Hydrophobic | Pi-Alkyl |
| 2QV4 | α-cadinol | ASP300 | 3,00514 | Hydrogen Bond | Conventional Hydrogen Bond |
| 2QV4 | α-cadinol | HIS305 | 3,12067 | Hydrogen Bond | Conventional Hydrogen Bond |
| 2QV4 | α-cadinol | HIS305 | 3,76151 | Hydrogen Bond | Pi-Donor Hydrogen Bond |
| 2QV4 | α-cadinol | TRP59 | 3,5191 | Hydrophobic | Pi-Sigma |
| 2QV4 | α-cadinol | TRP58 | 5,18765 | Hydrophobic | Pi-Alkyl |
| 2QV4 | α-cadinol | TRP59 | 5,40817 | Hydrophobic | Pi-Alkyl |
| 2QV4 | α-cadinol | TRP59 | 4,15357 | Hydrophobic | Pi-Alkyl |
| 2QV4 | α-cadinol | TRP59 | 4,23711 | Hydrophobic | Pi-Alkyl |
| 2QV4 | α-cadinol | TRP59 | 4,65305 | Hydrophobic | Pi-Alkyl |
| 2QV4 | α-copaene | ALA198 | 5,30352 | Hydrophobic | Alkyl |
| 2QV4 | α-copaene | TYR62 | 4,30042 | Hydrophobic | Pi-Alkyl |
| 2QV4 | α-cubebene | TYR62 | 3,78138 | Hydrophobic | Pi-Sigma |
| 2QV4 | α-cubebene | LEU162 | 4,78141 | Hydrophobic | Alkyl |
| 2QV4 | α-cubebene | LEU162 | 4,1868 | Hydrophobic | Alkyl |
| 2QV4 | α-cubebene | TYR62 | 4,88813 | Hydrophobic | Pi-Alkyl |
| 2QV4 | α-humulene | TRP59 | 3,82022 | Hydrophobic | Pi-Sigma |
| 2QV4 | α-humulene | TRP58 | 4,73619 | Hydrophobic | Pi-Alkyl |
| 2QV4 | α-humulene | TRP59 | 4,50451 | Hydrophobic | Pi-Alkyl |
| 2QV4 | α-humulene | TRP59 | 4,75433 | Hydrophobic | Pi-Alkyl |
| 2QV4 | α-humulene | TRP59 | 4,17634 | Hydrophobic | Pi-Alkyl |
| 2QV4 | α-humulene | TYR62 | 4,4943 | Hydrophobic | Pi-Alkyl |
| 2QV4 | α-humulene | HIS305 | 5,22572 | Hydrophobic | Pi-Alkyl |
| 2QV4 | α-muurolene | LEU165 | 4,71762 | Hydrophobic | Alkyl |
| 2QV4 | α-muurolene | LEU165 | 4,80283 | Hydrophobic | Alkyl |
| 2QV4 | α-muurolene | TRP59 | 4,43588 | Hydrophobic | Pi-Alkyl |
| 2QV4 | α-muurolene | TRP59 | 4,45111 | Hydrophobic | Pi-Alkyl |
| 2QV4 | α-muurolene | TRP59 | 4,29504 | Hydrophobic | Pi-Alkyl |
| 2QV4 | α-muurolene | TYR62 | 5,46626 | Hydrophobic | Pi-Alkyl |
| 2QV4 | α-muurolene | HIS101 | 4,89946 | Hydrophobic | Pi-Alkyl |
| 2QV4 | α-phellandrene | TYR62 | 4,75448 | Hydrophobic | Pi-Alkyl |
| 2QV4 | α-phellandrene | TYR62 | 4,07015 | Hydrophobic | Pi-Alkyl |
| 2QV4 | α-phellandrene | HIS299 | 4,91587 | Hydrophobic | Pi-Alkyl |
| 2QV4 | α-terpineol | THR163 | 3,10387 | Hydrogen Bond | Conventional Hydrogen Bond |
| 2QV4 | α-terpineol | TYR62 | 3,86833 | Hydrophobic | Pi-Sigma |
| 2QV4 | α-terpineol | TYR62 | 4,78778 | Hydrophobic | Pi-Alkyl |
| 2QV4 | α-terpineol | HIS299 | 4,89646 | Hydrophobic | Pi-Alkyl |
| 2QV4 | α-thujene | TYR62 | 3,58752 | Hydrophobic | Pi-Sigma |
| 2QV4 | α-thujene | TRP58 | 5,16264 | Hydrophobic | Pi-Alkyl |
| 2QV4 | α-thujene | TRP58 | 5,13007 | Hydrophobic | Pi-Alkyl |
| 2QV4 | α-thujene | TYR62 | 5,28282 | Hydrophobic | Pi-Alkyl |
| 2QV4 | α-thujene | HIS299 | 4,86417 | Hydrophobic | Pi-Alkyl |
| 2QV4 | β-bourbonene | TYR62 | 3,71557 | Hydrophobic | Pi-Sigma |
| 2QV4 | β-bourbonene | TRP58 | 5,36325 | Hydrophobic | Pi-Alkyl |
| 2QV4 | β-bourbonene | TRP58 | 5,42619 | Hydrophobic | Pi-Alkyl |
| 2QV4 | β-bourbonene | TRP59 | 4,80885 | Hydrophobic | Pi-Alkyl |
| 2QV4 | β-bourbonene | TRP59 | 4,98297 | Hydrophobic | Pi-Alkyl |
| 2QV4 | β-bourbonene | HIS299 | 4,86624 | Hydrophobic | Pi-Alkyl |
| 2QV4 | β-caryophyllene | TYR62 | 4,87087 | Hydrophobic | Pi-Alkyl |
| 2QV4 | β-copaene | TRP59 | 3,6466 | Hydrophobic | Pi-Sigma |
| 2QV4 | β-copaene | LEU165 | 5,26015 | Hydrophobic | Alkyl |
| 2QV4 | β-copaene | TRP58 | 4,82257 | Hydrophobic | Pi-Alkyl |
| 2QV4 | β-copaene | TYR62 | 4,09835 | Hydrophobic | Pi-Alkyl |
| 2QV4 | γ-muurolene | LEU165 | 4,76541 | Hydrophobic | Alkyl |
| 2QV4 | γ-muurolene | TRP59 | 4,96179 | Hydrophobic | Pi-Alkyl |
| 2QV4 | γ-muurolene | TRP59 | 4,60893 | Hydrophobic | Pi-Alkyl |
| 2QV4 | γ-muurolene | TYR62 | 5,18686 | Hydrophobic | Pi-Alkyl |
| 2QV4 | δ-cadinene | TRP59 | 3,85868 | Hydrophobic | Pi-Alkyl |
| 2QV4 | δ-cadinene | TRP59 | 5,10846 | Hydrophobic | Pi-Alkyl |
| 2QV4 | δ-cadinene | TRP59 | 4,24708 | Hydrophobic | Pi-Alkyl |
| 2QV4 | δ-cadinene | TRP59 | 3,9801 | Hydrophobic | Pi-Alkyl |
| 2QV4 | δ-cadinene | TRP59 | 4,79875 | Hydrophobic | Pi-Alkyl |
| 2QV4 | δ-cadinol | TRP59 | 2,9335 | Hydrogen Bond | Conventional Hydrogen Bond |
| 2QV4 | δ-cadinol | GLN63 | 3,26105 | Hydrogen Bond | Conventional Hydrogen Bond |
| 2QV4 | δ-cadinol | TRP59 | 3,57196 | Hydrophobic | Pi-Sigma |
| 2QV4 | δ-cadinol | TRP58 | 5,31144 | Hydrophobic | Pi-Alkyl |
| 2QV4 | δ-cadinol | TRP58 | 5,12505 | Hydrophobic | Pi-Alkyl |
| 2QV4 | δ-cadinol | TRP59 | 5,02289 | Hydrophobic | Pi-Alkyl |
| 2QV4 | δ-cadinol | TRP59 | 5,32598 | Hydrophobic | Pi-Alkyl |
| 2QV4 | δ-cadinol | TRP59 | 4,92973 | Hydrophobic | Pi-Alkyl |
| 3A4A | 1,8-cineol | TYR158 | 5,17212 | Hydrophobic | Pi-Alkyl |
| 3A4A | 1,8-cineol | PHE159 | 5,34645 | Hydrophobic | Pi-Alkyl |
| 3A4A | Bicyclogermacrene | TYR158 | 4,56673 | Hydrophobic | Pi-Alkyl |
| 3A4A | Borneol | PHE314 | 3,72807 | Hydrophobic | Pi-Sigma |
| 3A4A | Borneol | ILE419 | 5,49394 | Hydrophobic | Alkyl |
| 3A4A | Borneol | PHE314 | 4,33123 | Hydrophobic | Pi-Alkyl |
| 3A4A | Calamenene | TYR158 | 5,02632 | Hydrophobic | Pi-Alkyl |
| 3A4A | Calamenene | HIS280 | 4,80164 | Hydrophobic | Pi-Alkyl |
| 3A4A | Camphene | ILE419 | 5,3322 | Hydrophobic | Alkyl |
| 3A4A | Camphene | ILE419 | 5,40853 | Hydrophobic | Alkyl |
| 3A4A | Camphene | PHE314 | 4,12487 | Hydrophobic | Pi-Alkyl |
| 3A4A | Camphene | HIS423 | 5,43571 | Hydrophobic | Pi-Alkyl |
| 3A4A | Camphene | HIS423 | 4,56885 | Hydrophobic | Pi-Alkyl |
| 3A4A | Camphor | PHE314 | 3,83519 | Hydrophobic | Pi-Sigma |
| 3A4A | Camphor | LYS156 | 5,33425 | Hydrophobic | Alkyl |
| 3A4A | Camphor | ALA418 | 4,6094 | Hydrophobic | Alkyl |
| 3A4A | Camphor | PHE314 | 4,73276 | Hydrophobic | Pi-Alkyl |
| 3A4A | Carvone | PHE314 | 3,44696 | Hydrophobic | Pi-Sigma |
| 3A4A | Carvone | ILE419 | 4,90783 | Hydrophobic | Alkyl |
| 3A4A | Carvone | ALA418 | 4,84171 | Hydrophobic | Alkyl |
| 3A4A | Caryophyllene Oxide | ARG442 | 2,8248 | Hydrogen Bond | Conventional Hydrogen Bond |
| 3A4A | Caryophyllene Oxide | ARG442 | 3,48629 | Hydrogen Bond | Carbon Hydrogen Bond |
| 3A4A | Caryophyllene Oxide | PHE303 | 5,43367 | Hydrophobic | Pi-Alkyl |
| 3A4A | Epi-Cubebol | ASP352 | 2,91959 | Hydrogen Bond | Conventional Hydrogen Bond |
| 3A4A | Epi-Cubebol | GLN353 | 2,80969 | Hydrogen Bond | Conventional Hydrogen Bond |
| 3A4A | Epi-Cubebol | TYR158 | 5,27665 | Hydrophobic | Pi-Alkyl |
| 3A4A | Germacrene-D-4-ol | ARG315 | 5,37632 | Hydrophobic | Alkyl |
| 3A4A | Germacrene-D-4-ol | PHE303 | 5,33962 | Hydrophobic | Pi-Alkyl |
| 3A4A | Germacrene-D-4-ol | PHE303 | 4,26528 | Hydrophobic | Pi-Alkyl |
| 3A4A | Hexahydrofarnesylacetone | THR310 | 3,2678 | Hydrogen Bond | Conventional Hydrogen Bond |
| 3A4A | Hexahydrofarnesylacetone | TYR72 | 3,99937 | Hydrophobic | Pi-Sigma |
| 3A4A | Hexahydrofarnesylacetone | TYR72 | 4,36243 | Hydrophobic | Pi-Alkyl |
| 3A4A | Hexahydrofarnesylacetone | HIS112 | 4,67239 | Hydrophobic | Pi-Alkyl |
| 3A4A | Hexahydrofarnesylacetone | TYR158 | 4,97629 | Hydrophobic | Pi-Alkyl |
| 3A4A | Hexahydrofarnesylacetone | PHE178 | 4,68072 | Hydrophobic | Pi-Alkyl |
| 3A4A | Hexahydrofarnesylacetone | HIS280 | 4,90522 | Hydrophobic | Pi-Alkyl |
| 3A4A | Ledol | GLN353 | 2,6999 | Hydrogen Bond | Conventional Hydrogen Bond |
| 3A4A | Ledol | PHE303 | 3,93663 | Hydrophobic | Pi-Sigma |
| 3A4A | Ledol | PHE303 | 5,12836 | Hydrophobic | Pi-Alkyl |
| 3A4A | Limonene | ILE419 | 5,30644 | Hydrophobic | Alkyl |
| 3A4A | Limonene | PHE314 | 4,11478 | Hydrophobic | Pi-Alkyl |
| 3A4A | Limonene | PHE314 | 5,49393 | Hydrophobic | Pi-Alkyl |
| 3A4A | Limonene | HIS423 | 5,35364 | Hydrophobic | Pi-Alkyl |
| 3A4A | Linalool | GLU277 | 3,09328 | Hydrogen Bond | Conventional Hydrogen Bond |
| 3A4A | Linalool | ASP352 | 3,12121 | Hydrogen Bond | Conventional Hydrogen Bond |
| 3A4A | Linalool | PHE178 | 3,84011 | Hydrophobic | Pi-Sigma |
| 3A4A | Linalool | TYR72 | 3,94486 | Hydrophobic | Pi-Sigma |
| 3A4A | Linalool | VAL216 | 5,34465 | Hydrophobic | Alkyl |
| 3A4A | Linalool | HIS112 | 4,69506 | Hydrophobic | Pi-Alkyl |
| 3A4A | Linalool | PHE178 | 4,91599 | Hydrophobic | Pi-Alkyl |
| 3A4A | Myrcene | ILE419 | 5,48051 | Hydrophobic | Alkyl |
| 3A4A | Myrcene | ILE419 | 5,02649 | Hydrophobic | Alkyl |
| 3A4A | Myrcene | LYS156 | 4,41807 | Hydrophobic | Alkyl |
| 3A4A | Myrcene | ALA418 | 4,66129 | Hydrophobic | Alkyl |
| 3A4A | Myrcene | PHE314 | 4,30092 | Hydrophobic | Pi-Alkyl |
| 3A4A | Myrcene | PHE314 | 4,18755 | Hydrophobic | Pi-Alkyl |
| 3A4A | Myrcene | HIS423 | 5,26009 | Hydrophobic | Pi-Alkyl |
| 3A4A | Myrtenal | PHE314 | 3,73533 | Hydrophobic | Pi-Sigma |
| 3A4A | Myrtenal | PHE314 | 4,68818 | Hydrophobic | Pi-Alkyl |
| 3A4A | Myrtenal | HIS423 | 5,36331 | Hydrophobic | Pi-Alkyl |
| 3A4A | Myrtenol | PHE314 | 3,73171 | Hydrophobic | Pi-Sigma |
| 3A4A | Myrtenol | ILE419 | 5,19972 | Hydrophobic | Alkyl |
| 3A4A | Myrtenol | HIS423 | 5,0932 | Hydrophobic | Pi-Alkyl |
| 3A4A | p-Cymene | PHE314 | 3,94442 | Hydrophobic | Pi-Pi Stacked |
| 3A4A | p-Cymene | ILE419 | 5,48639 | Hydrophobic | Pi-Alkyl |
| 3A4A | p-Cymene | PHE314 | 5,43572 | Hydrophobic | Pi-Alkyl |
| 3A4A | Pulegone | PHE314 | 3,45256 | Hydrophobic | Pi-Sigma |
| 3A4A | Pulegone | PHE314 | 4,17948 | Hydrophobic | Pi-Alkyl |
| 3A4A | Sabinene | ALA418 | 4,76311 | Hydrophobic | Alkyl |
| 3A4A | Sabinene | ALA418 | 4,10747 | Hydrophobic | Alkyl |
| 3A4A | Sabinene | ILE419 | 5,24401 | Hydrophobic | Alkyl |
| 3A4A | Sabinene | LYS156 | 4,34512 | Hydrophobic | Alkyl |
| 3A4A | Sabinene | TRP238 | 5,38782 | Hydrophobic | Pi-Alkyl |
| 3A4A | Sabinene | PHE314 | 4,7916 | Hydrophobic | Pi-Alkyl |
| 3A4A | Spathulenol | PHE303 | 4,6525 | Hydrophobic | Pi-Alkyl |
| 3A4A | T-Cadinol | GLU411 | 3,03067 | Hydrogen Bond | Conventional Hydrogen Bond |
| 3A4A | T-Cadinol | ARG442 | 3,05433 | Hydrogen Bond | Conventional Hydrogen Bond |
| 3A4A | T-Cadinol | ARG442 | 3,50519 | Hydrogen Bond | Carbon Hydrogen Bond |
| 3A4A | T-Cadinol | ARG315 | 4,08383 | Hydrophobic | Alkyl |
| 3A4A | T-Cadinol | ARG315 | 5,26142 | Hydrophobic | Alkyl |
| 3A4A | T-Cadinol | TYR158 | 5,43548 | Hydrophobic | Pi-Alkyl |
| 3A4A | T-Cadinol | PHE303 | 4,3726 | Hydrophobic | Pi-Alkyl |
| 3A4A | Terpinen-4-ol | SER236 | 3,17816 | Hydrogen Bond | Conventional Hydrogen Bond |
| 3A4A | Terpinen-4-ol | PHE314 | 3,64028 | Hydrophobic | Pi-Sigma |
| 3A4A | Terpinen-4-ol | ILE419 | 5,44194 | Hydrophobic | Alkyl |
| 3A4A | Terpinen-4-ol | HIS423 | 5,33497 | Hydrophobic | Pi-Alkyl |
| 3A4A | Terpinen-4-ol | HIS423 | 5,03386 | Hydrophobic | Pi-Alkyl |
| 3A4A | Tricosane | TRP36 | 5,02269 | Hydrophobic | Pi-Alkyl |
| 3A4A | Tricosane | TRP81 | 5,00672 | Hydrophobic | Pi-Alkyl |
| 3A4A | Tricosane | TRP81 | 5,0551 | Hydrophobic | Pi-Alkyl |
| 3A4A | Tricosane | TRP81 | 4,96665 | Hydrophobic | Pi-Alkyl |
| 3A4A | Tricosane | PHE469 | 5,00125 | Hydrophobic | Pi-Alkyl |
| 3A4A | Tricosane | TYR470 | 4,79317 | Hydrophobic | Pi-Alkyl |
| 3A4A | Viridiflorol | ARG315 | 4,58717 | Hydrophobic | Alkyl |
| 3A4A | Viridiflorol | YR158 | 5,01645 | Hydrophobic | Pi-Alkyl |
| 3A4A | α-cadinol | TYR158 | 2,91952 | Hydrogen Bond | Conventional Hydrogen Bond |
| 3A4A | α-cadinol | LYS156 | 5,25376 | Hydrophobic | Alkyl |
| 3A4A | α-cadinol | TYR158 | 4,22771 | Hydrophobic | Pi-Alkyl |
| 3A4A | α-cadinol | TYR158 | 5,20783 | Hydrophobic | Pi-Alkyl |
| 3A4A | α-cadinol | TYR158 | 4,05724 | Hydrophobic | Pi-Alkyl |
| 3A4A | α-copaene | VAL216 | 5,29525 | Hydrophobic | Alkyl |
| 3A4A | α-copaene | PHE303 | 5,25335 | Hydrophobic | Pi-Alkyl |
| 3A4A | α-copaene | PHE303 | 5,33219 | Hydrophobic | Pi-Alkyl |
| 3A4A | α-cubebene | ARG315 | 4,64396 | Hydrophobic | Alkyl |
| 3A4A | α-cubebene | ARG315 | 4,56731 | Hydrophobic | Alkyl |
| 3A4A | α-cubebene | PHE303 | 5,11407 | Hydrophobic | Pi-Alkyl |
| 3A4A | α-humulene | PHE303 | 3,85457 | Hydrophobic | Pi-Sigma |
| 3A4A | α-humulene | HIS280 | 4,82687 | Hydrophobic | Pi-Alkyl |
| 3A4A | α-humulene | PHE303 | 5,05199 | Hydrophobic | Pi-Alkyl |
| 3A4A | α-muurolene | LYS156 | 4,41476 | Hydrophobic | Alkyl |
| 3A4A | α-muurolene | ARG315 | 4,12047 | Hydrophobic | Alkyl |
| 3A4A | α-muurolene | TYR158 | 4,03832 | Hydrophobic | Pi-Alkyl |
| 3A4A | α-muurolene | TYR158 | 4,19921 | Hydrophobic | Pi-Alkyl |
| 3A4A | α-muurolene | PHE314 | 4,7826 | Hydrophobic | Pi-Alkyl |
| 3A4A | α-muurolene | TYR316 | 5,16245 | Hydrophobic | Pi-Alkyl |
| 3A4A | α-phellandrene | ILE419 | 5,18682 | Hydrophobic | Alkyl |
| 3A4A | α-phellandrene | PHE314 | 4,09602 | Hydrophobic | Pi-Alkyl |
| 3A4A | α-phellandrene | PHE314 | 5,17972 | Hydrophobic | Pi-Alkyl |
| 3A4A | α-phellandrene | HIS423 | 5,34549 | Hydrophobic | Pi-Alkyl |
| 3A4A | α-terpineol | SER236 | 2,9695 | Hydrogen Bond | Conventional Hydrogen Bond |
| 3A4A | α-terpineol | GLU422 | 3,206 | Hydrogen Bond | Conventional Hydrogen Bond |
| 3A4A | α-terpineol | GLU422 | 3,14715 | Hydrogen Bond | Conventional Hydrogen Bond |
| 3A4A | α-terpineol | PHE314 | 4,77632 | Hydrophobic | Pi-Alkyl |
| 3A4A | α-terpineol | HIS423 | 4,83153 | Hydrophobic | Pi-Alkyl |
| 3A4A | α-thujene | LYS156 | 4,21058 | Hydrophobic | Alkyl |
| 3A4A | α-thujene | ALA418 | 4,20112 | Hydrophobic | Alkyl |
| 3A4A | α-thujene | ALA418 | 4,58502 | Hydrophobic | Alkyl |
| 3A4A | α-thujene | ILE419 | 5,12722 | Hydrophobic | Alkyl |
| 3A4A | α-thujene | TRP238 | 5,28021 | Hydrophobic | Pi-Alkyl |
| 3A4A | α-thujene | PHE314 | 4,86808 | Hydrophobic | Pi-Alkyl |
| 3A4A | β-bourbonene | TYR158 | 5,40066 | Hydrophobic | Pi-Alkyl |
| 3A4A | β-bourbonene | PHE303 | 4,85903 | Hydrophobic | Pi-Alkyl |
| 3A4A | β-caryophyllene | PHE303 | 5,46425 | Hydrophobic | Pi-Alkyl |
| 3A4A | β-copaene | VAL216 | 4,84331 | Hydrophobic | Alkyl |
| 3A4A | β-copaene | TYR158 | 5,01191 | Hydrophobic | Pi-Alkyl |
| 3A4A | β-copaene | TYR158 | 5,26481 | Hydrophobic | Pi-Alkyl |
| 3A4A | β-copaene | PHE178 | 5,04683 | Hydrophobic | Pi-Alkyl |
| 3A4A | β-copaene | PHE303 | 4,89711 | Hydrophobic | Pi-Alkyl |
| 3A4A | γ-muurolene | TYR158 | 4,97578 | Hydrophobic | Pi-Alkyl |
| 3A4A | γ-muurolene | PHE303 | 5,40488 | Hydrophobic | Pi-Alkyl |
| 3A4A | γ-muurolene | PHE303 | 4,21469 | Hydrophobic | Pi-Alkyl |
| 3A4A | δ-cadinene | TYR158 | 3,88077 | Hydrophobic | Pi-Sigma |
| 3A4A | δ-cadinene | ARG315 | 5,21158 | Hydrophobic | Alkyl |
| 3A4A | δ-cadinene | TYR158 | 4,96347 | Hydrophobic | Pi-Alkyl |
| 3A4A | δ-cadinene | TYR158 | 5,16242 | Hydrophobic | Pi-Alkyl |
| 3A4A | δ-cadinene | HIS280 | 5,06545 | Hydrophobic | Pi-Alkyl |
| 3A4A | δ-cadinol | ARG315 | 4,18165 | Hydrophobic | Alkyl |
| 3A4A | δ-cadinol | ARG315 | 5,33744 | Hydrophobic | Alkyl |
| 3A4A | δ-cadinol | PHE314 | 4,83758 | Hydrophobic | Pi-Alkyl |
| 3A4A | δ-cadinol | TYR316 | 5,02509 | Hydrophobic | Pi-Alkyl |
| 4EY7 | 1,8-cineol | TYR124 | 3,21454 | Hydrogen Bond | Conventional Hydrogen Bond |
| 4EY7 | 1,8-cineol | TYR337 | 3,5749 | Hydrophobic | Pi-Sigma |
| 4EY7 | 1,8-cineol | TYR341 | 3,67951 | Hydrophobic | Pi-Sigma |
| 4EY7 | 1,8-cineol | TYR337 | 5,35905 | Hydrophobic | Pi-Alkyl |
| 4EY7 | 1,8-cineol | PHE338 | 5,00577 | Hydrophobic | Pi-Alkyl |
| 4EY7 | 1,8-cineol | PHE338 | 3,9527 | Hydrophobic | Pi-Alkyl |
| 4EY7 | 1,8-cineol | TYR341 | 4,84702 | Hydrophobic | Pi-Alkyl |
| 4EY7 | Bicyclogermacrene | PHE338 | 3,81431 | Hydrophobic | Pi-Sigma |
| 4EY7 | Bicyclogermacrene | TYR337 | 3,87558 | Hydrophobic | Pi-Sigma |
| 4EY7 | Bicyclogermacrene | TYR337 | 5,43123 | Hydrophobic | Pi-Alkyl |
| 4EY7 | Borneol | GLU202 | 3,19755 | Hydrogen Bond | Conventional Hydrogen Bond |
| 4EY7 | Borneol | SER203 | 2,99981 | Hydrogen Bond | Conventional Hydrogen Bond |
| 4EY7 | Borneol | TRP86 | 3,63661 | Hydrophobic | Pi-Sigma |
| 4EY7 | Borneol | TRP86 | 3,80255 | Hydrophobic | Pi-Sigma |
| 4EY7 | Borneol | TRP86 | 4,66105 | Hydrophobic | Pi-Alkyl |
| 4EY7 | Borneol | TRP86 | 4,77222 | Hydrophobic | Pi-Alkyl |
| 4EY7 | Bornyl Acetate | TYR337 | 3,78543 | Hydrophobic | Pi-Sigma |
| 4EY7 | Bornyl Acetate | TRP86 | 4,88186 | Hydrophobic | Pi-Alkyl |
| 4EY7 | Bornyl Acetate | TYR337 | 4,90331 | Hydrophobic | Pi-Alkyl |
| 4EY7 | Calamenene | TRP86 | 5,12732 | Hydrophobic | Pi-Alkyl |
| 4EY7 | Calamenene | TRP86 | 5,38619 | Hydrophobic | Pi-Alkyl |
| 4EY7 | Calamenene | PHE295 | 4,54226 | Hydrophobic | Pi-Alkyl |
| 4EY7 | Calamenene | PHE297 | 4,70618 | Hydrophobic | Pi-Alkyl |
| 4EY7 | Calamenene | PHE338 | 5,21151 | Hydrophobic | Pi-Alkyl |
| 4EY7 | Calamenene | HIS447 | 4,6993 | Hydrophobic | Pi-Alkyl |
| 4EY7 | Camphene | TRP86 | 3,54089 | Hydrophobic | Pi-Sigma |
| 4EY7 | Camphene | TYR337 | 3,65051 | Hydrophobic | Pi-Sigma |
| 4EY7 | Camphene | TRP86 | 4,93661 | Hydrophobic | Pi-Alkyl |
| 4EY7 | Camphene | TYR337 | 5,13696 | Hydrophobic | Pi-Alkyl |
| 4EY7 | Camphene | TYR337 | 4,22335 | Hydrophobic | Pi-Alkyl |
| 4EY7 | Camphene | PHE338 | 4,14697 | Hydrophobic | Pi-Alkyl |
| 4EY7 | Camphene | PHE338 | 4,95223 | Hydrophobic | Pi-Alkyl |
| 4EY7 | Camphene | TYR341 | 4,24962 | Hydrophobic | Pi-Alkyl |
| 4EY7 | Camphene | HIS447 | 4,8315 | Hydrophobic | Pi-Alkyl |
| 4EY7 | Camphor | TYR124 | 2,90945 | Hydrogen Bond | Conventional Hydrogen Bond |
| 4EY7 | Camphor | TYR337 | 4,30113 | Hydrophobic | Pi-Alkyl |
| 4EY7 | Camphor | PHE338 | 4,66761 | Hydrophobic | Pi-Alkyl |
| 4EY7 | Camphor | HIS447 | 5,43536 | Hydrophobic | Pi-Alkyl |
| 4EY7 | Carvone | TYR124 | 3,1176 | Hydrogen Bond | Conventional Hydrogen Bond |
| 4EY7 | Carvone | TYR337 | 3,62911 | Hydrophobic | Pi-Sigma |
| 4EY7 | Carvone | TYR341 | 3,88568 | Hydrophobic | Pi-Alkyl |
| 4EY7 | Caryophyllene Oxide | TYR124 | 2,84522 | Hydrogen Bond | Conventional Hydrogen Bond |
| 4EY7 | Caryophyllene Oxide | TRP86 | 4,3398 | Hydrophobic | Pi-Alkyl |
| 4EY7 | Caryophyllene Oxide | TRP86 | 5,31421 | Hydrophobic | Pi-Alkyl |
| 4EY7 | Epi-Cubebol | SER203 | 2,99562 | Hydrogen Bond | Conventional Hydrogen Bond |
| 4EY7 | Epi-Cubebol | TYR341 | 3,79233 | Hydrophobic | Pi-Sigma |
| 4EY7 | Epi-Cubebol | PHE338 | 4,45317 | Hydrophobic | Pi-Alkyl |
| 4EY7 | Epi-Cubebol | TYR341 | 5,14499 | Hydrophobic | Pi-Alkyl |
| 4EY7 | Germacrene-D-4-ol | TYR337 | 3,59509 | Hydrophobic | Pi-Sigma |
| 4EY7 | Germacrene-D-4-ol | PHE338 | 3,81195 | Hydrophobic | Pi-Sigma |
| 4EY7 | Germacrene-D-4-ol | TRP86 | 4,81929 | Hydrophobic | Pi-Alkyl |
| 4EY7 | Hexahydrofarnesylacetone | TYR337 | 3,42077 | Hydrophobic | Pi-Sigma |
| 4EY7 | Hexahydrofarnesylacetone | TYR337 | 3,99961 | Hydrophobic | Pi-Sigma |
| 4EY7 | Hexahydrofarnesylacetone | TRP286 | 5,06605 | Hydrophobic | Pi-Alkyl |
| 4EY7 | Hexahydrofarnesylacetone | TRP286 | 4,98255 | Hydrophobic | Pi-Alkyl |
| 4EY7 | Hexahydrofarnesylacetone | PHE338 | 4,40045 | Hydrophobic | Pi-Alkyl |
| 4EY7 | Hexahydrofarnesylacetone | TYR341 | 4,3661 | Hydrophobic | Pi-Alkyl |
| 4EY7 | Hexahydrofarnesylacetone | HIS447 | 5,15387 | Hydrophobic | Pi-Alkyl |
| 4EY7 | Ledol | GLU202 | 2,42412 | Hydrogen Bond | Conventional Hydrogen Bond |
| 4EY7 | Ledol | SER203 | 2,95131 | Hydrogen Bond | Conventional Hydrogen Bond |
| 4EY7 | Ledol | TRP86 | 3,53401 | Hydrophobic | Pi-Sigma |
| 4EY7 | Ledol | TRP86 | 5,15835 | Hydrophobic | Pi-Alkyl |
| 4EY7 | Ledol | PHE297 | 5,30528 | Hydrophobic | Pi-Alkyl |
| 4EY7 | Ledol | PHE338 | 5,38009 | Hydrophobic | Pi-Alkyl |
| 4EY7 | Ledol | HIS447 | 5,38022 | Hydrophobic | Pi-Alkyl |
| 4EY7 | Limonene | PHE338 | 3,70981 | Hydrophobic | Pi-Sigma |
| 4EY7 | Limonene | TRP86 | 4,08002 | Hydrophobic | Pi-Alkyl |
| 4EY7 | Limonene | TRP86 | 4,31534 | Hydrophobic | Pi-Alkyl |
| 4EY7 | Limonene | TYR337 | 3,94496 | Hydrophobic | Pi-Alkyl |
| 4EY7 | Limonene | TYR337 | 4,13321 | Hydrophobic | Pi-Alkyl |
| 4EY7 | Limonene | PHE338 | 5,063 | Hydrophobic | Pi-Alkyl |
| 4EY7 | Limonene | TYR341 | 4,1231 | Hydrophobic | Pi-Alkyl |
| 4EY7 | Myrcene | TYR337 | 3,74217 | Hydrophobic | Pi-Sigma |
| 4EY7 | Myrcene | PHE338 | 3,66858 | Hydrophobic | Pi-Sigma |
| 4EY7 | Myrcene | TRP286 | 4,77326 | Hydrophobic | Pi-Alkyl |
| 4EY7 | Myrcene | TYR337 | 4,1055 | Hydrophobic | Pi-Alkyl |
| 4EY7 | Myrcene | PHE338 | 4,93299 | Hydrophobic | Pi-Alkyl |
| 4EY7 | Myrcene | TYR341 | 3,68479 | Hydrophobic | Pi-Alkyl |
| 4EY7 | Myrcene | TYR341 | 5,22786 | Hydrophobic | Pi-Alkyl |
| 4EY7 | Myrtenal | SER203 | 2,87893 | Hydrogen Bond | Conventional Hydrogen Bond |
| 4EY7 | Myrtenal | TYR337 | 3,56575 | Hydrophobic | Pi-Sigma |
| 4EY7 | Myrtenal | TYR337 | 4,6861 | Hydrophobic | Pi-Alkyl |
| 4EY7 | Myrtenol | SER203 | 2,92595 | Hydrogen Bond | Conventional Hydrogen Bond |
| 4EY7 | Myrtenol | GLY121 | 3,19884 | Hydrogen Bond | Conventional Hydrogen Bond |
| 4EY7 | Myrtenol | TYR337 | 3,55111 | Hydrophobic | Pi-Sigma |
| 4EY7 | Myrtenol | TYR337 | 4,68145 | Hydrophobic | Pi-Alkyl |
| 4EY7 | Myrtenol | PHE338 | 5,49195 | Hydrophobic | Pi-Alkyl |
| 4EY7 | p-Cymene | TYR341 | 3,8297 | Hydrophobic | Pi-Sigma |
| 4EY7 | p-Cymene | TRP286 | 3,84451 | Hydrophobic | Pi-Sigma |
| 4EY7 | p-Cymene | TRP286 | 3,88779 | Hydrophobic | Pi-Pi Stacked |
| 4EY7 | p-Cymene | TYR341 | 4,57594 | Hydrophobic | Pi-Pi Stacked |
| 4EY7 | p-Cymene | TYR72 | 4,5074 | Hydrophobic | Pi-Alkyl |
| 4EY7 | p-Cymene | TRP286 | 4,03301 | Hydrophobic | Pi-Alkyl |
| 4EY7 | Pinocarvone | TYR337 | 3,58912 | Hydrophobic | Pi-Sigma |
| 4EY7 | Pinocarvone | PHE338 | 3,96926 | Hydrophobic | Pi-Sigma |
| 4EY7 | Pinocarvone | PHE338 | 3,60447 | Hydrophobic | Pi-Sigma |
| 4EY7 | Pulegone | TYR124 | 2,77873 | Hydrogen Bond | Conventional Hydrogen Bond |
| 4EY7 | Pulegone | PHE338 | 3,92539 | Hydrophobic | Pi-Sigma |
| 4EY7 | Pulegone | TRP286 | 4,72227 | Hydrophobic | Pi-Alkyl |
| 4EY7 | Sabinene | TYR341 | 3,63401 | Hydrophobic | Pi-Sigma |
| 4EY7 | Sabinene | TRP86 | 3,70701 | Hydrophobic | Pi-Alkyl |
| 4EY7 | Sabinene | TRP86 | 4,06869 | Hydrophobic | Pi-Alkyl |
| 4EY7 | Sabinene | TYR337 | 3,92635 | Hydrophobic | Pi-Alkyl |
| 4EY7 | Sabinene | TYR337 | 4,26689 | Hydrophobic | Pi-Alkyl |
| 4EY7 | Sabinene | PHE338 | 4,90046 | Hydrophobic | Pi-Alkyl |
| 4EY7 | Spathulenol | PHE338 | 3,9386 | Hydrophobic | Pi-Sigma |
| 4EY7 | Spathulenol | TRP86 | 5,18 | Hydrophobic | Pi-Alkyl |
| 4EY7 | Spathulenol | TYR124 | 5,27428 | Hydrophobic | Pi-Alkyl |
| 4EY7 | Spathulenol | PHE297 | 5,02424 | Hydrophobic | Pi-Alkyl |
| 4EY7 | Spathulenol | PHE338 | 4,63362 | Hydrophobic | Pi-Alkyl |
| 4EY7 | T-Cadinol | TRP86 | 4,74633 | Hydrophobic | Pi-Alkyl |
| 4EY7 | T-Cadinol | TRP86 | 5,47556 | Hydrophobic | Pi-Alkyl |
| 4EY7 | T-Cadinol | PHE297 | 4,85559 | Hydrophobic | Pi-Alkyl |
| 4EY7 | T-Cadinol | TYR337 | 5,14343 | Hydrophobic | Pi-Alkyl |
| 4EY7 | T-Cadinol | PHE338 | 4,63838 | Hydrophobic | Pi-Alkyl |
| 4EY7 | T-Cadinol | PHE338 | 5,46824 | Hydrophobic | Pi-Alkyl |
| 4EY7 | T-Cadinol | HIS447 | 5,4606 | Hydrophobic | Pi-Alkyl |
| 4EY7 | T-Cadinol | HIS447 | 4,92748 | Hydrophobic | Pi-Alkyl |
| 4EY7 | Terpinen-4-ol | HIS447 | 3,67023 | Hydrogen Bond | Carbon Hydrogen Bond |
| 4EY7 | Terpinen-4-ol | TRP86 | 3,47624 | Hydrophobic | Pi-Sigma |
| 4EY7 | Terpinen-4-ol | PHE338 | 3,72664 | Hydrophobic | Pi-Sigma |
| 4EY7 | Terpinen-4-ol | TYR337 | 3,96025 | Hydrophobic | Pi-Alkyl |
| 4EY7 | Terpinen-4-ol | TYR337 | 4,20113 | Hydrophobic | Pi-Alkyl |
| 4EY7 | Terpinen-4-ol | PHE338 | 4,76431 | Hydrophobic | Pi-Alkyl |
| 4EY7 | Terpinen-4-ol | TYR341 | 4,11749 | Hydrophobic | Pi-Alkyl |
| 4EY7 | Tricosane | TRP86 | 3,6224 | Hydrophobic | Pi-Sigma |
| 4EY7 | Tricosane | LEU289 | 5,20533 | Hydrophobic | Alkyl |
| 4EY7 | Tricosane | TRP86 | 4,99173 | Hydrophobic | Pi-Alkyl |
| 4EY7 | Tricosane | TRP86 | 4,23905 | Hydrophobic | Pi-Alkyl |
| 4EY7 | Tricosane | TRP86 | 5,40069 | Hydrophobic | Pi-Alkyl |
| 4EY7 | Tricosane | TRP286 | 4,44077 | Hydrophobic | Pi-Alkyl |
| 4EY7 | Tricosane | TRP286 | 5,00606 | Hydrophobic | Pi-Alkyl |
| 4EY7 | Tricosane | TYR337 | 4,25219 | Hydrophobic | Pi-Alkyl |
| 4EY7 | Tricosane | TYR337 | 5,22395 | Hydrophobic | Pi-Alkyl |
| 4EY7 | Tricosane | PHE338 | 4,38305 | Hydrophobic | Pi-Alkyl |
| 4EY7 | Tricosane | TYR341 | 4,73733 | Hydrophobic | Pi-Alkyl |
| 4EY7 | Tricosane | TYR341 | 5,03554 | Hydrophobic | Pi-Alkyl |
| 4EY7 | Tricosane | HIS447 | 5,32561 | Hydrophobic | Pi-Alkyl |
| 4EY7 | Tricosane | HIS447 | 5,01912 | Hydrophobic | Pi-Alkyl |
| 4EY7 | Viridiflorol | HIS447 | 3,55677 | Hydrogen Bond | Carbon Hydrogen Bond |
| 4EY7 | Viridiflorol | TYR337 | 3,28388 | Hydrogen Bond | Pi-Donor Hydrogen Bond |
| 4EY7 | Viridiflorol | TRP86 | 3,88864 | Hydrophobic | Pi-Sigma |
| 4EY7 | Viridiflorol | TYR337 | 3,81192 | Hydrophobic | Pi-Sigma |
| 4EY7 | Viridiflorol | PHE338 | 3,83765 | Hydrophobic | Pi-Sigma |
| 4EY7 | Viridiflorol | TRP86 | 5,0291 | Hydrophobic | Pi-Alkyl |
| 4EY7 | Viridiflorol | PHE297 | 5,23411 | Hydrophobic | Pi-Alkyl |
| 4EY7 | Viridiflorol | TYR337 | 5,05821 | Hydrophobic | Pi-Alkyl |
| 4EY7 | Viridiflorol | PHE338 | 4,8284 | Hydrophobic | Pi-Alkyl |
| 4EY7 | α-cadinol | TYR124 | 3,07604 | Hydrogen Bond | Conventional Hydrogen Bond |
| 4EY7 | α-cadinol | TRP86 | 3,80785 | Hydrophobic | Pi-Sigma |
| 4EY7 | α-cadinol | TYR337 | 3,95353 | Hydrophobic | Pi-Sigma |
| 4EY7 | α-cadinol | TRP86 | 3,96633 | Hydrophobic | Pi-Sigma |
| 4EY7 | α-cadinol | TRP86 | 3,75668 | Hydrophobic | Pi-Sigma |
| 4EY7 | α-cadinol | PHE295 | 5,19084 | Hydrophobic | Pi-Alkyl |
| 4EY7 | α-cadinol | PHE297 | 5,32392 | Hydrophobic | Pi-Alkyl |
| 4EY7 | α-cadinol | PHE297 | 4,91929 | Hydrophobic | Pi-Alkyl |
| 4EY7 | α-cadinol | TYR337 | 5,31447 | Hydrophobic | Pi-Alkyl |
| 4EY7 | α-copaene | TYR337 | 3,69661 | Hydrophobic | Pi-Sigma |
| 4EY7 | α-copaene | TYR124 | 5,40032 | Hydrophobic | Pi-Alkyl |
| 4EY7 | α-copaene | PHE297 | 5,09172 | Hydrophobic | Pi-Alkyl |
| 4EY7 | α-copaene | TYR337 | 4,92892 | Hydrophobic | Pi-Alkyl |
| 4EY7 | α-copaene | PHE338 | 4,42642 | Hydrophobic | Pi-Alkyl |
| 4EY7 | α-copaene | TYR341 | 4,21055 | Hydrophobic | Pi-Alkyl |
| 4EY7 | α-cubebene | TRP86 | 3,69292 | Hydrophobic | Pi-Sigma |
| 4EY7 | α-cubebene | PHE338 | 3,83586 | Hydrophobic | Pi-Sigma |
| 4EY7 | α-cubebene | TYR337 | 3,45671 | Hydrophobic | Pi-Sigma |
| 4EY7 | α-cubebene | TRP86 | 4,24866 | Hydrophobic | Pi-Alkyl |
| 4EY7 | α-cubebene | TRP86 | 4,9669 | Hydrophobic | Pi-Alkyl |
| 4EY7 | α-cubebene | TRP86 | 4,62568 | Hydrophobic | Pi-Alkyl |
| 4EY7 | α-cubebene | TRP86 | 4,46178 | Hydrophobic | Pi-Alkyl |
| 4EY7 | α-cubebene | TYR337 | 5,27444 | Hydrophobic | Pi-Alkyl |
| 4EY7 | α-cubebene | HIS447 | 5,35091 | Hydrophobic | Pi-Alkyl |
| 4EY7 | α-humulene | TYR72 | 5,03587 | Hydrophobic | Pi-Alkyl |
| 4EY7 | α-humulene | TYR124 | 4,7527 | Hydrophobic | Pi-Alkyl |
| 4EY7 | α-humulene | TRP286 | 4,90377 | Hydrophobic | Pi-Alkyl |
| 4EY7 | α-humulene | TRP286 | 3,79377 | Hydrophobic | Pi-Alkyl |
| 4EY7 | α-humulene | TRP286 | 4,62185 | Hydrophobic | Pi-Alkyl |
| 4EY7 | α-humulene | TRP286 | 4,87382 | Hydrophobic | Pi-Alkyl |
| 4EY7 | α-humulene | TYR341 | 5,34561 | Hydrophobic | Pi-Alkyl |
| 4EY7 | α-humulene | TYR341 | 4,75711 | Hydrophobic | Pi-Alkyl |
| 4EY7 | α-muurolene | TYR341 | 3,76162 | Hydrophobic | Pi-Sigma |
| 4EY7 | α-muurolene | TYR341 | 3,9573 | Hydrophobic | Pi-Sigma |
| 4EY7 | α-muurolene | TRP86 | 3,50465 | Hydrophobic | Pi-Sigma |
| 4EY7 | α-muurolene | TRP86 | 4,38644 | Hydrophobic | Pi-Alkyl |
| 4EY7 | α-muurolene | TRP86 | 5,38933 | Hydrophobic | Pi-Alkyl |
| 4EY7 | α-muurolene | TYR337 | 5,40482 | Hydrophobic | Pi-Alkyl |
| 4EY7 | α-muurolene | PHE338 | 4,56166 | Hydrophobic | Pi-Alkyl |
| 4EY7 | α-phellandrene | TYR341 | 3,50546 | Hydrophobic | Pi-Sigma |
| 4EY7 | α-phellandrene | VAL294 | 5,01255 | Hydrophobic | Alkyl |
| 4EY7 | α-phellandrene | TRP286 | 5,47495 | Hydrophobic | Pi-Alkyl |
| 4EY7 | α-phellandrene | TRP286 | 5,01976 | Hydrophobic | Pi-Alkyl |
| 4EY7 | α-terpineol | TYR124 | 3,01666 | Hydrogen Bond | Conventional Hydrogen Bond |
| 4EY7 | α-terpineol | TYR341 | 4,01932 | Hydrogen Bond | Pi-Donor Hydrogen Bond |
| 4EY7 | α-terpineol | TYR341 | 3,5611 | Hydrophobic | Pi-Sigma |
| 4EY7 | α-terpineol | TYR341 | 3,96424 | Hydrophobic | Pi-Sigma |
| 4EY7 | α-terpineol | VAL294 | 4,86847 | Hydrophobic | Alkyl |
| 4EY7 | α-terpineol | TRP286 | 5,35686 | Hydrophobic | Pi-Alkyl |
| 4EY7 | α-terpineol | TRP286 | 5,09884 | Hydrophobic | Pi-Alkyl |
| 4EY7 | α-thujene | TRP86 | 3,80635 | Hydrophobic | Pi-Sigma |
| 4EY7 | α-thujene | TYR341 | 3,68662 | Hydrophobic | Pi-Sigma |
| 4EY7 | α-thujene | TYR337 | 4,30798 | Hydrophobic | Pi-Alkyl |
| 4EY7 | α-thujene | PHE338 | 4,91019 | Hydrophobic | Pi-Alkyl |
| 4EY7 | α-thujene | PHE338 | 3,99097 | Hydrophobic | Pi-Alkyl |
| 4EY7 | α-thujene | PHE338 | 4,66916 | Hydrophobic | Pi-Alkyl |
| 4EY7 | α-thujene | TYR341 | 4,51398 | Hydrophobic | Pi-Alkyl |
| 4EY7 | β-bourbonene | TRP86 | 3,78591 | Hydrophobic | Pi-Sigma |
| 4EY7 | β-bourbonene | TRP86 | 4,95316 | Hydrophobic | Pi-Alkyl |
| 4EY7 | β-bourbonene | TRP86 | 4,87206 | Hydrophobic | Pi-Alkyl |
| 4EY7 | β-bourbonene | TRP86 | 5,43907 | Hydrophobic | Pi-Alkyl |
| 4EY7 | β-bourbonene | TRP86 | 4,38943 | Hydrophobic | Pi-Alkyl |
| 4EY7 | β-bourbonene | TYR337 | 4,59961 | Hydrophobic | Pi-Alkyl |
| 4EY7 | β-caryophyllene | TYR337 | 4,48968 | Hydrophobic | Pi-Alkyl |
| 4EY7 | β-caryophyllene | PHE338 | 5,49479 | Hydrophobic | Pi-Alkyl |
| 4EY7 | β-caryophyllene | TYR341 | 5,42061 | Hydrophobic | Pi-Alkyl |
| 4EY7 | β-copaene | TYR337 | 3,82184 | Hydrophobic | Pi-Sigma |
| 4EY7 | β-copaene | PHE338 | 3,53307 | Hydrophobic | Pi-Sigma |
| 4EY7 | β-copaene | TRP86 | 4,61772 | Hydrophobic | Pi-Alkyl |
| 4EY7 | β-copaene | TYR337 | 4,90849 | Hydrophobic | Pi-Alkyl |
| 4EY7 | β-copaene | HIS447 | 5,2658 | Hydrophobic | Pi-Alkyl |
| 4EY7 | γ-muurolene | TRP86 | 4,41305 | Hydrophobic | Pi-Alkyl |
| 4EY7 | γ-muurolene | TRP86 | 4,4862 | Hydrophobic | Pi-Alkyl |
| 4EY7 | γ-muurolene | TRP86 | 4,27749 | Hydrophobic | Pi-Alkyl |
| 4EY7 | γ-muurolene | TYR337 | 4,85391 | Hydrophobic | Pi-Alkyl |
| 4EY7 | γ-muurolene | PHE338 | 5,1174 | Hydrophobic | Pi-Alkyl |
| 4EY7 | δ-cadinene | TYR341 | 3,72048 | Hydrophobic | Pi-Sigma |
| 4EY7 | δ-cadinene | TYR341 | 3,93924 | Hydrophobic | Pi-Sigma |
| 4EY7 | δ-cadinene | TRP286 | 3,94404 | Hydrophobic | Pi-Sigma |
| 4EY7 | δ-cadinene | TRP286 | 4,23509 | Hydrophobic | Pi-Alkyl |
| 4EY7 | δ-cadinene | TRP286 | 4,17709 | Hydrophobic | Pi-Alkyl |
| 4EY7 | δ-cadinene | TRP286 | 5,19906 | Hydrophobic | Pi-Alkyl |
| 4EY7 | δ-cadinol | TYR124 | 2,95461 | Hydrogen Bond | Conventional Hydrogen Bond |
| 4EY7 | δ-cadinol | TYR341 | 3,79022 | Hydrophobic | Pi-Sigma |
| 4EY7 | δ-cadinol | TRP86 | 3,58413 | Hydrophobic | Pi-Sigma |
| 4EY7 | δ-cadinol | TRP86 | 5,33285 | Hydrophobic | Pi-Alkyl |
| 4EY7 | δ-cadinol | PHE295 | 5,08671 | Hydrophobic | Pi-Alkyl |
| 4EY7 | δ-cadinol | PHE297 | 5,26704 | Hydrophobic | Pi-Alkyl |
| 4EY7 | δ-cadinol | PHE297 | 5,02526 | Hydrophobic | Pi-Alkyl |
| 4EY7 | δ-cadinol | TYR337 | 4,85912 | Hydrophobic | Pi-Alkyl |
| 4EY7 | δ-cadinol | PHE338 | 4,91357 | Hydrophobic | Pi-Alkyl |
| 4EY7 | δ-cadinol | HIS447 | 5,38502 | Hydrophobic | Pi-Alkyl |
| 4EY7 | δ-cadinol | HIS447 | 4,65968 | Hydrophobic | Pi-Alkyl |
| 5DYW | 1,8-cineol | TRP82 | 3,99913 | Hydrophobic | Pi-Sigma |
| 5DYW | 1,8-cineol | PHE329 | 3,84183 | Hydrophobic | Pi-Sigma |
| 5DYW | 1,8-cineol | ALA328 | 3,7962 | Hydrophobic | Alkyl |
| 5DYW | 1,8-cineol | ALA328 | 4,8777 | Hydrophobic | Alkyl |
| 5DYW | 1,8-cineol | ALA328 | 4,30696 | Hydrophobic | Alkyl |
| 5DYW | 1,8-cineol | PHE329 | 4,97095 | Hydrophobic | Pi-Alkyl |
| 5DYW | 1,8-cineol | TYR332 | 5,47189 | Hydrophobic | Pi-Alkyl |
| 5DYW | 1,8-cineol | TYR332 | 4,33596 | Hydrophobic | Pi-Alkyl |
| 5DYW | 1,8-cineol | HIS438 | 5,05149 | Hydrophobic | Pi-Alkyl |
| 5DYW | Bicyclogermacrene | TRP82 | 3,56938 | Hydrophobic | Pi-Sigma |
| 5DYW | Bicyclogermacrene | ALA328 | 5,37045 | Hydrophobic | Alkyl |
| 5DYW | Bicyclogermacrene | TRP82 | 4,29448 | Hydrophobic | Pi-Alkyl |
| 5DYW | Borneol | ASN228 | 2,94409 | Hydrogen Bond | Conventional Hydrogen Bond |
| 5DYW | Borneol | ASN228 | 3,29835 | Hydrogen Bond | Conventional Hydrogen Bond |
| 5DYW | Borneol | PRO401 | 4,76482 | Hydrophobic | Alkyl |
| 5DYW | Bornyl Acetate | HIS438 | 3,42602 | Hydrogen Bond | Carbon Hydrogen Bond |
| 5DYW | Bornyl Acetate | TRP82 | 3,69723 | Hydrophobic | Pi-Sigma |
| 5DYW | Bornyl Acetate | ALA328 | 5,10167 | Hydrophobic | Alkyl |
| 5DYW | Bornyl Acetate | TYR332 | 5,0129 | Hydrophobic | Pi-Alkyl |
| 5DYW | Calamenene | TRP82 | 3,86828 | Hydrophobic | Pi-Sigma |
| 5DYW | Calamenene | TRP82 | 4,09157 | Hydrophobic | Pi-Pi Stacked |
| 5DYW | Calamenene | TRP82 | 3,78517 | Hydrophobic | Pi-Pi Stacked |
| 5DYW | Calamenene | TRP82 | 4,13579 | Hydrophobic | Pi-Alkyl |
| 5DYW | Calamenene | TRP82 | 4,84838 | Hydrophobic | Pi-Alkyl |
| 5DYW | Calamenene | HIS438 | 4,59286 | Hydrophobic | Pi-Alkyl |
| 5DYW | Camphene | TRP82 | 3,55931 | Hydrophobic | Pi-Sigma |
| 5DYW | Camphene | ALA328 | 4,18444 | Hydrophobic | Alkyl |
| 5DYW | Camphene | TRP82 | 5,11101 | Hydrophobic | Pi-Alkyl |
| 5DYW | Camphene | TRP82 | 5,40242 | Hydrophobic | Pi-Alkyl |
| 5DYW | Camphene | TYR332 | 5,49624 | Hydrophobic | Pi-Alkyl |
| 5DYW | Camphene | TYR332 | 4,63184 | Hydrophobic | Pi-Alkyl |
| 5DYW | Camphor | TRP82 | 3,05587 | Hydrogen Bond | Conventional Hydrogen Bond |
| 5DYW | Camphor | TRP82 | 3,71805 | Hydrophobic | Pi-Sigma |
| 5DYW | Camphor | ALA328 | 4,00175 | Hydrophobic | Alkyl |
| 5DYW | Camphor | PHE329 | 5,38161 | Hydrophobic | Pi-Alkyl |
| 5DYW | Camphor | TYR332 | 4,85847 | Hydrophobic | Pi-Alkyl |
| 5DYW | Carvone | TRP82 | 3,7403 | Hydrophobic | Pi-Sigma |
| 5DYW | Carvone | TRP82 | 3,80069 | Hydrophobic | Pi-Alkyl |
| 5DYW | Carvone | TRP82 | 3,76144 | Hydrophobic | Pi-Alkyl |
| 5DYW | Caryophyllene Oxide | TRP82 | 3,89818 | Hydrophobic | Pi-Sigma |
| 5DYW | Caryophyllene Oxide | TYR332 | 5,09698 | Hydrophobic | Pi-Alkyl |
| 5DYW | Epi-Cubebol | TRP82 | 3,55408 | Hydrogen Bond | Pi-Donor Hydrogen Bond |
| 5DYW | Epi-Cubebol | TYR332 | 3,72879 | Hydrophobic | Pi-Sigma |
| 5DYW | Epi-Cubebol | TRP82 | 5,22223 | Hydrophobic | Pi-Alkyl |
| 5DYW | Germacrene-D-4-ol | TRP82 | 3,86082 | Hydrophobic | Pi-Alkyl |
| 5DYW | Germacrene-D-4-ol | TRP82 | 4,92424 | Hydrophobic | Pi-Alkyl |
| 5DYW | Germacrene-D-4-ol | TRP82 | 4,51285 | Hydrophobic | Pi-Alkyl |
| 5DYW | Germacrene-D-4-ol | TRP82 | 4,01951 | Hydrophobic | Pi-Alkyl |
| 5DYW | Hexahydrofarnesylacetone | TRP82 | 3,69735 | Hydrophobic | Pi-Sigma |
| 5DYW | Hexahydrofarnesylacetone | TRP82 | 5,46764 | Hydrophobic | Pi-Alkyl |
| 5DYW | Ledol | THR120 | 2,76264 | Hydrogen Bond | Conventional Hydrogen Bond |
| 5DYW | Ledol | GLY116 | 3,71651 | Hydrogen Bond | Carbon Hydrogen Bond |
| 5DYW | Ledol | TRP82 | 3,58164 | Hydrophobic | Pi-Sigma |
| 5DYW | Ledol | TRP82 | 4,232 | Hydrophobic | Pi-Alkyl |
| 5DYW | Ledol | TRP82 | 5,23745 | Hydrophobic | Pi-Alkyl |
| 5DYW | Ledol | TRP82 | 4,96451 | Hydrophobic | Pi-Alkyl |
| 5DYW | Ledol | TRP82 | 4,64387 | Hydrophobic | Pi-Alkyl |
| 5DYW | Limonene | TRP82 | 3,97753 | Hydrophobic | Pi-Alkyl |
| 5DYW | Limonene | TRP82 | 3,76514 | Hydrophobic | Pi-Alkyl |
| 5DYW | Limonene | TRP82 | 4,374 | Hydrophobic | Pi-Alkyl |
| 5DYW | Limonene | HIS438 | 5,42057 | Hydrophobic | Pi-Alkyl |
| 5DYW | Linalool | GLU197 | 2,8479 | Hydrogen Bond | Conventional Hydrogen Bond |
| 5DYW | Linalool | TRP82 | 3,72928 | Hydrogen Bond | Pi-Donor Hydrogen Bond |
| 5DYW | Linalool | ALA328 | 4,34544 | Hydrophobic | Alkyl |
| 5DYW | Linalool | ALA328 | 4,34192 | Hydrophobic | Alkyl |
| 5DYW | Linalool | TRP82 | 4,39105 | Hydrophobic | Pi-Alkyl |
| 5DYW | Linalool | TRP82 | 4,66359 | Hydrophobic | Pi-Alkyl |
| 5DYW | Linalool | TRP82 | 5,00384 | Hydrophobic | Pi-Alkyl |
| 5DYW | Linalool | TRP82 | 5,12802 | Hydrophobic | Pi-Alkyl |
| 5DYW | Linalool | TRP82 | 4,4752 | Hydrophobic | Pi-Alkyl |
| 5DYW | Linalool | TRP430 | 5,11799 | Hydrophobic | Pi-Alkyl |
| 5DYW | Myrcene | TRP82 | 3,73512 | Hydrophobic | Pi-Sigma |
| 5DYW | Myrcene | ALA328 | 3,55651 | Hydrophobic | Alkyl |
| 5DYW | Myrcene | TRP82 | 3,65902 | Hydrophobic | Pi-Alkyl |
| 5DYW | Myrcene | TRP82 | 5,09925 | Hydrophobic | Pi-Alkyl |
| 5DYW | Myrcene | TRP82 | 3,95469 | Hydrophobic | Pi-Alkyl |
| 5DYW | Myrcene | TRP82 | 4,42018 | Hydrophobic | Pi-Alkyl |
| 5DYW | Myrcene | TYR332 | 4,93514 | Hydrophobic | Pi-Alkyl |
| 5DYW | Myrcene | TRP430 | 4,67 | Hydrophobic | Pi-Alkyl |
| 5DYW | Myrcene | TRP430 | 4,00657 | Hydrophobic | Pi-Alkyl |
| 5DYW | Myrtenal | TRP82 | 3,014 | Hydrogen Bond | Conventional Hydrogen Bond |
| 5DYW | Myrtenal | TRP430 | 3,29549 | Hydrogen Bond | Conventional Hydrogen Bond |
| 5DYW | Myrtenal | PHE329 | 3,98862 | Hydrophobic | Pi-Sigma |
| 5DYW | Myrtenal | TYR332 | 3,99402 | Hydrophobic | Pi-Sigma |
| 5DYW | Myrtenal | ALA328 | 4,53316 | Hydrophobic | Alkyl |
| 5DYW | Myrtenal | TRP82 | 5,44918 | Hydrophobic | Pi-Alkyl |
| 5DYW | Myrtenol | TRP82 | 2,99417 | Hydrogen Bond | Conventional Hydrogen Bond |
| 5DYW | Myrtenol | TRP430 | 3,31684 | Hydrogen Bond | Conventional Hydrogen Bond |
| 5DYW | Myrtenol | PHE329 | 3,96293 | Hydrophobic | Pi-Sigma |
| 5DYW | Myrtenol | ALA328 | 4,32156 | Hydrophobic | Alkyl |
| 5DYW | Myrtenol | TYR332 | 5,36892 | Hydrophobic | Pi-Alkyl |
| 5DYW | p-Cymene | TRP82 | 3,88361 | Hydrophobic | Pi-Pi Stacked |
| 5DYW | p-Cymene | TRP82 | 3,74237 | Hydrophobic | Pi-Pi Stacked |
| 5DYW | p-Cymene | ALA328 | 4,0077 | Hydrophobic | Alkyl |
| 5DYW | p-Cymene | MET437 | 5,34977 | Hydrophobic | Alkyl |
| 5DYW | p-Cymene | TRP82 | 4,45761 | Hydrophobic | Pi-Alkyl |
| 5DYW | p-Cymene | TRP82 | 5,13961 | Hydrophobic | Pi-Alkyl |
| 5DYW | p-Cymene | TYR440 | 5,35223 | Hydrophobic | Pi-Alkyl |
| 5DYW | Phytol | SER198 | 2,91473 | Hydrogen Bond | Conventional Hydrogen Bond |
| 5DYW | Phytol | PHE329 | 5,04488 | Hydrophobic | Pi-Alkyl |
| 5DYW | Pulegone | TRP82 | 3,69795 | Hydrophobic | Pi-Sigma |
| 5DYW | Pulegone | ALA328 | 3,68371 | Hydrophobic | Alkyl |
| 5DYW | Pulegone | TYR332 | 4,98244 | Hydrophobic | Pi-Alkyl |
| 5DYW | Pulegone | TRP430 | 5,03657 | Hydrophobic | Pi-Alkyl |
| 5DYW | Pulegone | TRP430 | 4,44676 | Hydrophobic | Pi-Alkyl |
| 5DYW | Sabinene | ALA328 | 5,35935 | Hydrophobic | Alkyl |
| 5DYW | Sabinene | PHE329 | 5,30074 | Hydrophobic | Pi-Alkyl |
| 5DYW | Sabinene | TYR332 | 4,3185 | Hydrophobic | Pi-Alkyl |
| 5DYW | Sabinene | TYR332 | 3,54819 | Hydrophobic | Pi-Alkyl |
| 5DYW | Spathulenol | PHE329 | 3,95279 | Hydrophobic | Pi-Sigma |
| 5DYW | Spathulenol | ALA328 | 3,75835 | Hydrophobic | Alkyl |
| 5DYW | Spathulenol | ALA328 | 5,35588 | Hydrophobic | Alkyl |
| 5DYW | Spathulenol | TRP82 | 4,37352 | Hydrophobic | Pi-Alkyl |
| 5DYW | Spathulenol | TRP82 | 4,34153 | Hydrophobic | Pi-Alkyl |
| 5DYW | Spathulenol | TRP82 | 4,87445 | Hydrophobic | Pi-Alkyl |
| 5DYW | Spathulenol | TRP82 | 4,86172 | Hydrophobic | Pi-Alkyl |
| 5DYW | Spathulenol | TYR332 | 4,66777 | Hydrophobic | Pi-Alkyl |
| 5DYW | Spathulenol | HIS438 | 5,34916 | Hydrophobic | Pi-Alkyl |
| 5DYW | T-Cadinol | ALA328 | 4,10985 | Hydrophobic | Alkyl |
| 5DYW | T-Cadinol | MET437 | 5,24565 | Hydrophobic | Alkyl |
| 5DYW | T-Cadinol | TRP82 | 4,2086 | Hydrophobic | Pi-Alkyl |
| 5DYW | T-Cadinol | TRP82 | 3,94684 | Hydrophobic | Pi-Alkyl |
| 5DYW | T-Cadinol | TRP82 | 4,333 | Hydrophobic | Pi-Alkyl |
| 5DYW | T-Cadinol | TRP82 | 4,60227 | Hydrophobic | Pi-Alkyl |
| 5DYW | T-Cadinol | TRP82 | 4,06524 | Hydrophobic | Pi-Alkyl |
| 5DYW | T-Cadinol | TRP82 | 5,05695 | Hydrophobic | Pi-Alkyl |
| 5DYW | T-Cadinol | HIS438 | 5,15498 | Hydrophobic | Pi-Alkyl |
| 5DYW | T-Cadinol | TYR440 | 5,15085 | Hydrophobic | Pi-Alkyl |
| 5DYW | Terpinen-4-ol | TRP82 | 3,63332 | Hydrogen Bond | Carbon Hydrogen Bond |
| 5DYW | Terpinen-4-ol | TYR332 | 3,91454 | Hydrophobic | Pi-Sigma |
| 5DYW | Terpinen-4-ol | ALA328 | 4,42233 | Hydrophobic | Alkyl |
| 5DYW | Terpinen-4-ol | PHE329 | 4,52086 | Hydrophobic | Pi-Alkyl |
| 5DYW | Terpinen-4-ol | PHE329 | 4,60722 | Hydrophobic | Pi-Alkyl |
| 5DYW | Terpinen-4-ol | TYR332 | 4,97274 | Hydrophobic | Pi-Alkyl |
| 5DYW | Tricosane | TRP231 | 3,66439 | Hydrophobic | Pi-Sigma |
| 5DYW | Tricosane | TRP231 | 3,61891 | Hydrophobic | Pi-Sigma |
| 5DYW | Tricosane | TRP231 | 4,81991 | Hydrophobic | Pi-Alkyl |
| 5DYW | Tricosane | TRP231 | 4,53022 | Hydrophobic | Pi-Alkyl |
| 5DYW | Tricosane | TYR332 | 5,3923 | Hydrophobic | Pi-Alkyl |
| 5DYW | Viridiflorol | HIS438 | 3,5956 | Hydrogen Bond | Carbon Hydrogen Bond |
| 5DYW | Viridiflorol | TRP82 | 3,7438 | Hydrophobic | Pi-Sigma |
| 5DYW | Viridiflorol | TRP82 | 5,06485 | Hydrophobic | Pi-Alkyl |
| 5DYW | Viridiflorol | TRP82 | 4,31268 | Hydrophobic | Pi-Alkyl |
| 5DYW | α-cadinol | HIS438 | 3,16287 | Hydrogen Bond | Conventional Hydrogen Bond |
| 5DYW | α-cadinol | TRP82 | 4,02811 | Hydrophobic | Pi-Alkyl |
| 5DYW | α-cadinol | TRP82 | 4,77318 | Hydrophobic | Pi-Alkyl |
| 5DYW | α-cadinol | TRP82 | 5,20202 | Hydrophobic | Pi-Alkyl |
| 5DYW | α-cadinol | TRP82 | 3,82005 | Hydrophobic | Pi-Alkyl |
| 5DYW | α-cadinol | TRP82 | 4,3272 | Hydrophobic | Pi-Alkyl |
| 5DYW | α-cadinol | HIS438 | 5,37038 | Hydrophobic | Pi-Alkyl |
| 5DYW | α-copaene | TRP82 | 3,80618 | Hydrophobic | Pi-Sigma |
| 5DYW | α-copaene | TYR332 | 3,98186 | Hydrophobic | Pi-Sigma |
| 5DYW | α-copaene | ALA328 | 5,00594 | Hydrophobic | Alkyl |
| 5DYW | α-copaene | TRP82 | 4,46436 | Hydrophobic | Pi-Alkyl |
| 5DYW | α-copaene | TRP82 | 4,73648 | Hydrophobic | Pi-Alkyl |
| 5DYW | α-copaene | TRP82 | 5,17435 | Hydrophobic | Pi-Alkyl |
| 5DYW | α-copaene | TRP82 | 4,89112 | Hydrophobic | Pi-Alkyl |
| 5DYW | α-copaene | HIS438 | 4,50782 | Hydrophobic | Pi-Alkyl |
| 5DYW | α-cubebene | TRP82 | 3,61301 | Hydrophobic | Pi-Sigma |
| 5DYW | α-cubebene | PHE329 | 3,76003 | Hydrophobic | Pi-Sigma |
| 5DYW | α-cubebene | TRP82 | 4,68228 | Hydrophobic | Pi-Alkyl |
| 5DYW | α-cubebene | TRP82 | 4,04871 | Hydrophobic | Pi-Alkyl |
| 5DYW | α-cubebene | TRP82 | 4,36575 | Hydrophobic | Pi-Alkyl |
| 5DYW | α-cubebene | TRP82 | 4,38502 | Hydrophobic | Pi-Alkyl |
| 5DYW | α-cubebene | HIS438 | 5,21858 | Hydrophobic | Pi-Alkyl |
| 5DYW | α-humulene | PHE329 | 3,88192 | Hydrophobic | Pi-Sigma |
| 5DYW | α-humulene | TRP82 | 3,74327 | Hydrophobic | Pi-Sigma |
| 5DYW | α-humulene | TRP82 | 4,58863 | Hydrophobic | Pi-Alkyl |
| 5DYW | α-humulene | TRP82 | 4,68015 | Hydrophobic | Pi-Alkyl |
| 5DYW | α-humulene | TRP82 | 5,33556 | Hydrophobic | Pi-Alkyl |
| 5DYW | α-humulene | HIS438 | 4,81689 | Hydrophobic | Pi-Alkyl |
| 5DYW | α-muurolene | TRP82 | 3,76634 | Hydrophobic | Pi-Sigma |
| 5DYW | α-muurolene | TRP82 | 3,4588 | Hydrophobic | Pi-Sigma |
| 5DYW | α-muurolene | ALA328 | 4,17561 | Hydrophobic | Alkyl |
| 5DYW | α-muurolene | MET437 | 5,09386 | Hydrophobic | Alkyl |
| 5DYW | α-muurolene | ALA328 | 4,82692 | Hydrophobic | Alkyl |
| 5DYW | α-muurolene | TRP82 | 4,32803 | Hydrophobic | Pi-Alkyl |
| 5DYW | α-muurolene | TRP82 | 4,26725 | Hydrophobic | Pi-Alkyl |
| 5DYW | α-muurolene | TRP82 | 5,07892 | Hydrophobic | Pi-Alkyl |
| 5DYW | α-muurolene | TRP82 | 4,47538 | Hydrophobic | Pi-Alkyl |
| 5DYW | α-muurolene | HIS438 | 5,23931 | Hydrophobic | Pi-Alkyl |
| 5DYW | α-muurolene | HIS438 | 5,2159 | Hydrophobic | Pi-Alkyl |
| 5DYW | α-muurolene | TYR440 | 4,96694 | Hydrophobic | Pi-Alkyl |
| 5DYW | α-phellandrene | TRP82 | 3,98889 | Hydrophobic | Pi-Alkyl |
| 5DYW | α-phellandrene | TRP82 | 5,15447 | Hydrophobic | Pi-Alkyl |
| 5DYW | α-phellandrene | TRP82 | 3,77812 | Hydrophobic | Pi-Alkyl |
| 5DYW | α-phellandrene | TRP82 | 4,27576 | Hydrophobic | Pi-Alkyl |
| 5DYW | α-phellandrene | HIS438 | 5,38684 | Hydrophobic | Pi-Alkyl |
| 5DYW | α-terpineol | PRO285 | 2,97536 | Hydrogen Bond | Conventional Hydrogen Bond |
| 5DYW | α-terpineol | SER287 | 2,81035 | Hydrogen Bond | Conventional Hydrogen Bond |
| 5DYW | α-terpineol | SER287 | 3,19129 | Hydrogen Bond | Conventional Hydrogen Bond |
| 5DYW | α-terpineol | TRP231 | 3,8602 | Hydrophobic | Pi-Sigma |
| 5DYW | α-terpineol | TRP231 | 3,61611 | Hydrophobic | Pi-Sigma |
| 5DYW | α-terpineol | LEU286 | 5,4634 | Hydrophobic | Alkyl |
| 5DYW | α-terpineol | LEU286 | 5,31295 | Hydrophobic | Alkyl |
| 5DYW | α-terpineol | PHE329 | 5,45902 | Hydrophobic | Pi-Alkyl |
| 5DYW | α-terpineol | PHE329 | 5,32071 | Hydrophobic | Pi-Alkyl |
| 5DYW | α-terpineol | PHE398 | 4,68668 | Hydrophobic | Pi-Alkyl |
| 5DYW | α-terpineol | HIS438 | 5,25802 | Hydrophobic | Pi-Alkyl |
| 5DYW | α-thujene | TRP82 | 3,84342 | Hydrophobic | Pi-Sigma |
| 5DYW | α-thujene | ALA328 | 4,00806 | Hydrophobic | Alkyl |
| 5DYW | α-thujene | TRP82 | 5,1191 | Hydrophobic | Pi-Alkyl |
| 5DYW | α-thujene | TRP82 | 3,67932 | Hydrophobic | Pi-Alkyl |
| 5DYW | α-thujene | TRP82 | 4,57245 | Hydrophobic | Pi-Alkyl |
| 5DYW | α-thujene | TRP82 | 4,29128 | Hydrophobic | Pi-Alkyl |
| 5DYW | α-thujene | HIS438 | 5,04946 | Hydrophobic | Pi-Alkyl |
| 5DYW | α-thujene | HIS438 | 4,58678 | Hydrophobic | Pi-Alkyl |
| 5DYW | β-bourbonene | TRP82 | 4,58393 | Hydrophobic | Pi-Alkyl |
| 5DYW | β-bourbonene | TRP82 | 4,24966 | Hydrophobic | Pi-Alkyl |
| 5DYW | β-bourbonene | TRP82 | 3,96509 | Hydrophobic | Pi-Alkyl |
| 5DYW | β-caryophyllene | TRP82 | 3,61992 | Hydrophobic | Pi-Sigma |
| 5DYW | β-caryophyllene | TRP82 | 3,71998 | Hydrophobic | Pi-Sigma |
| 5DYW | β-copaene | TRP82 | 3,78373 | Hydrophobic | Pi-Sigma |
| 5DYW | β-copaene | TRP82 | 4,11261 | Hydrophobic | Pi-Alkyl |
| 5DYW | β-copaene | TRP82 | 5,37502 | Hydrophobic | Pi-Alkyl |
| 5DYW | β-copaene | TRP82 | 4,54143 | Hydrophobic | Pi-Alkyl |
| 5DYW | β-copaene | HIS438 | 5,39021 | Hydrophobic | Pi-Alkyl |
| 5DYW | β-copaene | HIS438 | 5,21326 | Hydrophobic | Pi-Alkyl |
| 5DYW | γ-muurolene | TRP82 | 3,73608 | Hydrophobic | Pi-Sigma |
| 5DYW | γ-muurolene | TRP82 | 3,43837 | Hydrophobic | Pi-Sigma |
| 5DYW | γ-muurolene | ALA328 | 4,19038 | Hydrophobic | Alkyl |
| 5DYW | γ-muurolene | TRP82 | 5,12951 | Hydrophobic | Pi-Alkyl |
| 5DYW | γ-muurolene | TRP82 | 4,48179 | Hydrophobic | Pi-Alkyl |
| 5DYW | γ-muurolene | PHE329 | 4,91046 | Hydrophobic | Pi-Alkyl |
| 5DYW | γ-muurolene | HIS438 | 5,45544 | Hydrophobic | Pi-Alkyl |
| 5DYW | γ-muurolene | HIS438 | 4,88752 | Hydrophobic | Pi-Alkyl |
| 5DYW | δ-cadinene | TRP82 | 3,64612 | Hydrophobic | Pi-Sigma |
| 5DYW | δ-cadinene | ALA328 | 4,03795 | Hydrophobic | Alkyl |
| 5DYW | δ-cadinene | MET437 | 5,32181 | Hydrophobic | Alkyl |
| 5DYW | δ-cadinene | ALA328 | 4,62416 | Hydrophobic | Alkyl |
| 5DYW | δ-cadinene | TRP82 | 4,03116 | Hydrophobic | Pi-Alkyl |
| 5DYW | δ-cadinene | TRP82 | 4,11446 | Hydrophobic | Pi-Alkyl |
| 5DYW | δ-cadinene | TRP82 | 4,83818 | Hydrophobic | Pi-Alkyl |
| 5DYW | δ-cadinene | TRP82 | 4,48235 | Hydrophobic | Pi-Alkyl |
| 5DYW | δ-cadinene | TRP82 | 4,89238 | Hydrophobic | Pi-Alkyl |
| 5DYW | δ-cadinene | TRP430 | 4,51457 | Hydrophobic | Pi-Alkyl |
| 5DYW | δ-cadinene | TRP430 | 4,46823 | Hydrophobic | Pi-Alkyl |
| 5DYW | δ-cadinol | TRP82 | 3,48038 | Hydrophobic | Pi-Sigma |
| 5DYW | δ-cadinol | TRP82 | 4,07175 | Hydrophobic | Pi-Alkyl |
| 5DYW | δ-cadinol | TRP82 | 4,56714 | Hydrophobic | Pi-Alkyl |
| 5DYW | δ-cadinol | TRP82 | 5,40799 | Hydrophobic | Pi-Alkyl |
| 5DYW | δ-cadinol | HIS438 | 5,2364 | Hydrophobic | Pi-Alkyl |
